# Cationic Cycloheptatrienyl Cyclopentadienyl Manganese Sandwich Complexes: Tromancenium Explored with High-Power LED Photosynthesis

**DOI:** 10.1021/acs.organomet.1c00376

**Published:** 2021-07-27

**Authors:** Reinhard Basse, Stefan Vanicek, Thomas Höfer, Holger Kopacka, Klaus Wurst, Thomas Müller, Heidi A. Schwartz, Selina Olthof, Larissa A. Casper, Moritz Nau, Rainer F. Winter, Maren Podewitz, Benno Bildstein

**Affiliations:** # Institute of General, Inorganic and Theoretical Chemistry, Center for Chemistry and Biomedicine, University of Innsbruck, Innrain 80-82, 6020 Innsbruck, Austria; ◊ Institute of Organic Chemistry, Center for Chemistry and Biomedicine, University of Innsbruck, Innrain 80-82, 6020 Innsbruck, Austria; & Department of Chemistry, University of Cologne, Greinstrasse 4-6, 50939 Köln, Germany; ÷ Department of Chemistry, University of Konstanz, Universitätsstrasse 10, 78457 Konstanz, Germany

## Abstract

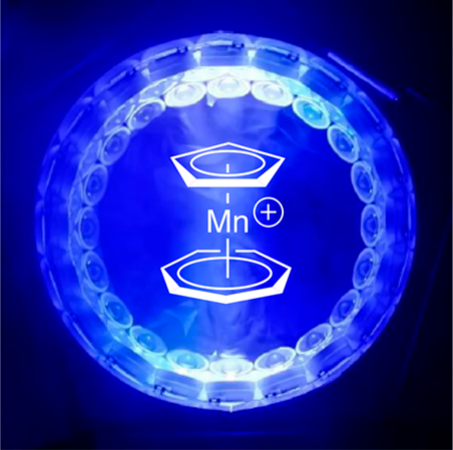

In this contribution, we revisit the neglected and forgotten cationic, air-stable, 18-valence electron, heteroleptic sandwich complex (cycloheptatrienyl)(cyclopentadienyl)manganese, which was reported independently by Fischer and by Pauson about 50 years ago. Using advanced high-power LED photochemical synthesis, an expedient rapid access to the parent complex and to functionalized derivatives with alkyl, carboxymethyl, bromo, and amino substituents was developed. A thorough study of these “tromancenium” salts by a range of spectroscopic techniques (^1^H/^13^C/^55^Mn-NMR, IR, UV–vis, HRMS, XRD, XPS, EPR), cyclic voltammetry (CV), and quantum chemical calculations (DFT) shows that these manganese sandwich complexes are unique metallocenes with quite different chemical and physical properties in comparison to those of isoelectronic cobaltocenium salts or (cycloheptatrienyl)(cyclopentadienyl) sandwich complexes of the early transition metals. Electrochemically, all tromancenium ions undergo a chemically partially reversible oxidation and a chemically irreversible reduction at half-wave or peak potentials that respond to the substituents at the Cp deck. As exemplarily shown for the parent tromancenium ion, the product generated during the irreversible reduction process reverts at least partially to the starting material upon reoxidation. Quantum-chemical calculations of the parent tromancenium salt indicate that metal–ligand bonding is distinctly weaker for the cycloheptatrienyl ligand in comparison to that of the cyclopentadienyl ligand. Both the HOMO and the LUMO are metal and cycloheptatrienyl-ligand centered, indicating that chemical reactions will occur either metal-based or at the seven-membered ring, but not on the cyclopentadienyl ligand.

## Introduction

Among the huge number of metal sandwich complexes known in organometallic chemistry, the class of heteroleptic (cycloheptatrienyl)(cyclopentadienyl) metal complexes [(Cht)M(Cp)]^±*n*^ constitutes an interesting family of compounds, mostly restricted to early transition metals, as shown by the work of Green,^1^ Elschenbroich,^2^ Tamm,^3^ and Braunschweig.^4^Table 1 summarizes the known representatives commonly called “tro-met-cenes” where “tro” stands for tropylium (C_7_H_7_)^+^, “met” for the metal center, and “cene” for a cyclopentadienide-containing sandwich complex. For example, the corresponding titanium, vanadium, or chromium species are called troticene, trovacene, and trochrocene, respectively. Whereas the chemistry of the group 4, 5, and 6 trometcenes has been and still is frequently studied,^3,4^ the representatives of manganese and ruthenium seem to be totally neglected sandwich complexes. This is rather surprising because these complexes are air-stable, redox-responsive 18-valence electron species, isoelectronic to ferrocene, with potentially similar useful properties and applications. We have previously contributed to this area with a publication on tropolone-based trorucenium metalloligands^5^ and would like to report here on new advances in tromancenium chemistry. The first and only representatives of simple tromancenium complexes were reported half a century ago independently by Fischer^6^ and by Pauson,^7^ but surprisingly, no further publications on this subject have appeared since then. Tromancenium salts are interesting monocationic sandwich compounds, isoelectronic to cobaltocenium salts,^8^ with prospective similarly advantageous properties, including high polarity, good solubility in polar solvents, redox-activity, and nontoxic character. All of these are desirable features in green chemistry, medicinal chemistry, and electrocatalysis. In addition, this research is a contribution to current efforts in homogeneous catalysis focusing on Earth-abundant, first-row transition metals like manganese, iron, and cobalt (so-called “base metals”) as alternatives to rare and expensive 4d and 5d elements.^9^

**Table 1:** Overview of Cycloheptatrienyl Cyclopentadienyl Transition Metal Sandwich Complexes^a^

group 4	group 5	group 6	group 7	group 8
[(Cht)**Ti**(Cp)]^−^	[(Cht)**V**(Cp)]^−^	[(Cht)**Cr**(Cp)]^−^		
[(Cht)**Ti**(Cp)]	[(Cht)**V**(Cp)]	[(Cht)**Cr**(Cp)]	[(Cht)**Mn**(Cp)]^+^	
	[(Cht)**V**(Cp)]^+^	[(Cht)**Cr**(Cp)]^+^		
[(Cht)**Zr**(Cp)]	[(Cht)**Nb**(Cp)]	[(Cht)**Mo**(Cp)]		[(Cht)**Ru**(Cp)]^2+^
[(Cht)**Hf**(Cp)]	[(Cht)**Ta**(Cp)]	[(Cht)**W**(Cp)]		

a Cht = η^7^-cycloheptatrienyl, Cp = η^5^-cyclopentadienyl.

## Results and Discussion

### State of Knowledge on the Chemistry of Tromancenium Salts

**Scheme 1:**
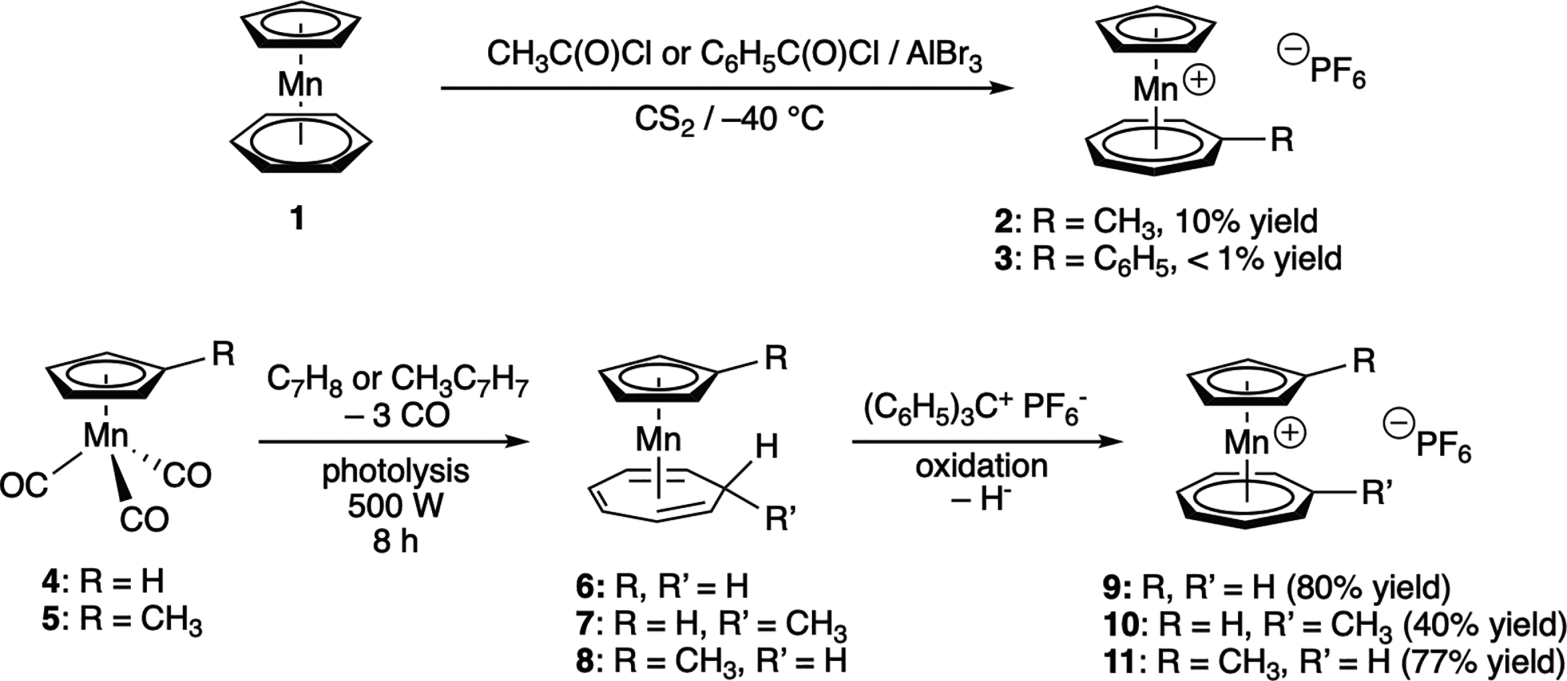
Fischer’s (Top) and Pauson’s (Bottom) Synthesis of the First Tromancenium Derivatives

Tromancenium is a “forgotten” stable π-sandwich species, most likely because it is quite difficult to prepare. In 1966, Fischer and Breitschaft^6^ obtained by accident and in poor yield 1-methyltromancenium hexafluoridophosphate (**2**) and 1-phenyltromancenium hexafluoridophosphate (**3**), respectively, when they attempted Friedel–Crafts acylation of (benzene)(cyclopentadienyl)manganese(I) (**1**) (Scheme 1). Chemically, this is an interesting, unusual ring-expansion of benzene to a seven-membered π-conjugated cyclic ligand by formal electrophilic attack of [CH_3_–C^+^] or [C_6_H_5_–C^+^], which indicates a high thermodynamic stability of the tromancenium moiety. From a synthetic chemist’s viewpoint, this reaction is of no further value, also because the starting complex **1** is accessible in only less than 3% yield.^6^ A few years later, in 1973, Pauson and Segal^7^ reported a clean and straightforward synthesis of the parent tromancenium hexafluoridophosphate (**9**, 80% isolated yield), of 1-methyltromancenium hexafluoridophosphate (**10**, 40% yield) and of 8-methyltromancenium hexafluoridophosphate (**11**, 77% yield) by photochemical displacement of all three CO ligands of tricarbonyl(cyclopentadienyl)manganese (**4**) or tricarbonyl(methylcyclopentadienyl)manganese (**5**) with cycloheptatriene or methylcycloheptatriene to the corresponding cyclopentadienyl-triene-Mn(I) complexes **6**–**8**, followed by oxidation/hydride abstraction with tritylium hexafluoridophosphate (Scheme 1). This photochemical protocol looks at first sight very promising. However, in reality, it is rather difficult to perform because intermediates **6**–**8** are highly air-sensitive and—in practice even more relevant—acceptable yields are only obtained using a falling film photoreactor,^7c^ which is an expensive specialized equipment not available in most laboratories. The tromancenium salts **2**, **9**, **10**, and **11** have been described as air-stable, pink, polar, diamagnetic compounds and were spectroscopically characterized by IR and ^1^H NMR spectroscopy only.^6,7^ In particular, no single-crystal structure analyses or electrochemical properties have been reported.

### High-Power LED Photochemical Synthesis

**Scheme 2:**
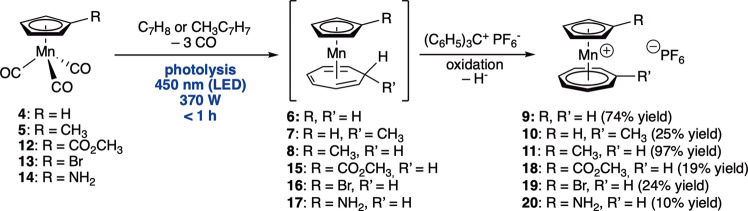
High-Power LED Photosynthesis of Tromancenium Salts

In comparison to common photochemical synthesis using standard medium-pressure mercury lamps, modern light-emitting diodes (LEDs) provide improved conditions using high-intensity monochromatic light that excites one specific transition and hence has the propensity to selectively activate a desired chemical process. We used here a custom-made photochemical reactor called “Solar Light Lab Luminaire” (see the Experimental Section) based on high-power LEDs developed in collaboration with the Austrian company Bartenbach.^10^ This apparatus proved extremely useful for our purposes. It allows very convenient, rapid, selective, high-power photochemical synthesis (270–370 W per channel) for five wavelengths from the UV to the visible part of the spectrum (UV-1:360 nm, UV-2:405 nm, blue: 450 nm, green: 535 nm, cold white (5000 K)). By using blue light and an irradiation period of only 1 h or less, we are able to obtain the intermediate **6**, **7**, or **8** in a straightforward manner (Scheme 2). Further reaction with tritylium hexafluoridophosphate afforded the desired tromancenium salts **9**, **10**, and **11** in 97–25% isolated yield on a 1 g scale, corresponding to an overall quantum yield of 2.9% (for **9**), 5.3% (for **10**), and 3.2% (for **11**), respectively. In the case of 1-methyl-substituted tromancenium **10**, the chemical yield dropped to 10% due to competing cycloaddition reactions of the methylcycloheptatriene ligand. This is a general problem, as attempted reactions with other substituted cycloheptatrienes like cyano or ethynyl cycloheptatriene failed to provide the desired cycloheptatriene complexes. Hence, mostly 8-substituted tromancenium compounds are accessible by this procedure. Nevertheless, this LED-based approach is clearly much more convenient than Pauson’s original procedure using a traditional medium-pressure mercury lamp in a falling film photoreactor with irradiation times of 8 h.^7^

Having methylated tromancenium salts **10** and **11** in hand, we anticipated that aromatic side-chain oxidation with aqueous KMnO_4_ would cleanly afford the corresponding carboxylic acids, in analogy to such established reactivity in cobaltocenium chemistry.^8a^ Surprisingly, only unreacted methylated starting materials **10** and **11** were recovered after the reactions, although discoloration of the purple KMnO_4_ solution and concomitant formation of MnO_2_ precipitate were observed. Interestingly, the same result was also obtained with nonmethylated tromancenium salt **9**, suggesting that tromancenium-catalyzed decomposition of permanganate took place instead of the desired aromatic side-chain oxidation. Attempts with various other oxidizing agents gave the same negative result.

An alternative synthetic access to tromancenium carboxylic acids was therefore required. Starting from cymantrenecarboxylic acid,^11^ photochemical substitution of the carbonyl ligands by cycloheptatriene followed by oxidation/hydride removal with tritylium was attempted, but without any success, suggesting that the carboxylic acid functionality is incompatible with our photochemical reaction conditions. To circumvent this problem, the methyl ester of cymantrenecarboxylic acid (**12**)^12^ as an O-protected synthon was used, and indeed, the corresponding 8-methoxycarbonyltromancenium hexafluoridophosphate (**18**) was obtained in 19% isolated yield (Scheme 2). Deprotection by alkaline hydrolysis to the desired tromancenium-8-carboxylic acid was attempted, but unfortunately, under these highly basic conditions only decomposition was observed. Hence, at the current state, tromanceniumcarboxylic acid remains an elusive target compound that needs to be made by other methods.

Next, we were interested if cymantrene derivatives containing other valuable functional groups are also suitable for the photochemical conversion to tromancenium salts. Satisfyingly, with bromocymantrene (**13**)^13^ the corresponding 8-bromotromancenium complex **19** was obtained in 24% chemical and in 2.7% quantum yield. In practice, the photochemical reaction of halogenated cymantrene derivatives needed careful optimization of the irradiation times: for bromocymantrene a very short duration of 2.30 min proved optimal whereas attempted analogous reaction with iodocymantrene^14^ resulted in complete decomposition, even after short irradiation times. Not unexpectedly, halogenated precursors are quite labile under these harsh photochemical conditions. A further very useful functional group is the amino group. Interestingly, even with this reactive substituent, the desired 8-aminotromancenium complex **20** was obtained on a 0.50 mmol scale by irradiation of aminocymantrene (**14**)^15^ for 3.40 min followed by oxidation with tritylium in 9.5% chemical and in 8.4% quantum yield.

A general and useful derivatization strategy of cationic sandwich metal complexes (for example cobaltocenium salts) is nucleophilic exoaddition of various nucleophiles to provide neutral, functionalized endohydride derivatives that may subsequently be oxidized with tritylium to valuable substituted sandwich metal cations.^8a,16^ Attempted reactions of tromancenium complex **9** with a range of carbon, nitrogen, or oxygen nucleophiles resulted, unfortunately, only in complete decomposition of the manganese sandwich complex, indicating that tromancenium salts are surprisingly labile in comparison to other cationic metallocenes, due to rather weak bonding of the cycloheptatrienyl ligand (vide infra).

To summarize this synthetic section, high-power LED photochemistry of metal carbonyl complexes is convenient, fast, and superior to standard methodology. For tromancenium compounds, LED photochemistry with blue light proved very useful and provided rapid access to several target complexes, including highly valuable functionalized derivatives. Preferred starting materials are functionalized cymantrene complexes^17^ that are known in a large structural variety, thereby opening up interesting options for future developments in this class of compounds. On the other hand, tromancenium salts are quite susceptible to nucleophile-triggered degradation, thereby prohibiting derivatization methods involving nucleophilic attack on the cycloheptatrienylium ligand, which are well established for other cationic sandwich complexes.^16^

### Physical, Structural, And Spectroscopic Properties

Tromancenium salts **9**–**11** and **18**–**20** are air-stable, red crystalline solids with high melting points in the range of 206–256 °C (see the Experimental Section). They are soluble in polar solvents like acetonitrile, acetone, dimethylformamide, and dimethyl sulfoxide and also slightly soluble in water. Dilute solutions are pink, by coincidence very similar to the shady pink color of aqueous solutions of simple inorganic Mn^2+^ salts. UV–vis spectra reveal corresponding absorptions at 277–290 and 500–569 nm, respectively. Positive-mode ESI high-resolution mass spectra show signals of the most abundant monoisotopic peaks of the cations in excellent agreement with calculated values, thereby proving further the identity of these salts. IR spectra are rather unremarkable with dominating strong absorptions of the hexafluoridophosphate anions observed at 808–826 and 554–556 cm^–1^.

^1^H NMR spectra of **9**–**11** and **18**–**20** are overall rather simple (see the Experimental Section). The unsubstituted Cp ligands of **9** and **10** give rise to singlets at 4.73 or 4.67 ppm, whereas pseudotriplets for **11**, **18**, and **19** are detected at 4.67–5.20 ppm. In contrast, the protons of the unsubstituted Cht ligands of **9**, **11**, and **18**–**20** resonate at 6.63–6.84 ppm and are clearly deshielded in comparison to those of the Cp ligands, as expected for a formal cationic tropylium ligand. Quite similarly, ^13^C NMR spectra **9**–**11**, **18**, and **19** give Cp signals in the range of 77.3–86.4 and Cht signals in the range of 96.0–99.4 ppm, respectively. The ^1^H/^13^C NMR data for aminotromancenium **20** (δ(^1^H of Cp) = 4.32–4.43 ppm, δ (^1^H of Cht) = 6.63 ppm, δ (^13^C of Cp = 65.3, 73.3, 123.9 ppm), δ (^13^C of Cht = 97.5 ppm) are slightly different from those of the other tromancenium complexes. This is due to the amino donor substituent that imparts some fulvenoid iminium distortion, analogously to aminocobaltocenium salts.^8b^ In addition, ^1^H/^13^C signals for the methyl groups of **10** and **11** as well as signals for the carboxymethyl substituent of **18** and of the amino group of **20** are observed in the expected spectral regions.

**Figure 1:**
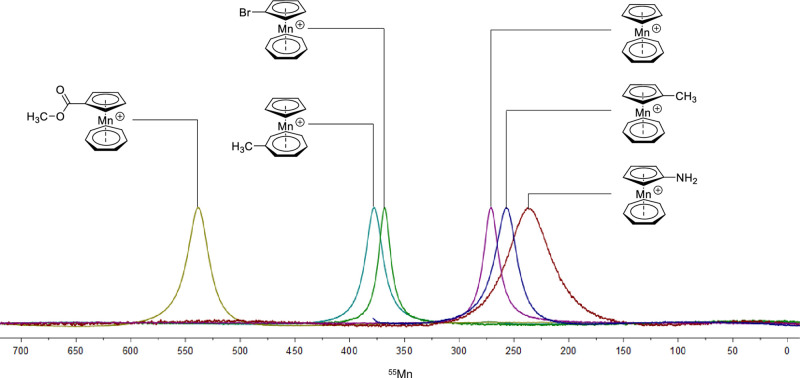
Overlay of ^55^Mn spectra of tromancenium salts in CD_3_CN solution, referenced versus saturated KMnO_4_/D_2_O solution: **9**, 271; **10**, 378; **11**, 257; **18**, 538; **19**, 368; **20**, 238 ppm.

^55^Mn-NMR spectroscopy provides a further uncommon option to gain direct information on the electronic properties of the metal center of tromancenium salts. In general, ^55^Mn-NMR is only rarely conducted despite its high sensitivity (natural abundance: 100%; receptivity relative to ^1^H: 0.179) because the high nuclear electric quadrupole moment (*Q* = 0.40 × 10^–28^ m^2^, *I* = 5/2) prevents the observation of signals for diamagnetic compounds of low symmetry. Diamagnetic manganese compounds are restricted to oxidation states −1, 0, +1, and +7, which, taken together with the predominance of high-spin complexes in oxidation states +2 and +3, limits the utility of ^55^Mn NMR spectroscopy in manganese coordination chemistry. However, Wrackmeyer^18^ obtained for a series of (arene)(cyclopentadienyl)Mn(I) complexes easily observable, fairly well resolved ^55^Mn NMR spectra with peak widths at half-height of only a few kHz, showing that such Mn(I) sandwich complexes are sufficiently “symmetric” with only small deviations from spherical charge distributions at the Mn nucleus. Gratifyingly, also tromancenium salts **9**–**11** and **18**–**20** gave very good ^55^Mn spectra (Figure 1, Experimental Section) with peak widths of approximately 1.5 kHz. Only aminotromancenium **20** displayed a broader signal. This agrees with a lower symmetry of the aminocyclopentadienyl ligand due to its partial iminium fulvene character. The ^55^Mn chemical shifts of **9**–**11** and **18**–**20** spread over a narrow range of 300 ppm, from 238 to 538 ppm, in line with their similar local ligand sphere at the manganese center. In general, ^55^Mn NMR is quite sensitive to electronic structure, as can be seen by comparison of the shift data of the tromancenium ions with those of (η^5^-C_5_H_5_)Mn(η^6^-C_7_H_8_) (**6**), δ(^55^Mn) = 1077 ppm,^18^ and cymantrene (η^5^-C_5_H_5_)Mn(CO)_3_ (**4**), δ(^55^Mn) = 2060 ppm.^19^ No linear correlation of ^55^Mn chemical shifts with Hammett substituent parameters was observed though,^20^ as it is rather common in transition metal NMR spectroscopy.

**Figure 2:**
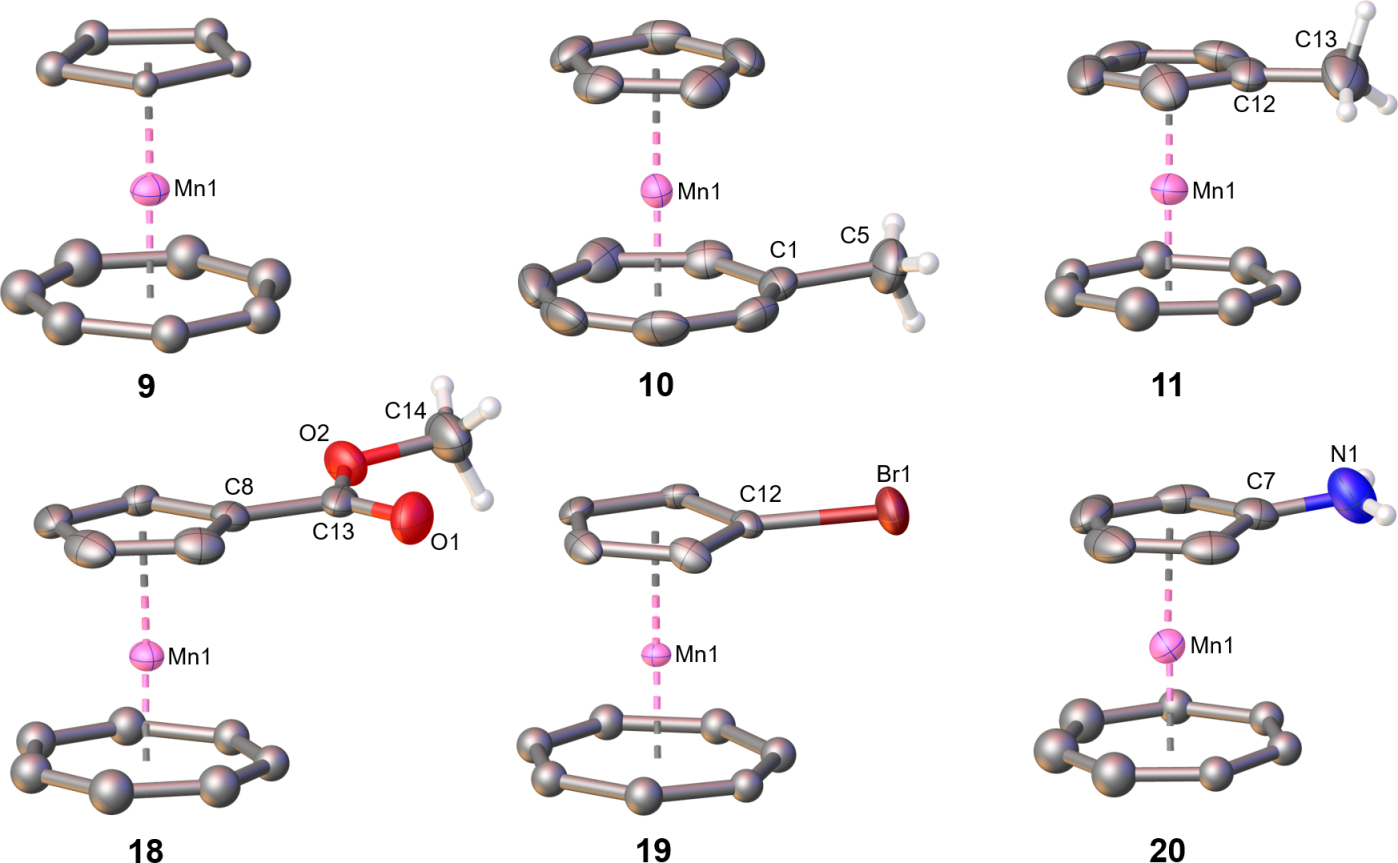
Molecular structures of tromancenium salts **9**, **10**, **11**, **18**, **19**, and **20**. Counteranions PF_6_^–^ and hydrogen atoms of the Cht and Cp ligands are omitted for clarity reasons. Selected bond lengths (Å): **9**: Mn–C_Cht_(avg) = 2.13, Mn–C_Cp_(avg) = 2.10; **10**: Mn–C_Cht_(avg) = 2.14, Mn–C_Cp_(avg) = 2.11, C1–C5 = 1.51(5); **11**: Mn–C_Cht_(avg) = 2.10, Mn–C_Cp_(avg) = 2.12, C12–C13 = 1.49(8); **18**: Mn–C_Cht_(avg) = 2.13, Mn–C_Cp_(avg) = 2.12, C8–C13 = 1.49(4), C13–O1 = 1.20(4), C13–O2 = 1.32(4); **19**: Mn–C_Cht_(avg) = 2.14, Mn–C_Cp_(avg) = 2.12, C12–Br1 = 1.88(3); **20**: Mn–C_Cht_(avg) = 1.98, Mn–C_Cp_(avg) = 2.10, Mn–C12 = 2.22(1), C7–N1 = 1.35(1).

The tromancenium salts crystallize readily, and single-crystal X-ray analyses are available for all six compounds (Figure 2, Supporting Information). Overall, the tromancenium cations display the typical sandwich metal structure of a central metal coordinated to parallel displaced carbocyclic ligands. The average manganese–carbon bond lengths are very similar for the Cp ligands (2.10–2.12 Å) and for the Cht ligands (2.10–2.14 Å). This drags the larger Cht ligands closer to the manganese centers with corresponding shorter Mn–Cht_centroid_ distances (approximately 1.3 Å) in comparison to those of the smaller Cp ligands (appr. 1.7 Å). Similar observations have been reported for other trometcenes.^21^ The substituents of the monosubstituted tromancenium salts, either in the 1-position (**10**) or the 8-position (**11**, **18**, **19**), have no appreciable effect on the structural metrics, so that they do not differ from parent **9**, as expected. However, 8-aminotromancenium (**20**) has a distorted structure with a slightly elongated Mn1–C7 bond (2.22(1) Å) and a shortened C7–N1 bond (1.35(1) Å), indicative of partial iminiumfulvene character, as it was already noted for the aminocobaltocenium ion.^8b^ This is also in line with ^1^H/^13^C/^55^Mn NMR solution data (vide supra).

**Figure 3:**
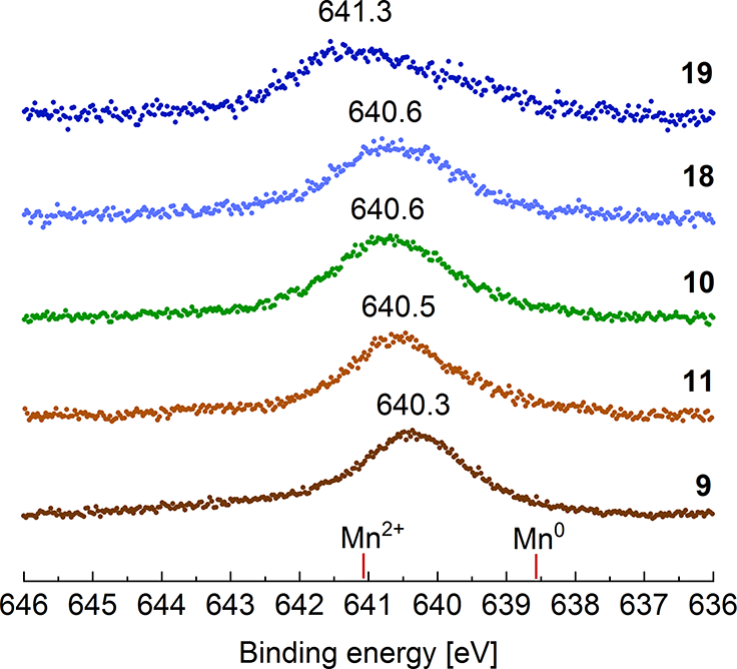
Stack of baseline-corrected XPS spectra of the Mn 2p_3/2_ region of tromancenium salts **9**–**11**, **18**, and **19**, including characteristic peak positions for Mn^0^ and Mn^2+^.

Photoelectron spectroscopy (XPS) was performed on all six tromancenium salts in order to gain more insight on their electronic structures. In principle, cycloheptatrienyl is a quite interesting ligand whose three closed-shell configurations C_7_H_7_^+^ (monocationic tropylium, 6 π-electrons, Hückel-aromatic), C_7_H_7_^–^ (monoanionic, 8 π-electrons, anti-Hückel-aromatic), or C_7_H_7_^3–^ (trianionic, 10 π-electrons, Hückel-aromatic) will impart corresponding different formal oxidation states to the coordinated metal center. In published XPS spectra of Mn(0) species, the 2p_3/2_ peak is located at 638.7 eV, whereas it is shifted to 641.4 eV in complexes of Mn(II).^22^ With 640.3 eV (**9**), 640.5 eV (**11**), 640.6 eV (**10**), 640.6 eV (**18**), and 641.3 eV (**19**), respectively, the binding energies of the present tromancenium salts fall in between these values (Figure 3, Supporting Information). Due to its electronegativity, the bromine substituent of **19** withdraws electron density from the metal center, resulting in a shift of the 2p_3/2_ signal toward the oxidation state +2. For the amino-substituted complex **20**, the XPS spectrum of the 2p region of manganese is of poor quality, most likely due to decomposition during the measurement, preventing in this case to determine the oxidation state of the metal center. Overall, our XPS spectra clearly indicate that the manganese center of tromancenium salts is best described by the oxidation state +1, thereby assigning the cycloheptatrienyl ligand a cationic tropylium structure C_7_H_7_^+^. In contrast, early transition metal (Cht)M(Cp) sandwich complexes, M = Ti, Zr, Hf,^3^ contain cycloheptatrienyl ligands that are more adequately described as trianionic C_7_H_7_^3–^ ligands, thereby attributing an oxidation state of +4 to Ti, Zr and Hf in these compounds.^3^

### Electrochemistry

Before turning to the redox properties of the cationic tromancenium complexes, we will briefly summarize those of the titanium, vanadium, and chromium congeners troticene, trovacene, and trochrocene (Table 2). Their valence electron counts of 16 (Ti), 17 (V), and 18 (Cr) are reflected according to differences in electronic configurations and orbital occupancies ranging from e_2_^4^a_1_^0^ (Ti) to e_2_^4^a_1_^1^ (V) and e_2_^4^a_1_^2^ (Cr). The Ti congener undergoes one-electron reduction at −2.70 V. While being sufficiently stable in THF at −20 °C to be characterized by EPR and UV/vis spectroscopy, the resulting anion is highly reactive at room temperature and gives rise to a chemical follow product. The latter oxidizes at a less cathodic potential of −2.47 V to regenerate the starting material. The authors assumed a reversible hapticity change of the cycloheptatrienyl ligand to account for the overall square-scheme (or box) mechanism. Oxidation of this complex is chemically irreversible under all conditions and leads to rapid electrode passivation.^23^ The 17 valence-electron trovacene can be oxidized and reduced reversibly at *E*_1/2_ = −0.23 V or *E*_1/2_ = −3.04 V to generate a persistent cation and anion, respectively.^24^ Further oxidation at ca. + 0.61 V occurs as two overlapping anodic peaks and results in rapid degradation to unknown products. Trochrocene, which shares its 18 VE count with the present tromancenium ions, reduces at ca. −3.38 V to generate, after proton atom abstraction from the NBu_4_^+^ cation of the supporting electrolyte, the neutral cycloheptatriene complex (η^6^-C_7_H_8_)(η^5^-C_5_H_5_)Cr.^24b^ Quite interestingly, this latter complex, on exposure to a potassium mirror, (re)generates the trochrocene anion, which is also directly formed on reduction of trochrocene in the complete absence of a proton source.^25^ Its associated radical cation, which is formed at a half-wave potential of −1.10 V, is stable under even less stringent conditions.^25,26^ Like for trovacene, further oxidation occurs as a chemically irreversible two-electron process at a much more positive peak potential of +1.10 V.^24b^

**Table 2:** Redox Potentials of the Tromancenium Ions of This Study and the Parent Troticene, Trovacene and Trochrocene^a^,^b^

compd^b^	*E*_ox_	*E*_red_	solvent	further redox processes
[(Cht)Mn(Cp)]^+^ (**9**)	+0.70^c^	–1.61^d^	DMF	associated reduced form gives rise to a chemical follow product, which oxidizes at –0.52 V
[(^Me^Cht)Mn(Cp)]^+^ (**10**)	+0.69^c^	–1.62^d^	DMF	associated reduced form gives rise to a chemical follow product, which oxidizes at –0.55 V
[(Cht)Mn(Cp^Me^)]^+^ (**11**)	+0.68^c^	–1.66^d^	DMF	associated reduced form gives rise to a chemical follow product, which oxidizes at –0.54 V
[(Cht)Mn(Cp^COOMe^)]^+^ (**18**)	+0.88^c^	–1.46^d^	CH_3_CN	associated reduced form gives a chemical follow product, which oxidizes at –0.44 V; further irreversible reduction peak at –2.50 V
[(Cht)Mn(Cp^Br^)]^+^ (**19**)	+0.93^d^	–1.49^e^	DMF	associated reduced form gives rise to a chemical follow product, which oxidizes at –0.31 V
[(Cht)Mn(Cp^NH2^)]^+^ (**20**)	+0.40	–1.33	DMF	additional oxidation wave at +0.65 V and reduction peaks at –1.84 V and –3.16 V
[(Cht)Ti(Cp)]	–0.01^d^	–2.70^e^	THF	associated reduced form gives rise to a chemical follow product, which oxidizes at –2.47 V and regenerates the starting complex^23b^
[(Cht)Ti(Cp*)]	–0.18^d^	–2.78^e^	THF	associated reduced form gives rise to a chemical follow product, which oxidizes at –2.56 V^23b^
[(Cht)V(Cp)]	–0.23	–3.04	DME	further, chemically irreversible 2e^–^ oxidation at ca. +0.61 V^23b^
[(Cht)Cr(Cp)]	–1.10	–3.38^e^	DME	associated anion rapidly forms (η^6^-C_7_H_8_)CrCp by proton abstraction; further oxidation as a chemically irreversible process at +1.10 V^23b^

a Potentials are given in volts and are calibrated against the Cp_2_Fe^0/+^ redox couple.

b Cht = η^7^-cycloheptatrienyl, Cp = η^5^-cyclopentadienyl.

c Half-wave potential of a chemically only partially reversible process.

d Peak potential of a chemically irreversible redox process.

e Potential calibration against the Cp_2_Fe^0/+^ redox couple.^24c^

**Figure 4:**
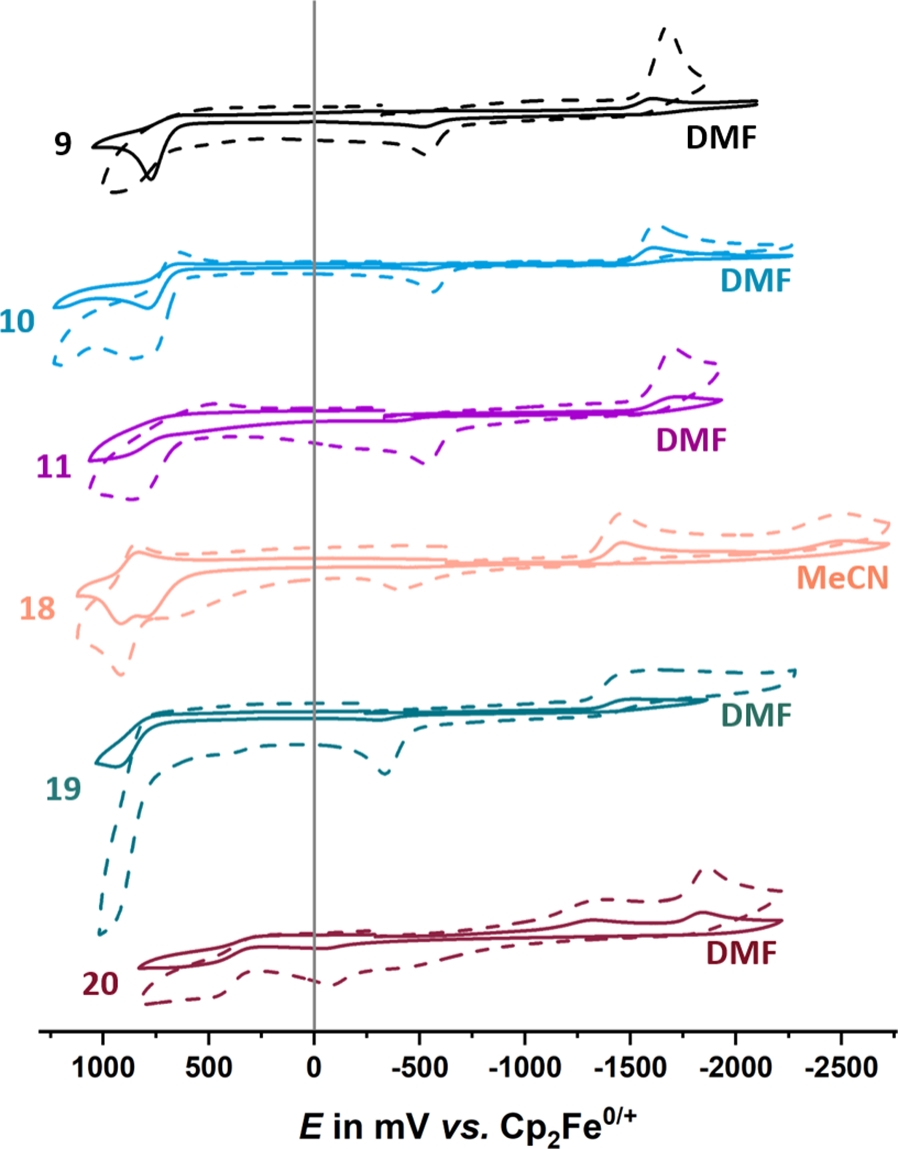
Cyclic voltammograms of the tromancenium ions **9**–**11** and **18**–**20** in DMF or MeCN (0.1 M NBu_4_^+^ PF_6_^–^) on a glassy carbon working electrode at sweep rates *v* of 0.1 (solid line) and 0.6 V s^–1^ (dotted line).

The electrochemistry on the present tromancenium ions was assessed in DMF and CH_3_CN as solvents, using 0.1 M NBu_4_^+^ PF_6_^–^ as the supporting electrolyte. CV responses in these solvents are in most cases rather similar (see Table 2 and Figure 4), with some exceptions (see below). The choice of the working electrode proved to be critical. Platinum led to severe electrode passivation even upon a single scan, with often erratic shifts of peak potentials. Better results were obtained on glassy carbon (for a comparison of **10** at different scan rates, see Figure S44), but even then, severe electrode passivation was noted. This required wiping of the electrode surface after every scan and frequent repolishing with diamond pastes after only a few scans in order to obtain reproducible results.

Considering that trochrocene and the tromancenium ions share the same valence electron count of 18, but differ by their outer charge, one would expect an anodic shift of all redox processes for the manganese congener, but overall similar redox properties. This proved to be the case, as the half-wave/peak potentials for tromancenium oxidation/reduction exhibit a sizable anodic shift of ca. 1.8 V compared to those of neutral trochrocene (see Table 2).

**Figure 5:**
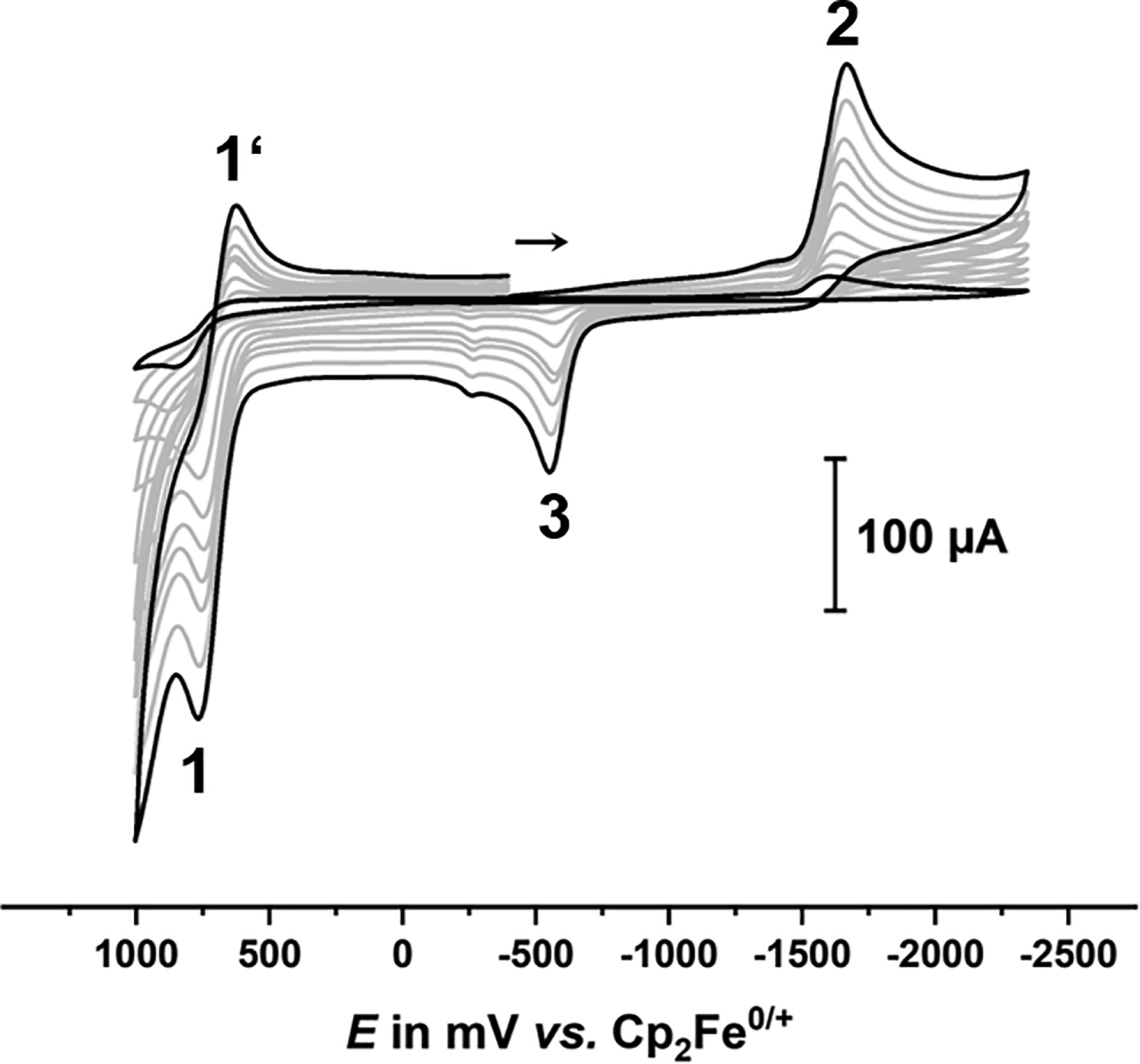
Cyclic voltammograms of the parent tromancenium ion **9** (4 mM) in DMF/0.1 M NBu_4_^+^ PF_6_^–^ on a glassy carbon working electrode at rt and at sweep rates *v* of 0.025 to 2.0 V s^–1^.

Figure 5 and Figure S45 illustrate the typical appearance of the cyclic voltammograms studied in this work as exemplified with the parent tromancenium ion **9**. All tromancenium ions undergo an oxidation, denoted as peaks 1/1′ (cf. *E*_ox_ in Table 2) in Figure 4, and an irreversible reduction (cf. *E*_red_ in Table 2), marked as peak 2 in Figure 5. Both processes involve the transfer of a single electron as associated from peak widths and the similar peak currents. Anodic oxidation is (close to) irreversible at low seep rates but becomes chemically partially reversible at faster sweep rates. Scan rates of 0.4 V s^–1^ or more are required in order to observe the associated cathodic return peak, even for the most electron-rich amino-substituted congener (see Figure 4). No additional peak/wave ascribable to a follow product was observed under conditions, where the oxidation was largely irreversible.

Reduction gives rise to an electroactive follow product, which oxidizes ca. 1.1 V anodic of the original reduction peak (denoted as peak 3 in Figure 5) and provides no associated cathodic return peak on scan reversal (see Figure S46). In agreement with its formation during the reductive scan, peak 3 is only observed after prior reduction as shown in Figure S46. Attempts to investigate whether the follow product reverts to the starting tromancenium compound on reoxidation were thwarted by excessive electrode fouling as it is exemplarily shown in Figure S47. Chemical irreversibility of the initial reduction also prevails on lowering *T* to −40 °C with even more severe problems of electrode passivation. A representative set of scans at variable *T* is shown as Figure S48.

**Figure 6:**
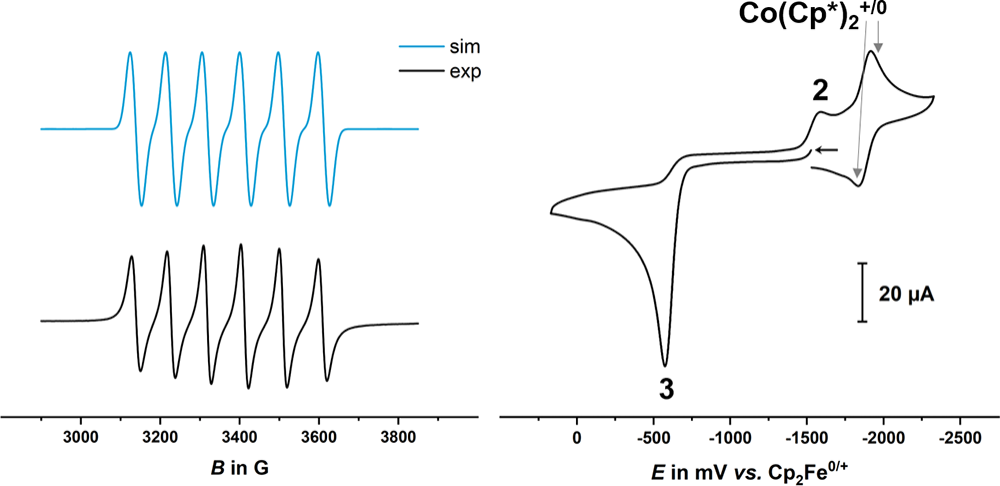
(Left) EPR spectrum (black) and simulation (blue) of chemically reduced **9** prepared by reaction with 1.2 equiv of decamethylcobaltocene at 20 °C in DMF solution. (Right) CV of a chemically reduced sample of **9** in DMF/0.1 M NBu_4_^+^ PF_6_^–^ at a glassy carbon working electrode and at a sweep rate *v* of 0.2 V s^–1^.

In order to possibly gain further insights on the follow product formed after reduction, complex **9** was chemically reduced with 1.2 equiv of decamethylcobaltocene and *T*-dependent EPR spectra were recorded in DMF as shown in Figure S49. As presented on the left of Figure 6, the EPR spectrum at 20 °C could be simulated with hyperfine couplings to one ^55^Mn nucleus (*A* = 265 G).

When the chemically reduced sample was analyzed by cyclic voltammetry, the wave assignable to the electrogenerated follow product now appeared as a chemically irreversible forward wave (peak 3 in Figure 6, right), and the original reduction peak (peak 2 in Figure 6, right) was observed again. This peak was absent if the scan was clipped before scanning through peak 3. This experiment suffered, however, from severely absorptive behavior and particularly problematic electrode passivation. Nevertheless, these results support that the **9**/**9**^**–**^ redox system and the electroactive product, which is generated from **9**^**–**^, constitute an overall (at least partially) reversible redox system, which obeys an overall “square scheme” mechanism.^27^

In order to also probe for a higher order reaction (e.g., a dimerization), we monitored the ratio of the peak currents associated with the chemical follow peak 3 (*i*_pf,a3_) and of the initial cathodic reduction peak 2 (*i*_p,c2_) at three different analyte concentrations and over a range of different sweep rates (for results on complex **9**, see Figures S50–S51). At identical sweep rates, the ratio *i*_pf,a3_/*i*_p,c2_ is, within the error margins, independent of the analyte concentration *c.* This argues against such a possibility, leaving hydride abstraction of the ensuing neutral radical as a plausible scenario.

**Figure 7:**
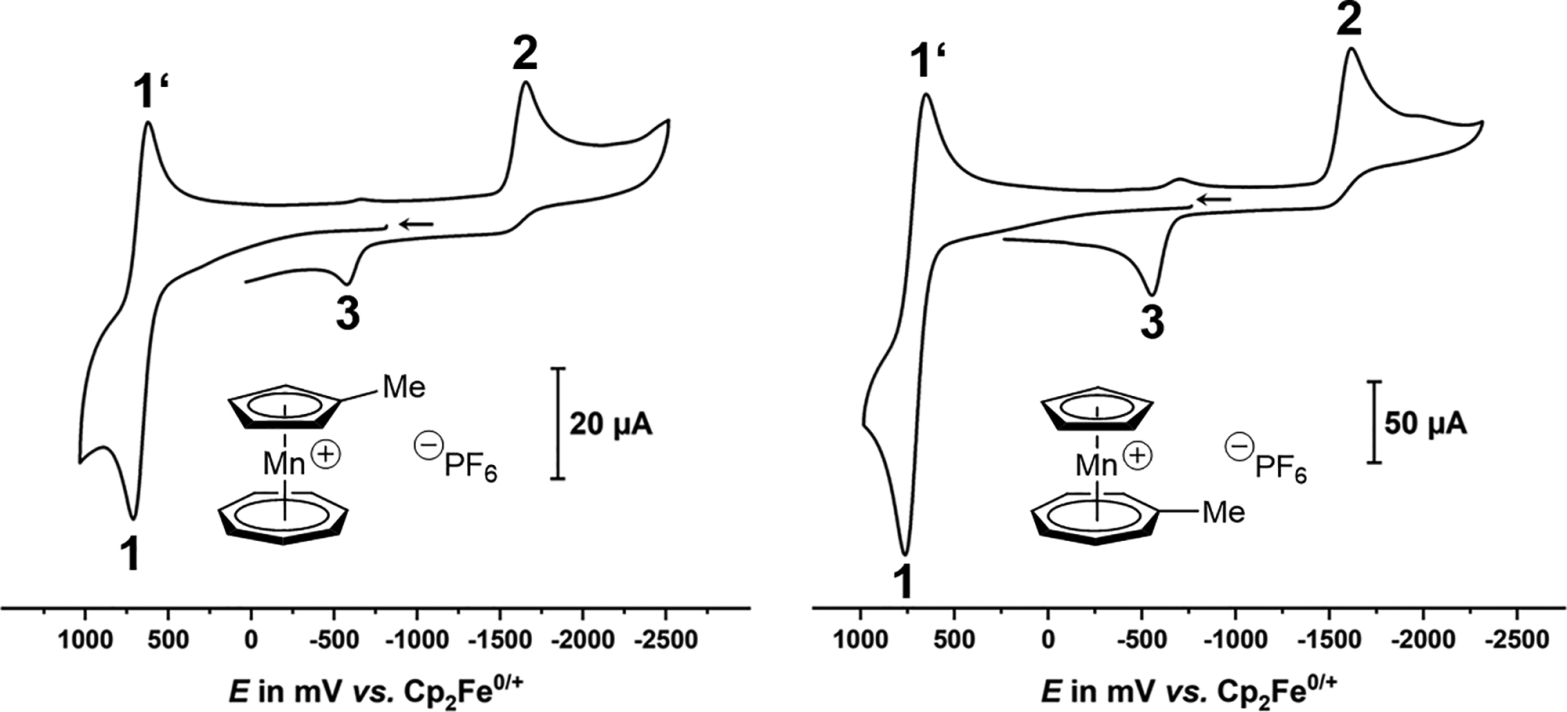
Cyclic voltammograms of the isomeric methyltromancenium ions **11** (left) and **10** (right) in DMF/0.1 M NBu_4_^+^ PF_6_^–^ on a glassy carbon working electrode at rt and at *v* = 0.6 V s^–1^.

Methyl substitution at either the cycloheptatrienylium (**10**, right of Figure 7) or the cyclopentadienide (**11**, left of Figure 7) ligand has the expected effect of shifting the reduction, and to a minor extent, the oxidation potential to more cathodic values (cf. Table 2). For the reduction of compound **11**, the shift induced by methyl substitution at the cyclopentadienide ligand comes close to that of −0.05 to −0.06 V observed in methylferrocene as compared to ferrocene.^28^ It is notably larger compared to that of substitution at the seven-membered ring in **10** (Table 2). In both complexes, **10** and **11**, methyl substitution slightly stabilizes the associated radical cations as shown by the enhanced currents of the cathodic return peak 1′ when compared to the parent tromancenium ion (compare Figures 5 and 7).

Further modification by replacing the methyl substituent of compound **11** with an electron acceptor or a stronger donor has concomitant consequences on the redox potentials. 8-Carboxymethyltromancenium **18** thus exhibits anodic shifts for both processes of 0.18 or 0.15 V, respectively. Scanning past the primary reduction peak reveals another irreversible reduction at ca. −2.48 V (−2.50 V in MeCN), which probably corresponds to the further reduction of the ester functionality within the electrogenerated follow product (cf. Figure S56), which reveals itself by an anodic follow peak at −0.44 V (Figure 4 and top of Figure S57). Oxidation of 8-bromotromancenium **19** constitutes a chemically irreversible process under all applied conditions (cf. Table 2 and Figures S58–59). Reduction is likewise ill-behaved and gives rise to several small cathodic peaks on the reverse scan (see right side of Figure S59). The oxidation and reduction potentials of the 8-aminotromancenium ion, **20**, the most electron-rich congener of this series, are by ca. 0.3 V more cathodic than that of the parent tromancenium ion. We also note an additional oxidation wave at +0.65 V as well as an additional reduction peak at −3.16 V (cf. Figure S60), close to the cathodic discharge limit of the DMF electrolyte. Representative CV scans are shown as Figures S60 and S61.

### Quantum Chemical Calculations

To further characterize the chemical nature and the bonding of tromancenium **9**, quantum chemical studies were performed to gain an understanding of its (electronic) structure. Geometry optimizations with various density functionals revealed that the η^5^ coordination of the cyclopentadienyl and the η^7^ coordination of the cycloheptatrienyl ligands—as evident in the crystal structure—remains intact in the gas phase and in implicit DMSO. C–C double bonds are completely delocalized over the 5-membered ring, while there are small bond-length alternations in the 7-membered ring. The distance of the cyclopentadienyl ring to Mn is at around 1.7 Å and thereby around 0.4 Å longer than that of the cycloheptatrienyl ring. Overall, the optimized structures are similar to the crystal structure and show little dependence on the employed density functional (compare also Table S1). Analysis of Hirshfeld partial charges indicate that the positive charge of the cationic species **9** is allocated at the Mn center (see Table S3).

**Figure 8:**
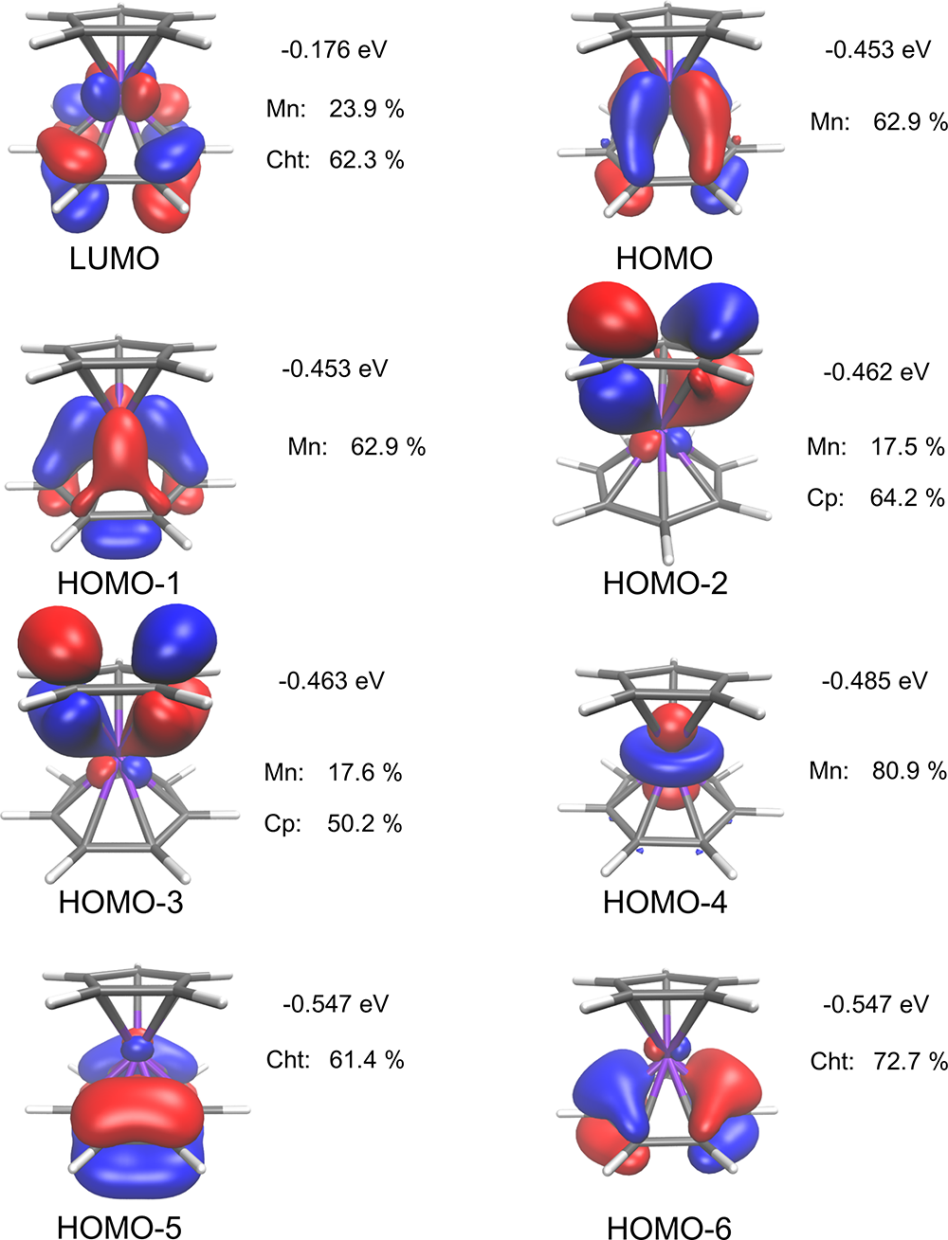
Frontier molecular orbitals calculated with PBE0-D3/def2-TZVPP. An isosurface of 0.05 arbitrary units was used for visualization.

To shed light on the electronic structure of **9**, frontier molecular orbitals were investigated. As visible from Figure 8, both the highest occupied molecular orbital (HOMO) as well as the lowest unoccupied molecular orbital (LUMO) are centered at the metal or at the metal and the cycloheptatrienyl ring, but not on the cyclopentadienyl ring. This trend is consistent for various density functionals (compare Figures S39 and S40). In fact, the first molecular orbital that has notable contributions from the cyclopentadienyl ligand is HOMO–2. These findings suggest that redox or chemical reactions take place either at the Mn center or at the 7-membered ring, but not at the Cp ligand. This is in line with our experimental electrochemical studies (vide supra).

Calculated Brønstedt acidities in dimethyl sulfoxide (modeled as implicit solvent with ε = 47.2) are very low with the cyloheptatrienyl ligand being slightly more acidic than the cyclopentadienyl ligand [p*K*_a_(Cht) = 42(±1), p*K*_a_ (Cp) = 44(±2)]. For comparison, the calculated p*K*_a_ of cobaltocenium hexafluoridophosphate is 38.5(±2).^8d^

When we reduced **9** in an in silico experiment to the neutral 19-electron “tromancene” species **9**^**–**^, we observed a distinct behavior. While the cyclopentadienyl ligand kept its η^5^ coordination, the hapticity of the cyloheptatrienyl heavily depends on the employed density functional–ranging from η^4^ to η^7^ (Table S2). In addition, the energy splitting between the doublet and the quartet states also showed a dependence on the density functional with PBE0-D3 and ωB97x-d, predicting the quartet state to be more stable by Δ*E*_d-q_ = ca. 50 kJ/mol and Δ*G*_d-q_^298 K^ = ca. 60 kJ/mol, whereas BP86-D3 favors the doublet state (compare Table S2), which is in line with previous findings.^29^ A recent combined experimental and computational benchmark study on a series of related metallocenes found the ωB97x-d density functional to yield accurate results for the spin-state energetics of a related [MnCp_2_] compound.^30^ Hence, we assume these calculations to be more accurate and surmise the quartet state to be the more stable one here. Regardless of the differences in the predicted spin-state splitting and the different coordination pattern of the cycloheptatrienyl ligand, the spin density is in all instances located at the metal center (Figures S65–S67). Analysis of the Hirshfeld partial charges exemplary calculated with the PBE0 density functional revealed a positively charged metal center (Table S3).

## Summary

A custom-made high-power LED photoreactor was proven to be highly advantageous in comparison to standard photochemical methodology for the convenient and rapid synthesis of six tromancenium salts. Substitution of the three carbonyl ligands of cymantrenes under blue light irradiation with cycloheptatrienes followed by oxidation/hydride removal with tritylium hexafluoridophosphate afforded the parent tromancenium hexafluoridophosphate [η^7^-C_7_H_7_)Mn(η^5^-C_5_H_5_)]PF_6_ and five monosubstituted derivatives containing methyl, methoxycarbonyl, bromo, and amino substituents in 97–10% yield, dependent on the photochemical stability of the substituents.

Tromancenium salts are 18-valence-electron, air-stable, polar compounds of red color, soluble in common polar organic solvents and also in water. Full spectroscopic characterization by HRMS, IR, UV–vis, and ^1^H/^13^C NMR, and notably also by ^55^Mn-NMR, is reported. Single-crystal analyses show typical sandwich metal complex structures with parallel displaced cycloheptatrienyl and cyclopentadienyl rings and more or less identical manganese–carbon bond lengths for the Mn–cycloheptatrienyl and Mn–cyclopentadienyl carbon bonds. The electronic structures of tromancenium salts are best described by a manganese center of formal oxidation state +1 coordinated to a 6π-electron cyclopentadienide and a 6π-electron tropylium ligand, as shown by manganese photoelectron spectroscopy.

Detailed quantum chemical calculations of the parent tromancenium hexafluoridophosphate indicate that metal–ligand bonding is distinctly weaker for the cycloheptatrienyl ligand in comparison to that of the cyclopentadienyl ligand. Both the HOMO (highest occupied molecular orbital) and the LUMO (lowest unoccupied molecular orbital) are metal and cycloheptatrienyl-ligand centered, indicating that redox processes or electrophilic and nucleophilic chemical reactions will occur either metal-based or at the seven-membered ring, but not on the cyclopentadienyl ligand.

Electrochemically, tromancenium salts can be oxidized to the dication and reduced to the neutral species at rather low potentials, although not reversibly. The half-wave/peak potentials respond to the inductive effects of the substituents at the cyclopentadienyl or the cycloheptatrienyl ligands. The product generated by reduction of the parent complex was studied in more detail. Chemical reduction yielded an EPR active species with a resolved hyperfine splitting to one ^55^Mn nucleus. The latter species regenerates at least partially the parent complex on reoxidation, thus establishing an overall square scheme mechanism.

With this work we show that the long neglected and forgotten tromancenium complexes are readily available by photochemical synthesis with modern high-power LED light sources. In a broader context, LED-based photochemistry will most likely replace standard photochemical methodology and will become a fertile area of organometallic, inorganic and organic synthetic chemistry.

## Experimental Section

### General Procedures

Standard methods and procedures of organic/organometallic synthesis, spectroscopic characterization and single-crystal structure XRD analysis were performed. ^1^H, ^13^C, and ^55^Mn NMR spectra were recorded at 25 °C on a Bruker Avance DPX 300 NMR spectrometer, and signals were referenced internally against ^1^H/^13^C residual solvent peaks or externally (^55^Mn) against a saturated KMnO_4_/D_2_O solution. Mass spectrometric data were measured on a Thermo Finnigan Q Exactive Orbitrap spectrometer, IR spectra were recorded on a Bruker ALPHA IR spectrometer, UV–vis spectra were measured on a PerkinElmer Lambda XLS+ spectrometer, single-crystal X-ray diffraction data were collected on a Bruker D8 Quest diffractometer with graphite-monochromated Mo–K_α_ radiation (λ = 0.71073 Å), and structures were solved by direct methods.

Cyclic voltammetry was performed as described recently.^8d^ T-dependent (thermostat with temperature controller HO3 using liquid nitrogen) electron paramagnetic resonance (EPR) spectroscopy was performed on a X-band spectrometer MiniScope MS5000 by Magnettech GmbH in combination with the program ESR Studio 1.63.0. The EPR sample was prepared by chemical reduction of complex **9** with 1.2 equiv of decamethylcobaltocene in DMF inside a nitrogen glovebox.

For XPS measurements, the powders were freshly grounded before the measurement under nitrogen atmosphere in a mortar and applied as thin layer on an adhesive copper foil which was then transferred into the vacuum system without air exposure. Measurements were performed on a multichamber ultrahigh vacuum system at a pressure of 5 × 10^–10^ mbar using a Phoibos 100 hemispherical analyzer (Specs). As the excitation source, an Al–K_α_ anode was used (h·ν = 1486.6 eV, probing depth ∼10 nm). Due to charging effects during measurements caused by the low conductivity of the powder samples, the binding energy scale as measured by XPS was shifted by a few electron volts for the different samples; to account for this, the binding energies were corrected such that adventitious carbon is positioned at 284.8 eV. Upon extended X-ray exposure, some samples showed oxidation and decomposition effects. Therefore, data collect times were kept short (around 20 min) to ensure that only the pristine material is probed. To achieve sufficient count rates, a rather high pass energy of 40 eV was used.

### Starting Materials

Chemicals were obtained commercially and used as received. *Note*: If cymantrene, CpMn(CO)_3_, is obtained commercially, its quality has to be critically tested by TLC, PXRD and ^55^Mn-NMR. Recently ordered batches from various vendors with a specified purity of 98% had an actual cymantrene content of only 37% in admixture with Mn_2_(CO)_10_, indicating that these chemicals originated from the same primary source. Mn_2_(CO)_10_ is the starting material in cymantrene synthesis and has similar physical properties (appearance, color, sublimation point), it can only be separated from cymantrene by chromatography on alumina with pentane as eluent. Hence, we recommend synthesis of cymantrene freshly.^19^ 7-Methylcyclohepta-1,3,5-triene,^31^ methoxycarbonylcymantrene,^12^ bromocymantrene,^13^ and aminocymantrene^15^ were prepared according to literature.

### Photochemical Syntheses

#### Caution

**Figure 9:**
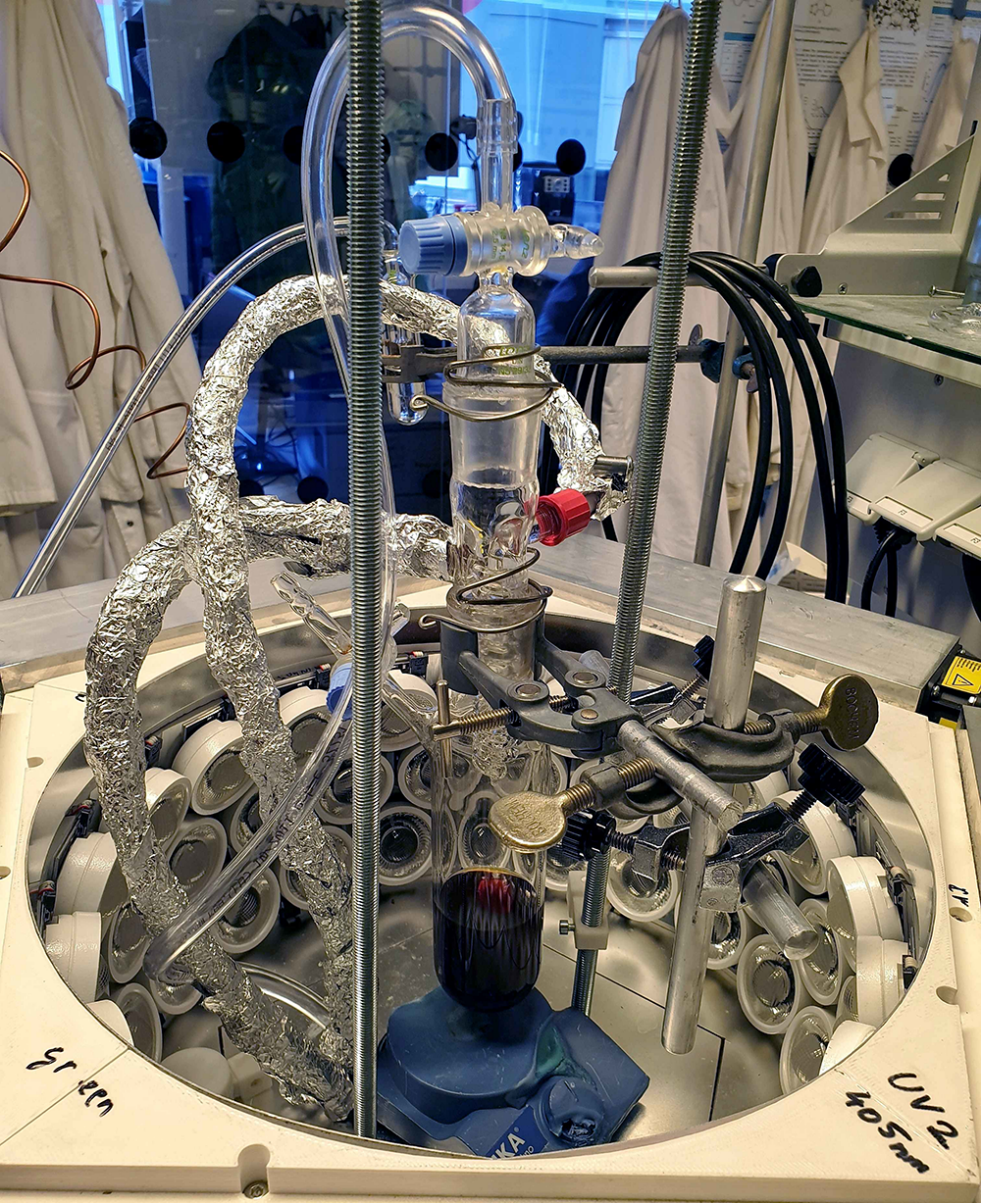
“Solar Light Lab Luminaire” photochemical apparatus with removed cover.

In all photochemical syntheses with high-power light sources, appropriate eye protection or only an apparatus with a completely covered setup during irradiation is mandatory. Photochemical syntheses were performed in Schlenk vessels equipped with a reflux condenser under strict exclusion of O_2_ in dry, purified, freeze–thawed, Ar-saturated solvents and starting materials using a custom-made high-power LED-reactor “Solar Light Lab Luminaire” (Figure 9) developed in collaboration with the Austrian company Bartenbach.^10^ This apparatus allows irradiation of chemical solutions on a 5–100 mL scale in a controlled manner with monochromatic light, ranging from 360 nm with 260 W electrical power up to 535 nm with 370 W electrical power or with polychromatic cool white, respectively. Thereby, selective photochemical activation with either UV, blue, green, or white light is possible with significantly reduced irradiation times of less than 30 min compared to 8 h or more commonly used in a traditional falling-film photoreactor with medium-pressure mercury lamps. The LED photoreactor is also equipped with a photodiode that allows measuring of the photon flux in the center of the apparatus corresponding to the volume of the solution, thereby allowing calculation of the quantum yield.

### DFT Calculations

A starting structure of **9** was derived from the existing crystal structure and subjected to quantum chemical studies with dispersion corrected density functional theory calculation in the gas phase and in DMSO modeled as implicit solvent with ε = 47.2. **9** was optimized with the hybrid PBE0 density functional,^32^ the pure BP86 density functional,^33^ and the range-separated hybrid density functional ωB97x-d^34^ in combination with the triple-ζ def2-TZVPP basis set.^35^ For speed-up, the resolution-of-identity technique was used.^36^ Empirical dispersion corrections with Becke–Johnson damping were added in the case of PBE0 and BP86.^37^ Bulk solvent effects of DMSO were accounted for with the Conductor-Like Screening Model (COSMO),^38^ where the solvent was described with a dielectric constant ε = 47.2. **9** was considered in its closed-shell state only (i) because a singlet state is consistent with experimental observations and (ii) because the energies of the triplet states were found to be between 70 to 115 kJ/mol higher in energy than the singlet state. Frontier molecular orbitals were calculated with PBE0 and BP86 and–for sake of comparison^34^–also with M05-2X^39^/def2-TZVPP.^35^ All frontier molecular orbitals were generated from single-point calculations on the PBE0-optimized structures and calculated with Gaussian 16 as it provided the contribution of the individual atoms to the respective orbitals.^40^ p*K*_a_ values were determined by a thermodynamic cycle as detailed in the Supporting Information. To obtain Gibbs free energies (for the calculation of p*K*_a_ values), zero-point energy and thermal corrections calculated with the rigid-rotor/quasi-harmonic oscillator were added to the electronic energy. To increase the accuracy calculated frequencies were scaled with a factor of 0.9821 (PBE0), of 1.0181 (BP86), and of 0.9779 (ωB97x-d)^41^ and frequencies below 50 cm^–1^ were set to 50 cm^–1^.^42^ For the investigation of the reduced 19-electron species **9**^**–**^, spin-unrestricted calculations were carried out both in the doublet and in the quartet state. Partial charges were calculated according to Hirshfeld^43^ as the difference between the molecular and the atomistic charge density as implemented in Gaussian 16.^40^ Unless otherwise noted, all calculations were performed with the quantum chemical program package Turbomole.^44^ Structures were visualized with PyMol^45^ and VMD.^46^

### η^7^-Cycloheptatrienyl-η^5^-cyclopentadienylmanganese (Tromancenium) Hexafluoridophosphate (**9**)

A Schlenk flask, equipped with a reflux condenser and a bubbler, was charged with 0.200 g of cymantrene (0.980 mmol, 1.00 equiv), 162 μL of 1,3,5-cycloheptatriene (1.470 mmol, 1.50 equiv), and 20 mL of dry heptane. The apparatus was then irradiated with blue light (450 nm) and 370 W for 15 min until no further carbon monoxide evolution was observed. *Note*: A successful reaction is indicated by a color change from yellow to red. Heptane was removed in vacuo. The intermediate highly air-sensitive -complex was dissolved in dichloromethane (abs) and cooled to 0 °C. Tritylium hexafluoridophosphate (0.570 g, 1.470 mmol, 1.50 equiv) was added in one portion, and the solution was stirred for additional 30 min under exclusion of light. After precipitation with diethyl ether, the air stable crude product was filtered off and washed three times with 10 mL portions of diethyl ether. The crude product was dissolved with acetonitrile from the folded filter. The solvent was removed in vacuo and the solid residue was purified by a short column chromatography on neutral alumina with acetonitrile–diethyl ether (3:7). After yellow remains of nonpolar trityl are removed completely, tromancenium hexafluoridophosphate is eluted with acetonitrile, affording **9** as red crystals in 73.9% yield (0.258 g, 0.724 mmol). An overall quantum yield of 1.5% was calculated. Compound **9** is air-stable and soluble in acetone, acetonitrile, and dimethyl sulfoxide. Mp: 246 °C. ^1^H NMR (300.1 MHz, CD_3_CN, ppm): δ = 4.73 (s, 5H, C8–12 of Cp), 6.84 (s, 7H, C1–7 of Cht). ^13^C NMR (75.5 MHz, CD_3_CN, ppm): δ = 78.3 (C8–12 of Cp), 97.5 (C1–7 of Cht). ^55^Mn-NMR (74.4 MHz, CD_3_CN, ppm): δ = 271.0. IR (ATR, cm^–1^): 3123 (ν_C–H_), 3079 (ν_C–H_), 1448 (ν_C=C_), 1428 (ν_C=C_), 1016 (δ_C–H_), 810 (ν_P–F_), 554 (δ_P–F_), 445 (ν_metal-ring_). HRMS (ESI pos, *m*/*z*): 211.0309 ([M – PF_6_]^+^), calcd for C_12_H_12_Mn: 211.0314. UV/vis (CH_3_CN, [nm]): λ_max1_ = 278, λ _max2_ = 544. Single crystals of **9** were obtained via diffusion crystallization in acetonitrile out of diethyl ether at room temperature. Single-crystal analysis (Figure 2, Supporting Information).

### η^7^-Methylcycloheptatrienyl-η^5^-cyclopentadienylmanganese (1-Methyltromancenium) Hexafluoridophosphate (**10**)

Analogous to the synthesis of **9** described above, **10** was prepared starting from 0.275 g of cymantrene (1.350 mmol, 1.00 equiv), 210 μL of 7-methylcyclohepta-1,3,5-triene (1.691 mmol, 1.25 equiv), and 10 mL of heptane (abs). Irradiation with blue light (450 nm) and 370 W for 15 min and subsequent reaction with tritylium hexafluoridophosphate (0.655 g, 1.686 mmol, 1.25 equiv) afforded 1-methyltromancenium hexafluoridophosphate (**10**) as red crystals in 25.0% yield (0.125 g, 0.338 mmol) in a quantum yield of 5.3%. Mp: 206 °C dec. ^1^H NMR (300.1 MHz, CD_3_CN, ppm): δ = 2.80 (s, 3H, CH_3_), 4.67 (s, 5H, C8–12 of Cp), 6.70 (m, 6H, C2–C7 of Cht). ^13^C NMR (75.5 MHz, CD_3_CN, ppm): δ = 26.4 (CH_3_), 78.7 (C8–12 of Cp), 96.0 (C2/C7 of Cht), 97.0 (C3/C6 of Cht), 97.8 (C4/C5 of Cht), 112.8 (ipso-carbon of Cht). ^55^Mn-NMR (74.4 MHz, CD_3_CN, ppm): δ = 378.2. IR (ATR, cm^–1^): 3117 (ν_C–H_), 1459, 1427, 1384 (ν_C=C_), 1030, 1015 (δ_C–H_), 812 (ν_P–F_), 554 (δ_P–F_), 448 (ν_metal-ring_). MS (ESI pos, *m*/*z*): 225.0467 ([M – PF_6_]^+^), calcd for C_13_H_14_Mn: 225.0471. UV/vis (CH_3_CN, nm): λ_max1_ = 278, λ_max2_ = 547. Single crystals of **10** were obtained from acetonitrile and diethyl ether at room temperature. Single-crystal analysis (Figure 2, Supporting Information).

### η^7^-Cycloheptatrienyl-η^5^-methylcyclopentadienylmanganese (8-Methyltromancenium) Hexafluoridophosphate (**11**)

Analogous to the synthesis of **9** described above, **11** was prepared starting from 0.385 mL of methylcymantrene (2.45 mmol, 1.00 equiv), 404 μL of 1,3,5-cycloheptatriene (3.86 mmol, 1.50 equiv), and 50 mL of dry heptane. Irradiation with blue light (450 nm) and 370 W for 45 min and subsequent reaction with tritylium hexafluoridophosphate (1.048 g, 2.70 mmol, 1.10 equiv) afforded 8-methyltromancenium hexafluoridophosphate (**11**) as red crystals in 97.4% yield (0.882 g, 2.38 mmol) in a calculated quantum yield of 3.2%. Mp: 256 °C dec. ^1^H NMR (300.1 MHz, CD_3_CN, ppm): δ *=* 1.91 (s, 3H, CH_3_), 4.67 (s, 4H, C9–C12 of Cp), 6.79 (s, 7H, C1–7 of Cht). ^13^C NMR (75.5 MHz, CD_3_CN, ppm): δ *=* 13.0 (CH_3_), 77.8 (C9/C12 of Cp), 79.9 (C10/C11 of Cp), 97.8 (C1–7 of Cht), *ipso*-carbon of Cp not visible. ^55^Mn-NMR (74.4 MHz, CD_3_CN, ppm): δ *=* 256.9. IR (ATR, cm^–1^): 3123 (ν_C–H_), 1449 (ν_C=C_), 1016 (ν_C–H_), 820 (ν_P–F_), 556 (ν_P–F_), 447 (ν_metal-ring_). HRMS (ESI pos, *m*/*z*): 225.0460 ([M – PF_6_]^+^), calcd for C_13_H_14_Mn: 225.0471. UV/vis (CH_3_CN, nm): λ_max1_ = 290, λ_max2_ = 545. Single crystals of **11** were obtained from acetonitrile and diethyl ether at room temperature. Single-crystal analysis (Figure 2, Supporting Information).

### η^7^-Cycloheptatrienyl-η^5^-methoxycarbonylcyclopentadienylmanganese (8-Methoxycarbonyltromancenium) Hexafluoridophosphate (**18**)

Analogous to the synthesis of **9** described above, **18** was prepared from methyl cymantrenecarboxylate (**12**)^12^ (0.080 g, 0.305 mmol, 1.00 equiv), 1,3,5-cycloheptatriene (42 μL, 0.382 mmol, 1.25 equiv), and 13 mL of dry heptane. Irradiation with blue light (450 nm) and 370 W for 30 min and subsequent reaction with tritylium hexafluoridophosphate (0.148 g, 0.382 mmol, 1.25 equiv) afforded 8-carbomethoxy-tromancenium hexafluoridophosphate (**18**) as a lavender-colored powder in 19.0% yield (0.024 g, 0.0579 mmol), representing a quantum yield of 1.5%. Mp: 208 °C dec. ^1^H NMR (300.1 MHz, CD_3_CN, ppm): δ *=* 3.92 (s, 3H, CH_3_), 4.87 (s, 2H, C10/C11 of Cp), 5.20 (s, 2H, C9/C12 of Cp), 6.89 (s, 7H, C1–7 of Cht). ^13^C NMR (75.5 MHz, CD_3_CN, ppm): δ *=* 53.7 (CH_3_), 78.6 (C10/C11 of Cp), 80.0 (C9/C12 of Cp), 81.5 (*ipso-*carbon of Cp), 98.8 (C1–7 of Cht), 167.9 (C13 of carbonyl). ^55^Mn-NMR (74.4 MHz, CD_3_CN, ppm): δ *=* 538.2. IR (ATR, cm^–1^): 3652 (_C=O_ overtone), 3122, 3081 (ν_C–H_), 1714 (ν_C=O_), 1476, 1448, 1437, 1409, 1381 (ν_C=C_), 1292 (ν_CO-O_), 1153 (_νO–C–C_), 1034, 965 (ν_ip C–H_), 812 (ν_P–F_), 554 (ν_P–F_), 463 (ν_metal-ring_). HRMS (ESI pos, [*m*/*z*]): 269.0348 ([M - PF_6_]^+^, calcd for C_14_H_14_MnO_2_: 269.0369. UV/vis (CH_3_CN, [nm]): λ_max1_ = 277, λ_max2_ = 569. Single crystals of **18** were obtained from acetonitrile and diethyl ether at room temperature. Single-crystal analysis (Figure 2, Supporting Information).

### η^7^-Cycloheptatrienyl-η^5^-bromocyclopentadienylmanganese (8-Bromotromancenium) Hexafluoridophosphate (**19**)

Analogous to the synthesis of **9** described above, **19** was prepared from bromocymantrene (**13**)^13^ (0.0325 g, 0.115 mmol, 1.00 equiv), 1,3,5-cycloheptatriene (19 μL, 0.172 mmol, 1.50 equiv), and 20 mL of dry heptane. Irradiation with blue light (450 nm) and 370 W for 2.30 min (*Note*: Longer irradiation times lead to undefined mixtures and significant yield loss) and subsequent reaction with tritylium hexafluoridophosphate (0.067 g, 0.172 mmol, 1.50 equiv) afforded 8-bromotromancenium hexafluoridophosphate (**19**) as red crystals in 24.0% yield (0.012 g, 0.028 mmol), representing a quantum yield of 2.7%. Mp: 222 °C dec. ^1^H NMR (300.1 MHz, CD_3_CN, ppm): δ *=* 4.78 (pseudo-t, 2H, J = 2.1 Hz, C10/C11 of Cp), 5.06 (pseudo-t, 2H, J = 2.1 Hz, C9/C12 of Cp), 6.90 (s, 7H, C1–7 of Cht). ^13^C NMR (75.5 MHz, CD_3_CN, ppm): δ *=* 77.3 (C10/C11 of Cp), 81.2 (C9/C12 of Cp), 86.4 (*ipso*-carbon of Cp), 99.4 (C1–7 of Cht). ^55^Mn-NMR (74.4 MHz, CD_3_CN, ppm): δ *=* 368.4. IR (ATR, cm^–1^): 3117 (ν_C–H_), 1447, 1425, 1399, 1364 (ν_C=C_), 1166 , 1025 (ν_C–H_), 808 (ν_P–F_), 554 (ν_P–F_), 448 (ν_metal-ring_). HRMS (ESI pos, *m*/*z*): 288.9421, 290.9398 ([M – PF_6_]^+^), calcd for C_12_H_11_BrMn: 288.9419, 290.9399. UV/vis (CH_3_CN, nm): λ_max1_ = 280, λ_max2_ = 550. Single crystals of **19** were obtained from acetonitrile and diethyl ether at room temperature. Single-crystal analysis (Figure 2, Supporting Information).

### η^7^-Cycloheptatrienyl-η^5^-aminocyclopentadienylmanganese (8-Aminotromancenium) Hexafluoridophosphate (**20**)

Analogous to the synthesis of **9** described above, **20** was prepared from 0.124 g of aminocymantrene (**14**)^15^ (0.565 mmol, 1.00 equiv), 75 μL of 1,3,5-cycloheptatriene (0.680 mmol, 1.20 equiv), and 10 mL of toluene (abs). Irradiation with blue light (450 nm) and 370 W for 3.40 min (*Note:* A chemoselective synthesis of **20** requires a meticulous control of the irradiation time, since excessive photochemical excitation leads to cleavage of the C–N bond, resulting in the formation of unsubstituted tromancenium salt) and subsequent reaction with tritylium hexafluoridophosphate (0.262 g, 0.675 mmol, 1.20 equiv) afforded air-stable 8-aminotromancenium hexafluoridophosphate (**20**) as red crystals in 9.5% yield (0.020 g, 0.054 mmol) with a quantum yield of 8.4%. Mp: 245 °C dec. ^1^H NMR (300.1 MHz, CD_3_CN, ppm): δ *=* 4.08 (s, 2H, NH_2_), 4.32 (pseudo-t, 2H, J = 2.1 Hz, C9/C12 of Cp), 4.43 (pseudo-t, 2H, J = 1.8 Hz, C10/C11 of Cp), 6.63 (s, 7H, C1–7 of Cht). ^13^C NMR (75.5 MHz, CD_3_CN, ppm): δ *=* 65.3 (C9/C12 of Cp), 73.3 (C10/C11 of Cp), 97.5 (C1–7 of Cht), 123.9 (*ipso*-carbon of Cp). ^55^Mn-NMR (74.4 MHz, CD_3_CN, ppm): δ *=* 238.0. IR (ATR, cm^–1^): 3502, 3406 (ν_N–H_), 3076 (ν_C–H_), 1710, 1628 (ν_C=C_), 1074, 1054, 1033 (δ_C–H_), 826 (ν_P–F_), 555 (δ_P–F_), 451, 437 (ν_metal-ring_). MS (ESI pos, [*m*/*z*]): 226.0419 ([M – PF_6_]^+^), calcd for C_14_H_14_MnO_2_: 226.0423. UV/vis (CH_3_CN, nm): λ_max1_ = 288, λ_max2_ = 312, λ_max3_ = 500 (plateau). Single crystals of **20** were obtained from acetonitrile and diethyl ether at room temperature. Single-crystal analysis (Figure 2, Supporting Information).

## Supporting Information Available

Spectra (^1^H/^13^C/^55^Mn NMR, IR, HRMS, UV–vis), photoelectron spectra (XPS), cyclic voltammograms, DFT results (PDF)

## Accession Codes

CCDC 2079152–2079157 contain the supplementary crystallographic data for this paper. These data can be obtained free of charge via www.ccdc.cam.ac.uk/data_request/cif, or by emailing data_request@ccdc.cam.ac.uk, or by contacting The Cambridge Crystallographic Data Centre, 12 Union Road, Cambridge CB2 1EZ, UK; fax: +44 1223 336033.
