# Supplementary material for: Cationic Cycloheptatrienyl Cyclopentadienyl Manganese Sandwich Complexes: Tromancenium Explored with High-Power LED Photosynthesis
Source: Organometallics. 2021 Jul 27;40(15):2736–49. doi: 10.1021/acs.organomet.1c00376 (PMC8356223; doi:10.1021/acs.organomet.1c00376)
Supplement: Supplementary file 1 — om1c00376_si_001.pdf [file om1c00376_si_001.pdf]

Supporting Information

Cationic cycloheptatrienyl cyclopentadienyl manganese sandwich complexes: tromancenium explored with high-power LED photosynthesis

Reinhard Basse,[#] Stefan Vanicek,[#] Thomas Höfer,[#] Holger Kopacka,[#] Klaus Wurst,[#] Thomas Müller,[◊] Heidi A. Schwartz,[#] Selina Olthof,[&] Larissa A. Casper,[÷] Moritz Nau,[÷] Rainer F. Winter,*[,÷] Maren Podewitz,*[,#] and Benno Bildstein*[,#]

[#]Institute of General, Inorganic and Theoretical Chemistry, Center for Chemistry and Biomedicine, University of Innsbruck, Innrain 80-82, 6020 Innsbruck, Austria

[◊]Institute of Organic Chemistry, Center for Chemistry and Biomedicine, University of Innsbruck, Innrain 80-82, 6020 Innsbruck, Austria

[&]Department of Chemistry, University of Cologne, Luxemburger Str. 116, 50939 Köln, Germany

[÷]Department of Chemistry, University of Konstanz, Universitätsstrasse 10, 78457 Konstanz, Germany

*E-mail for B.B.: benno.bildstein@uibk.ac.at
*E-mail for M.P.: Maren.Podewitz@uibk.ac.at
*E-mail for R.F.W.: rainer.winter@uni-konstanz.de

1. **Analytical Section** ... 2

Tromancenium hexafluoridophosphate (9) ... 2

1-Methyltromancenium hexafluoridophosphate (10) ... 5

8-Methyltromancenium hexafluoridophosphate (11) ... 8

8-Methoxycarbonyltromancenium hexafluoridophosphate (18) ... 12

8-Bromotromancenium hexafluoridophosphate (19) ... 15

8-Aminotromancenium hexafluoridophosphate (20) ... 18

2. **XPS measurements** ... 21

3. **Cyclic Voltammetry** ... 24

4. **Density Functional Theory Calculations** ... 33

1. Analytical Section

Tromancenium hexafluoridophosphate (9)

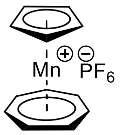

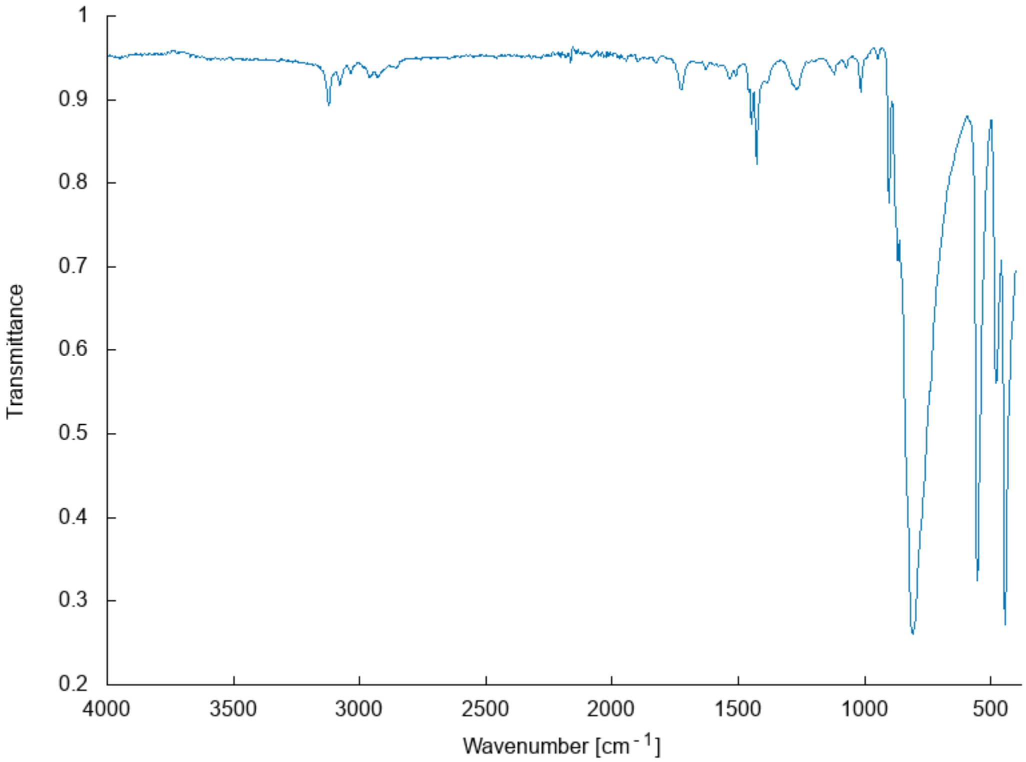

Figure S1. IR spectrum of **9**.

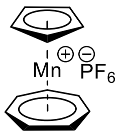

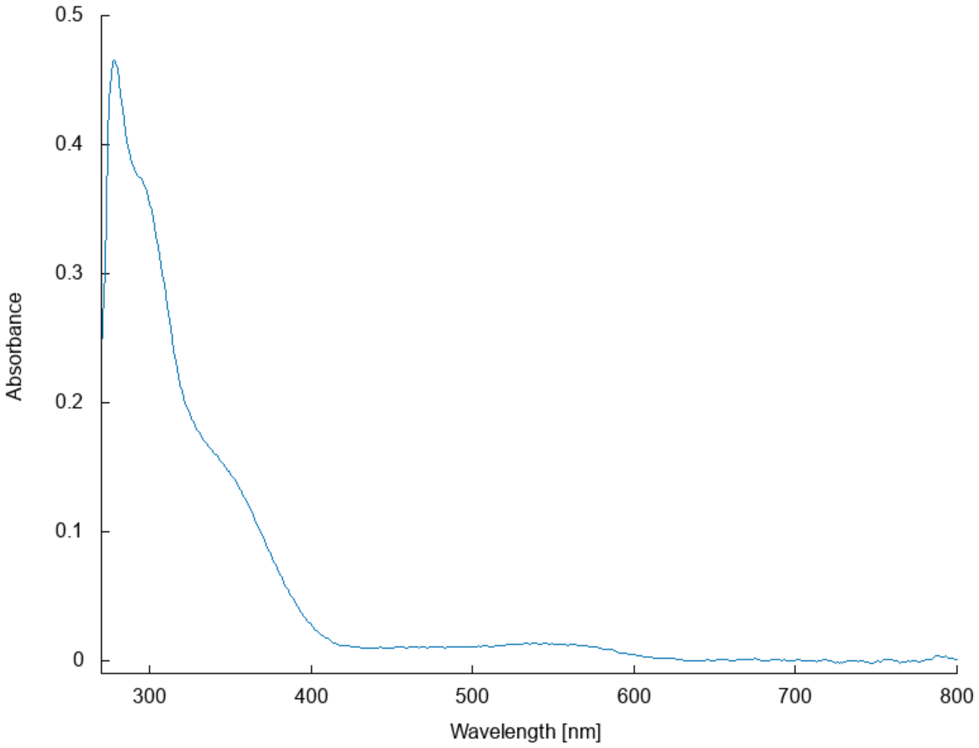

Figure S2. UV/vis spectrum of **9**.

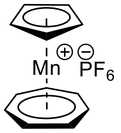

Mn PF_6

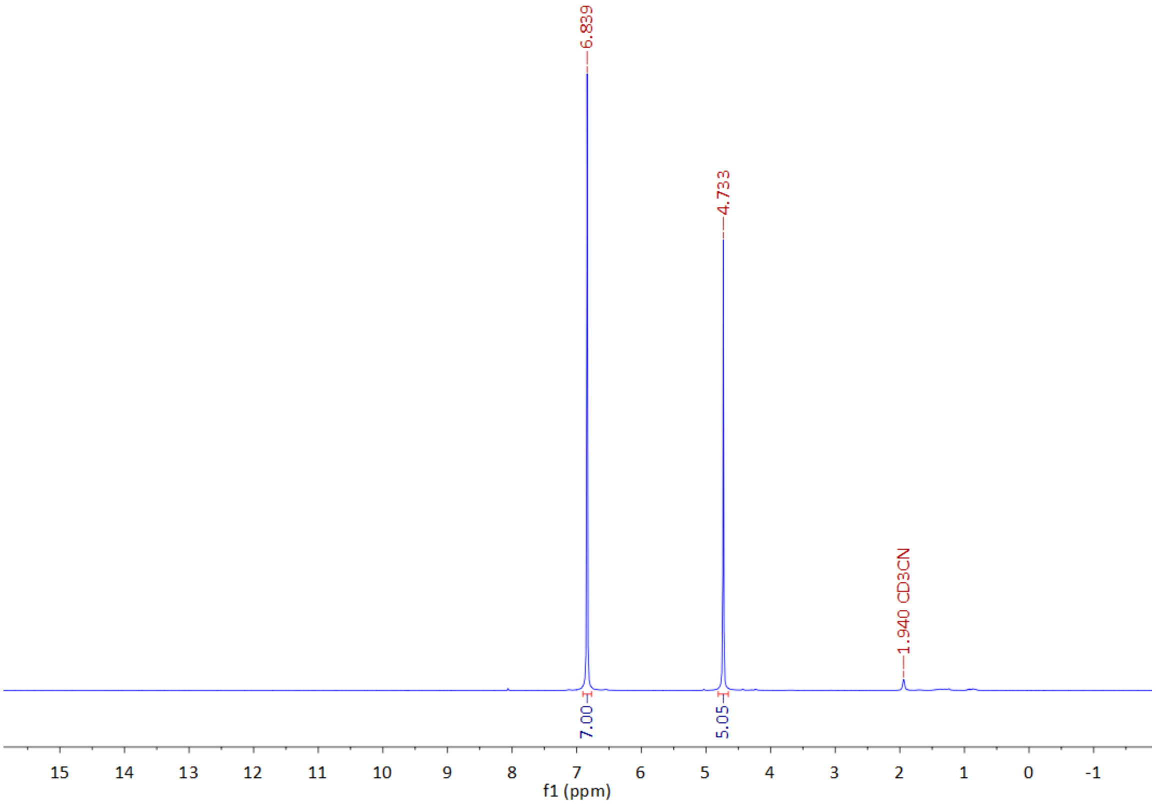

Figure S3. ^{1}H-NMR spectrum of **9**.

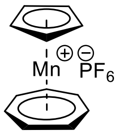

Mn PF_6

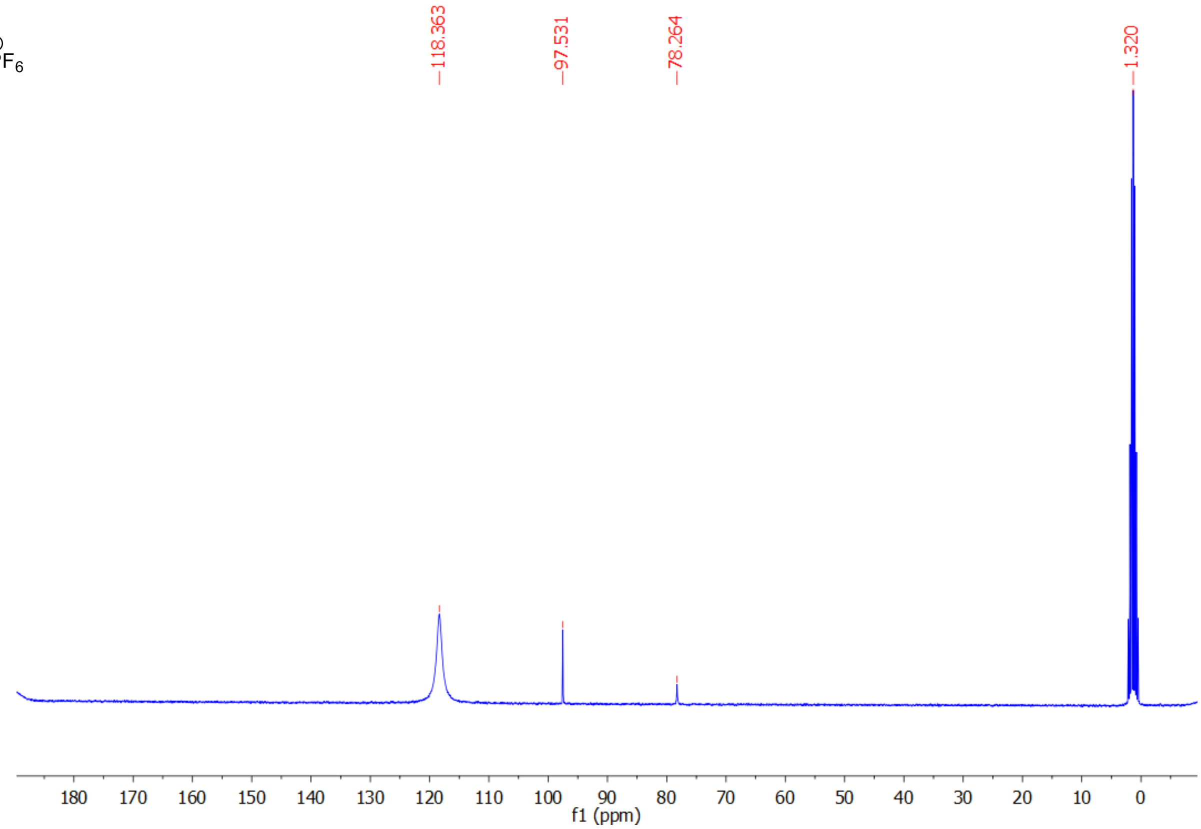

Figure S4. ^{13}C-NMR spectrum of **9**.

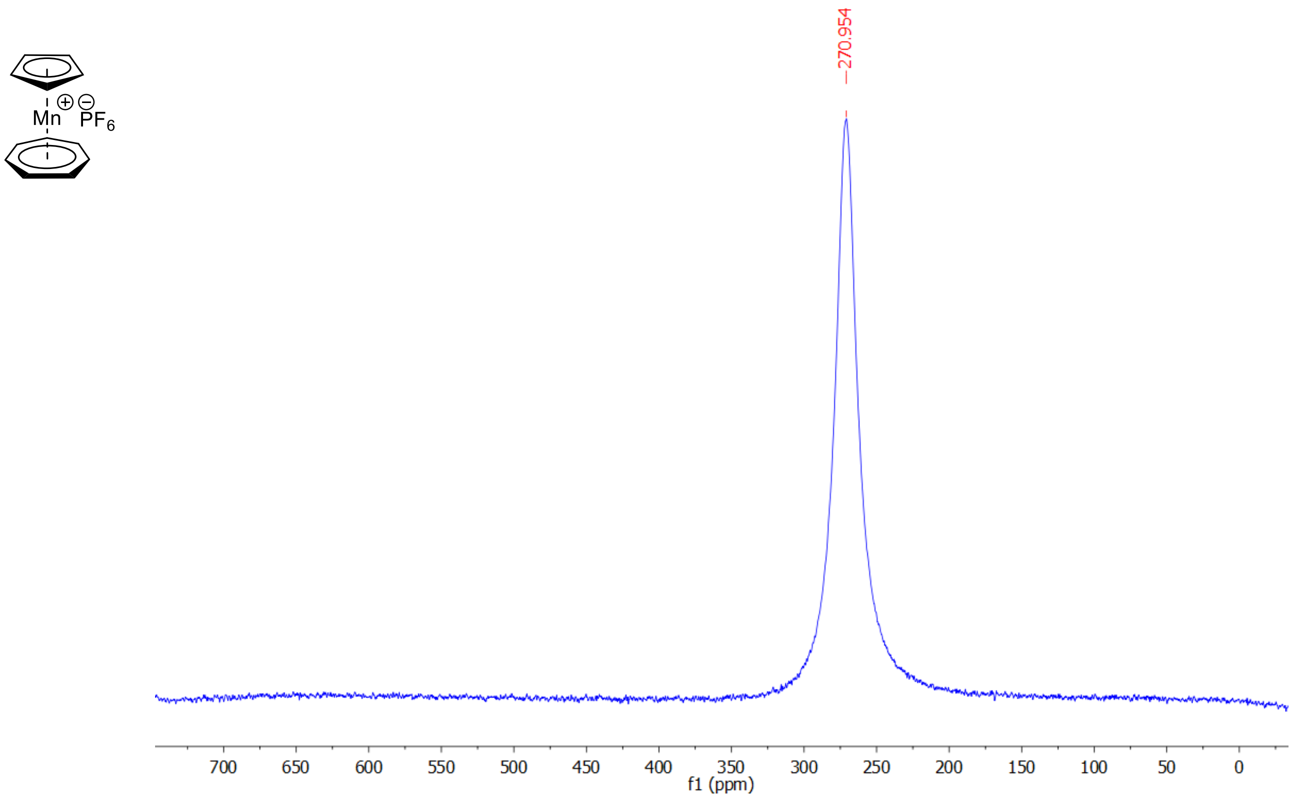

Figure S5. ^{55}Mn-NMR spectrum of **9**.

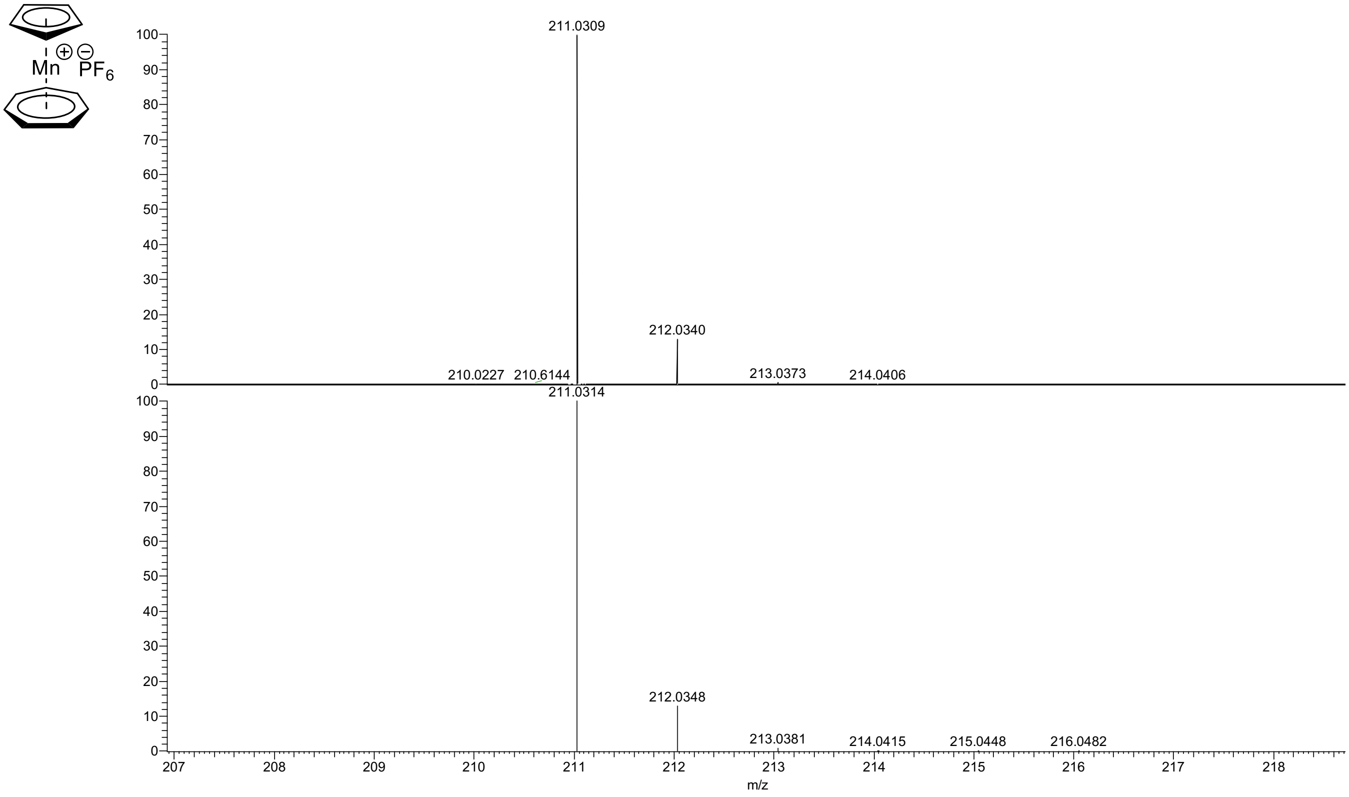

Figure S6. MS (ESI pos, [m/z]; *top*: experimental, *bottom*: simulated) of **9**.

1-Methyltromancenium hexafluoridophosphate (10)

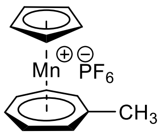

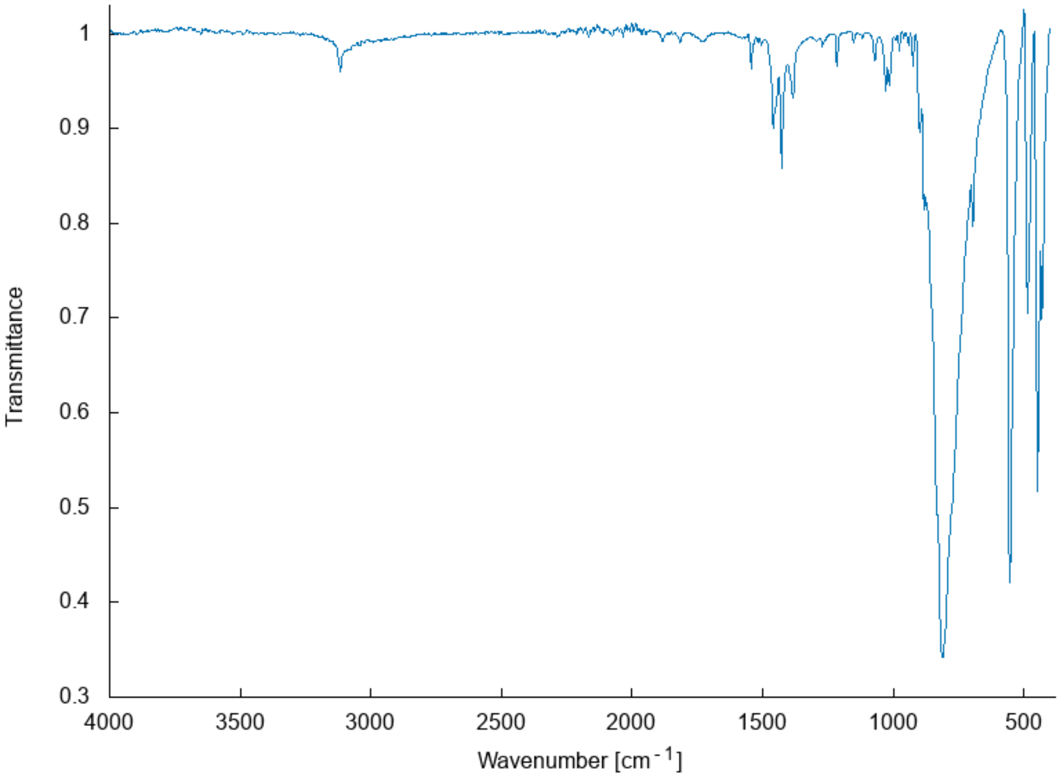

Figure S7. IR spectrum of **10**.

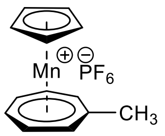

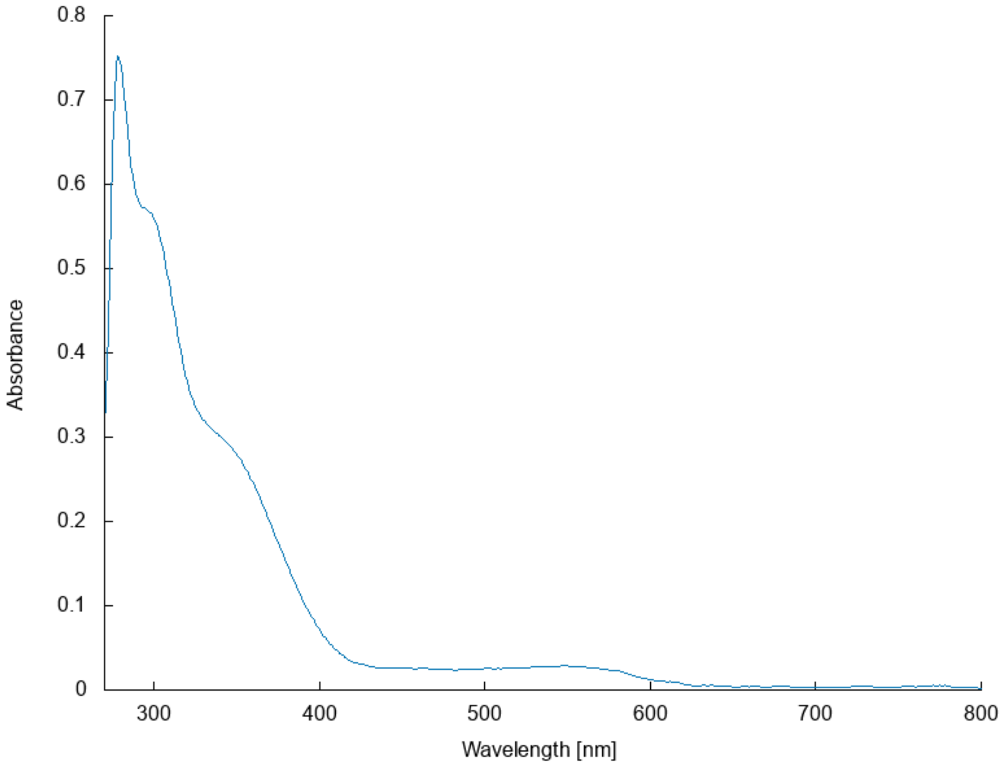

Figure S8. UV/vis spectrum of **10**.

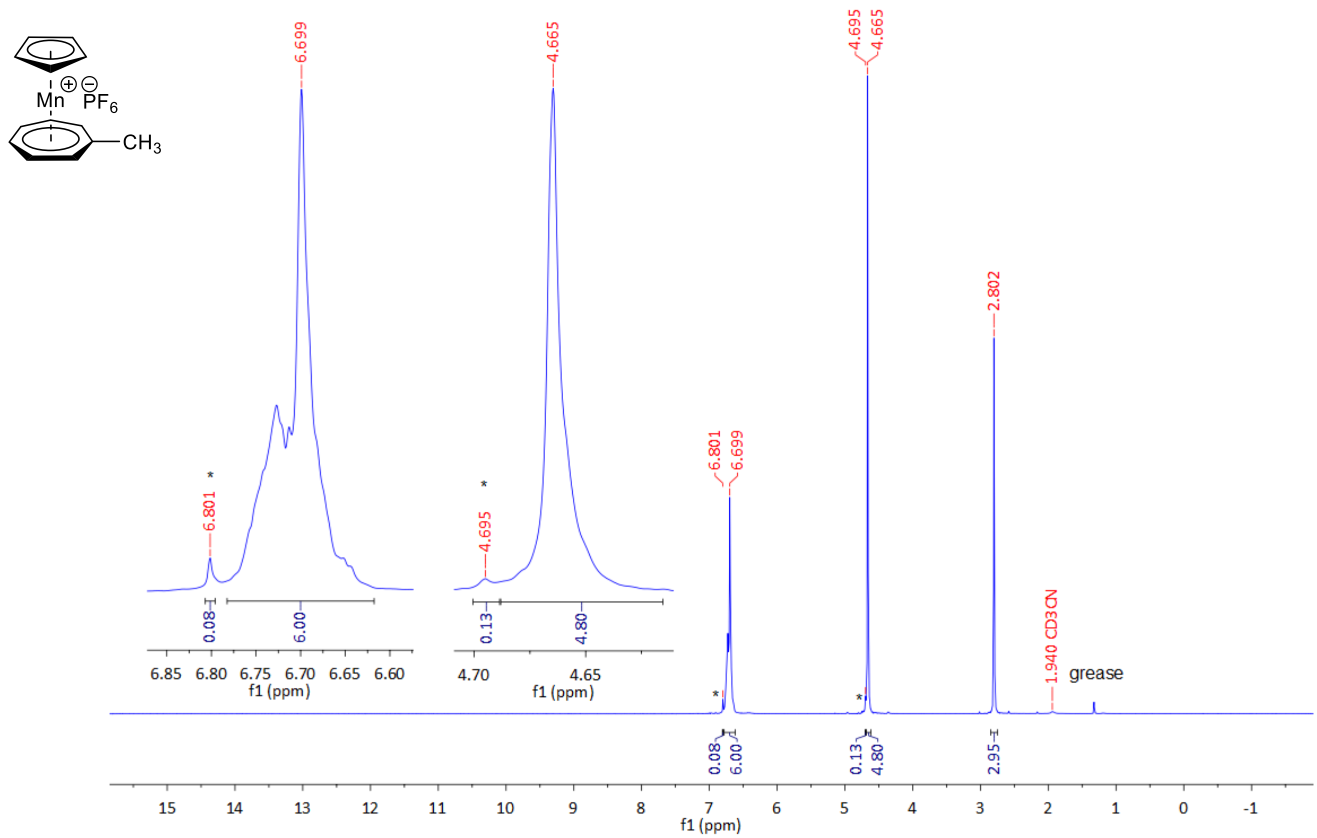

Figure S9. ¹H-NMR spectrum of **10** (*marked:* traces of unsubstituted tromancenium salt **9**).

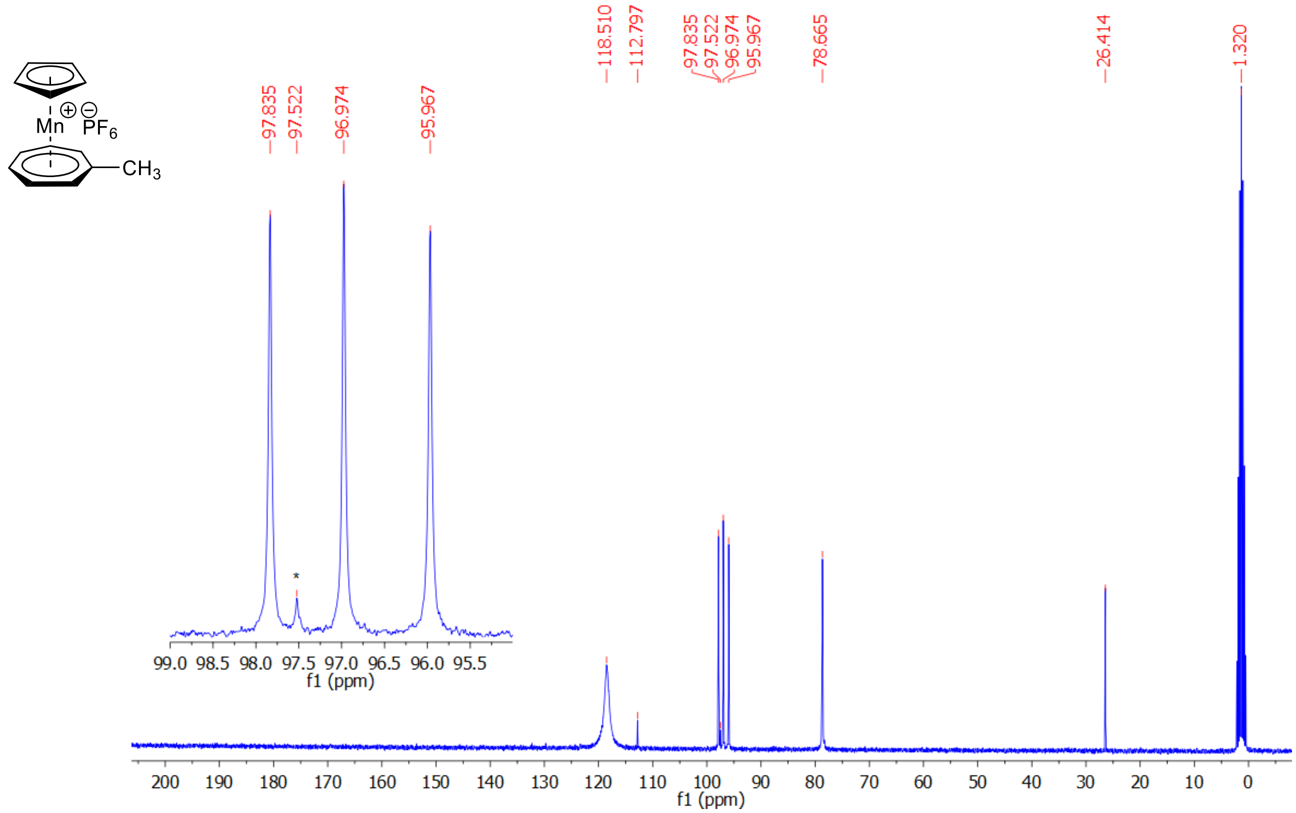

Figure S10. ¹³C-NMR spectrum of **10** (*marked:* traces of unsubstituted tromancenium salt **9**).

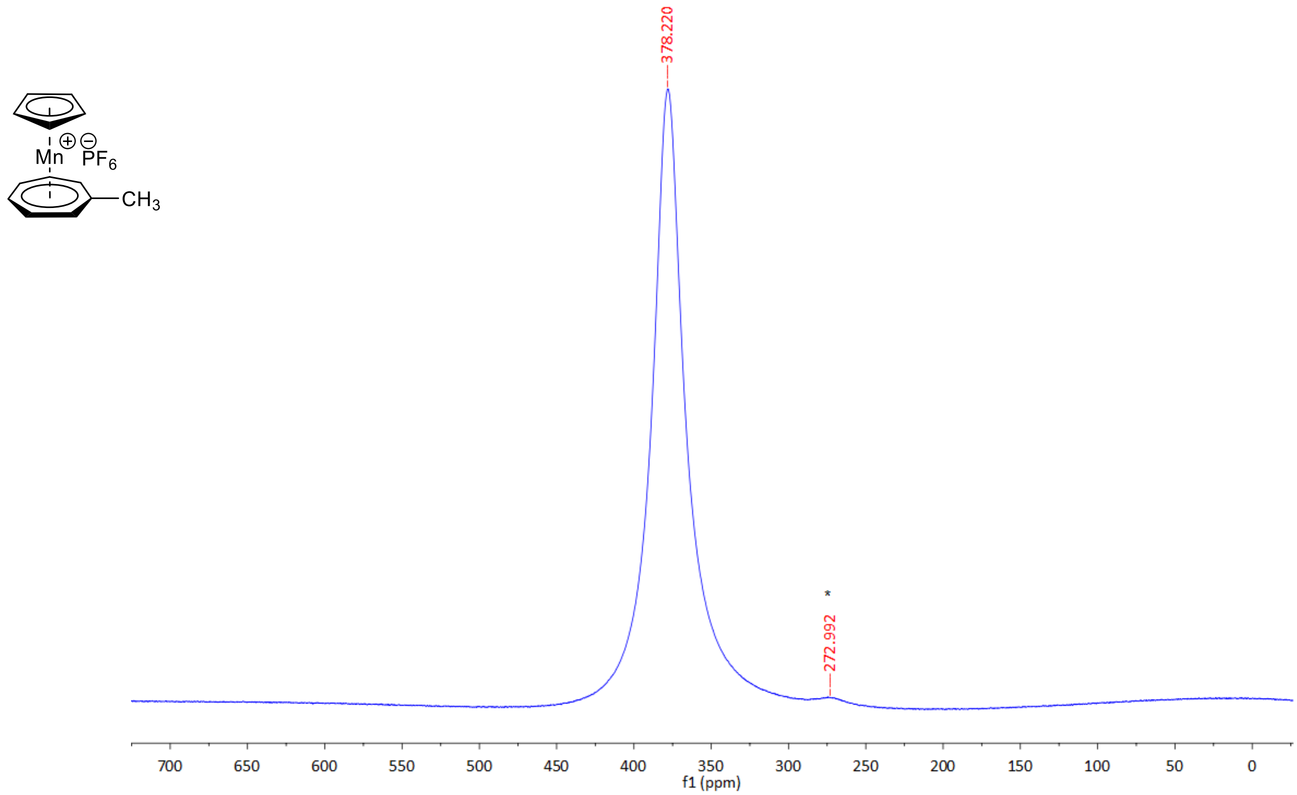

Figure S11. [55]Mn-NMR spectrum of **10** (*marked:* traces of unsubstituted tromancenium salt **9**).

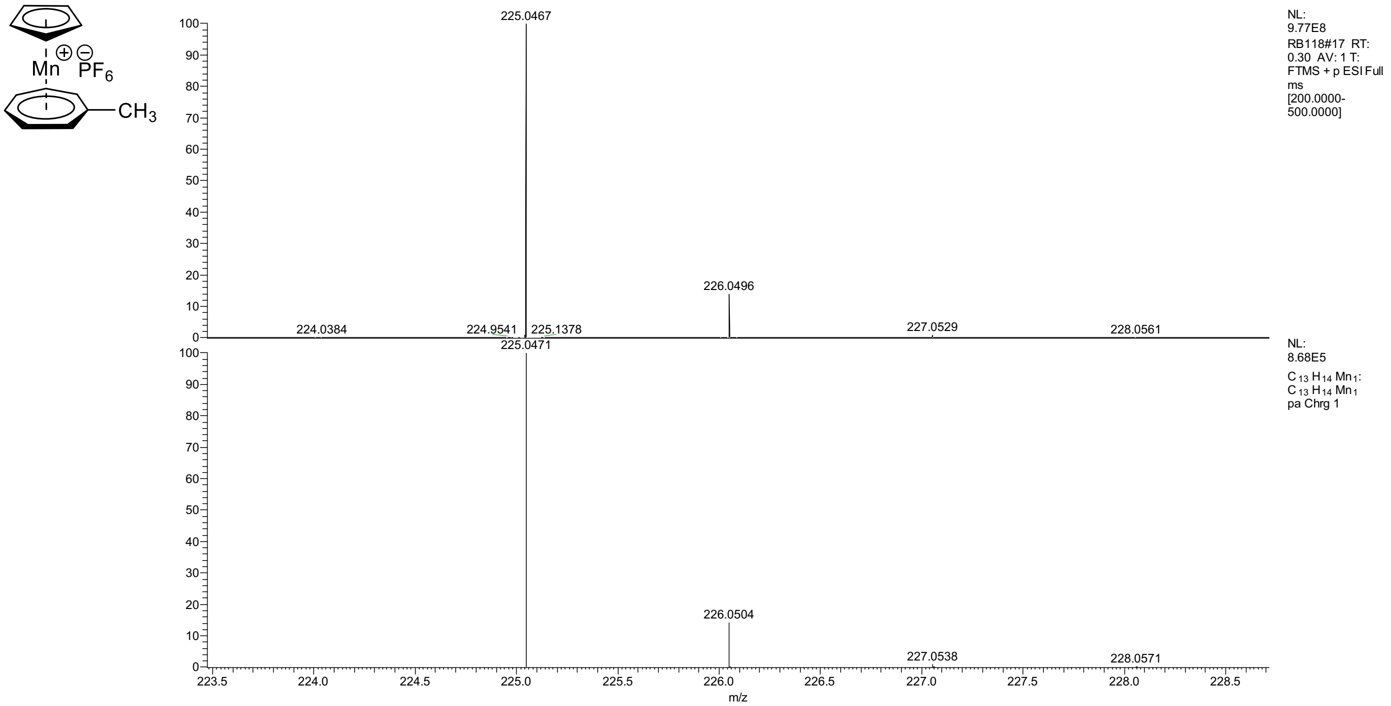

Figure S12. MS (ESI pos, [m/z]; *top*: experimental, *bottom*: simulated) of **10**.

8-Methyltromancenium hexafluoridophosphate (11)

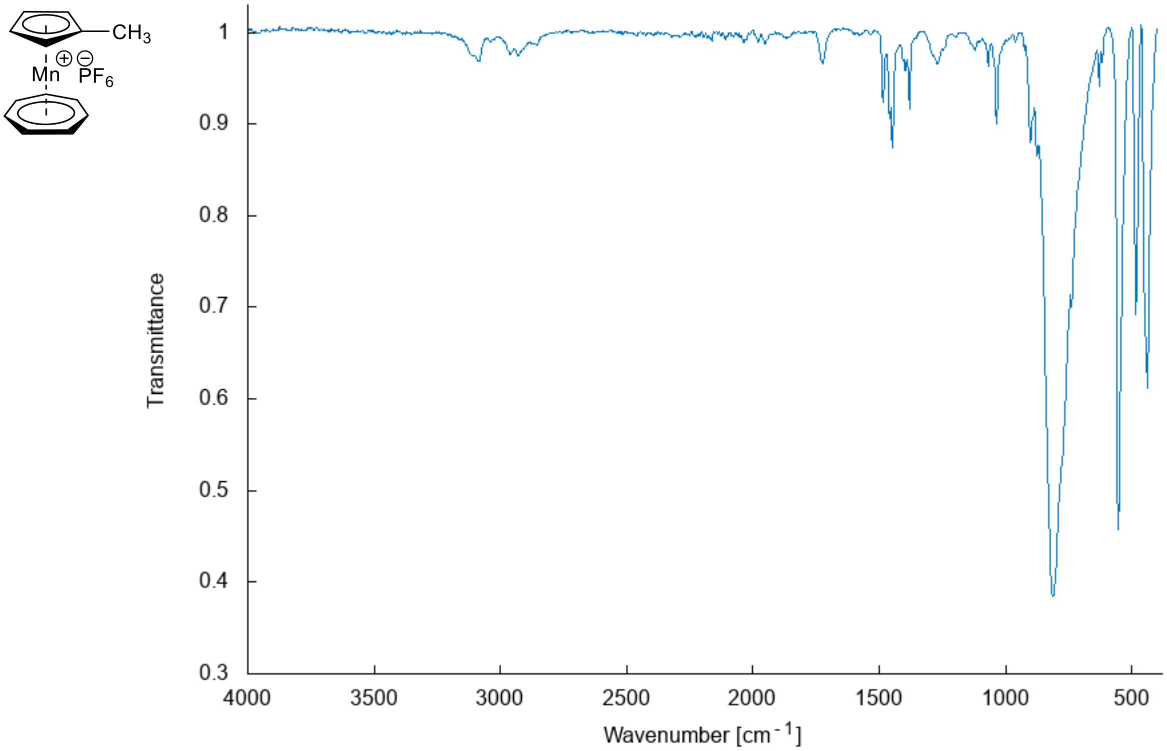

Figure S13. IR spectrum of **11**.

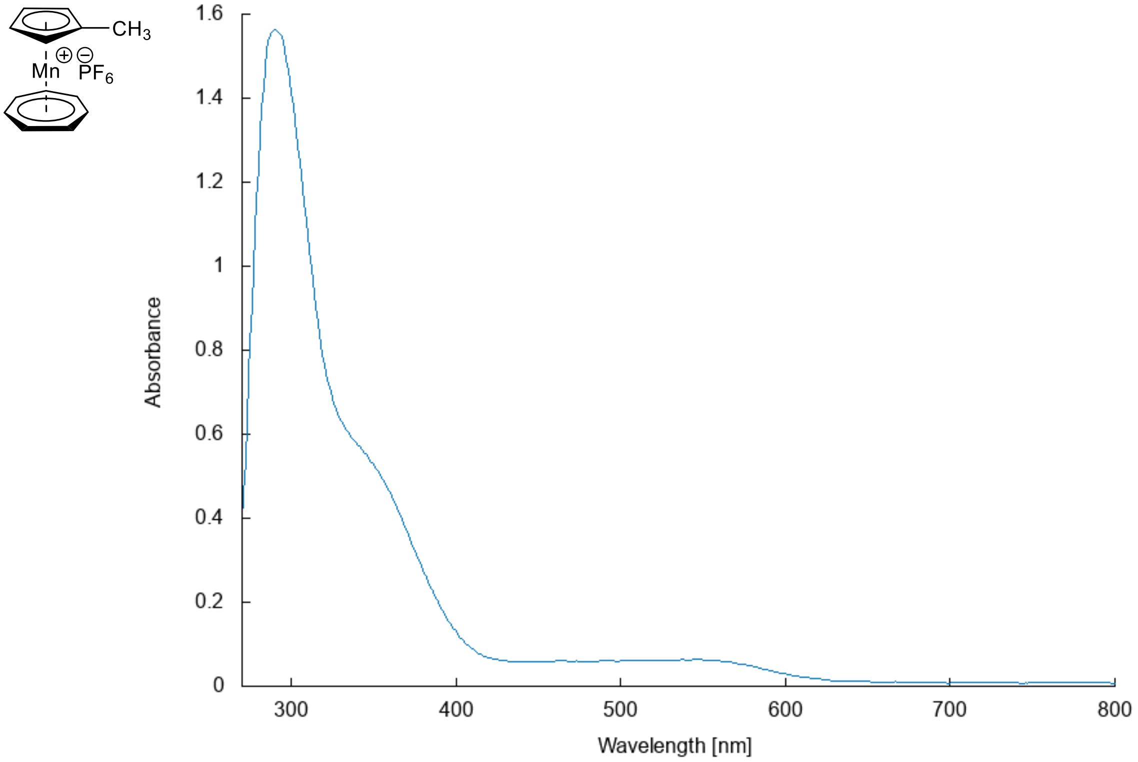

Figure S14. UV/vis spectrum of **11**.

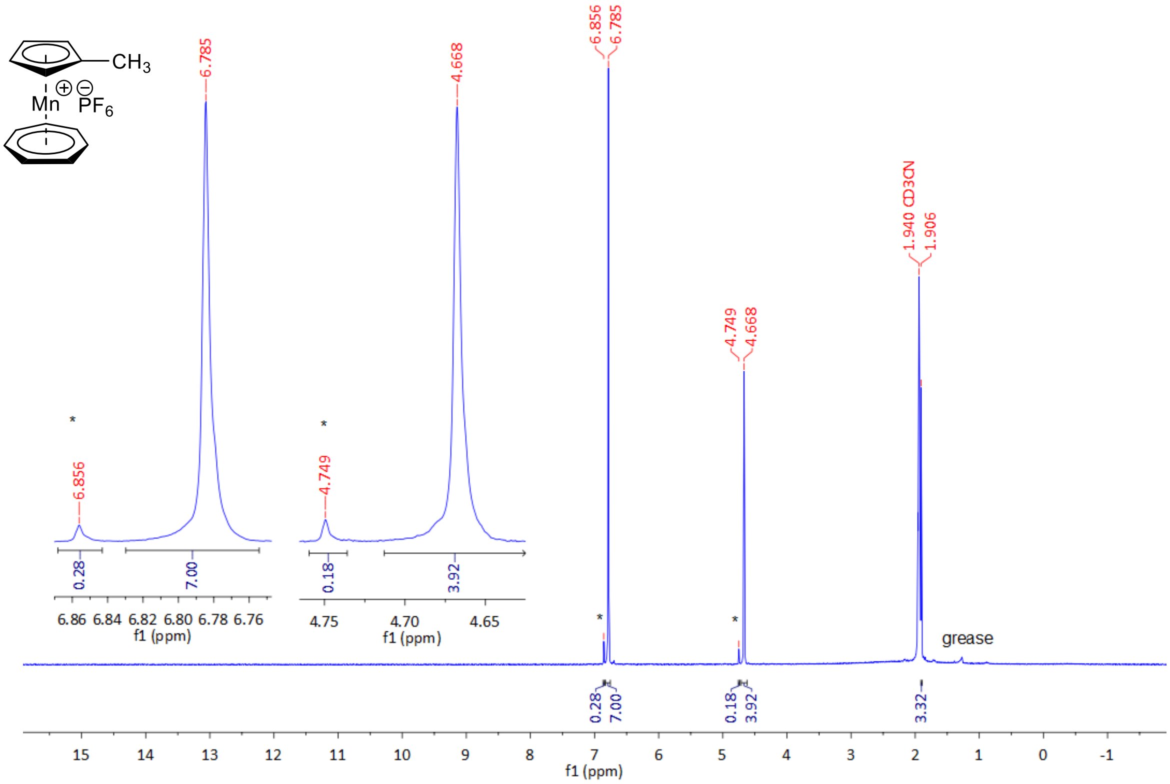

Figure S15. ^{1}H-NMR spectrum of **11** (*marked:* traces of unsubstituted tromancenium salt **9**).

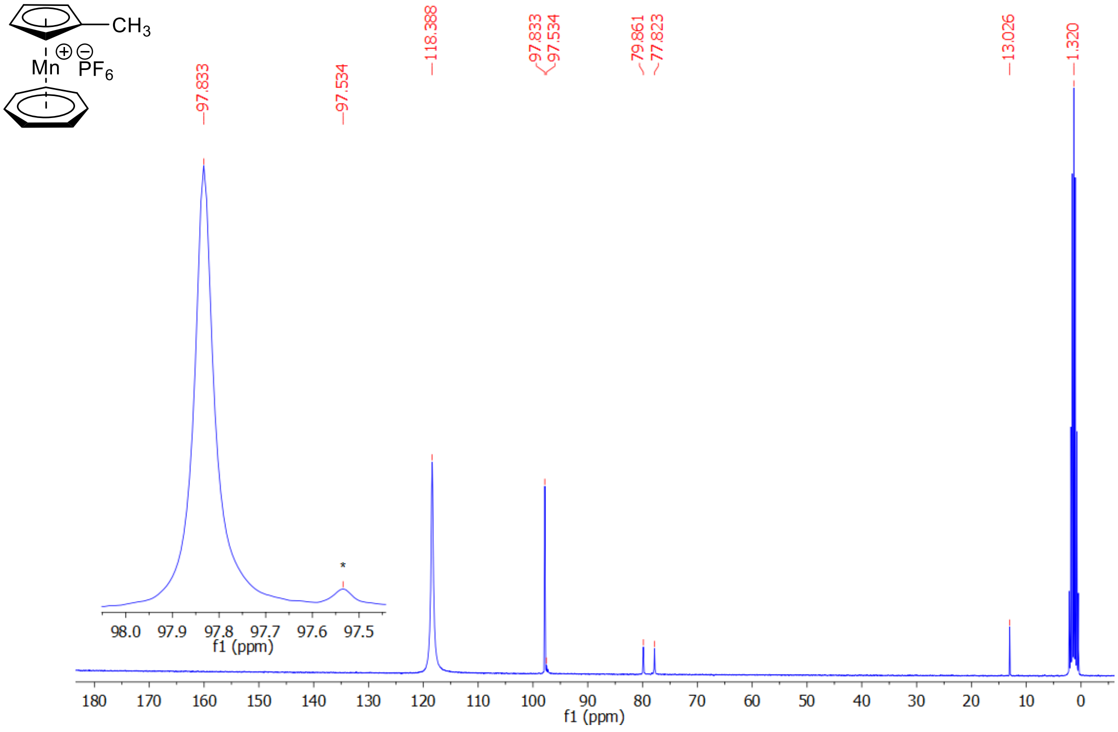

Figure S16. ^{13}C-NMR spectrum of **11** (*marked:* traces of unsubstituted tromancenium salt **9**).

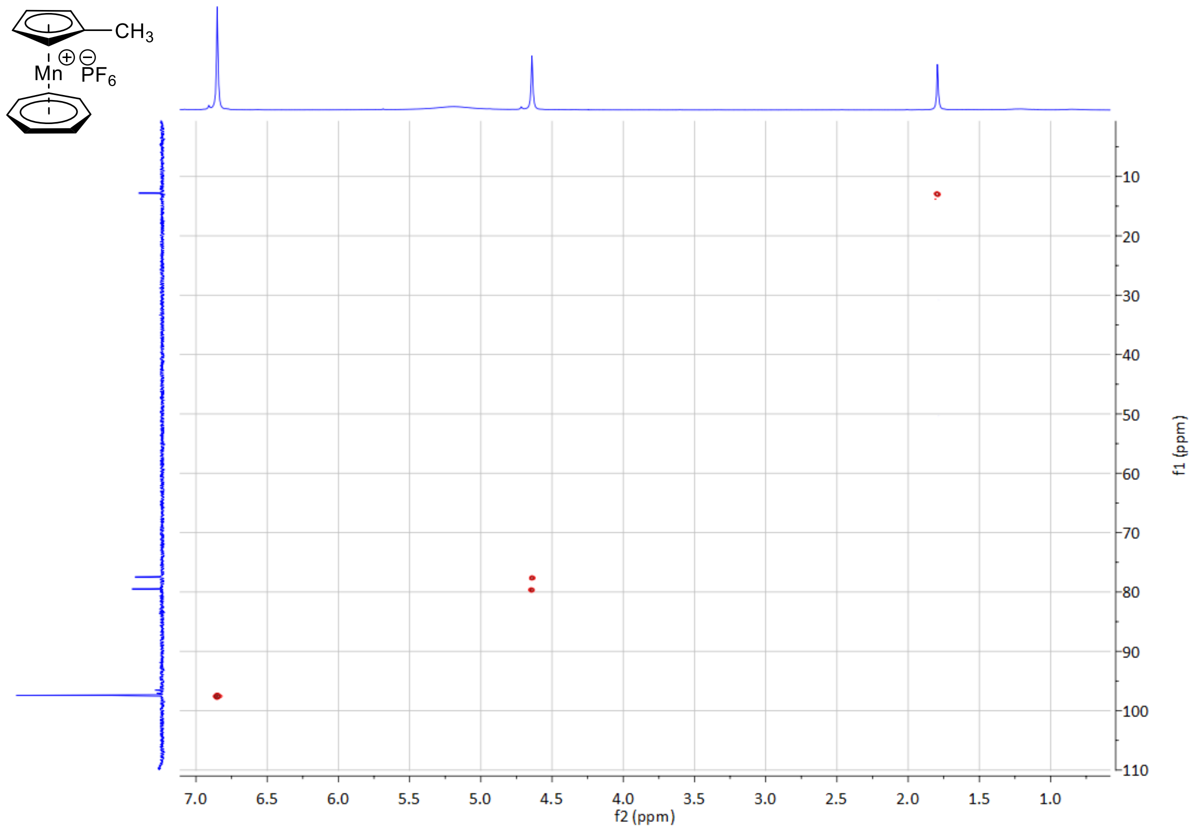

Figure S17. HMQC spectrum of **11** in CD_3CN.

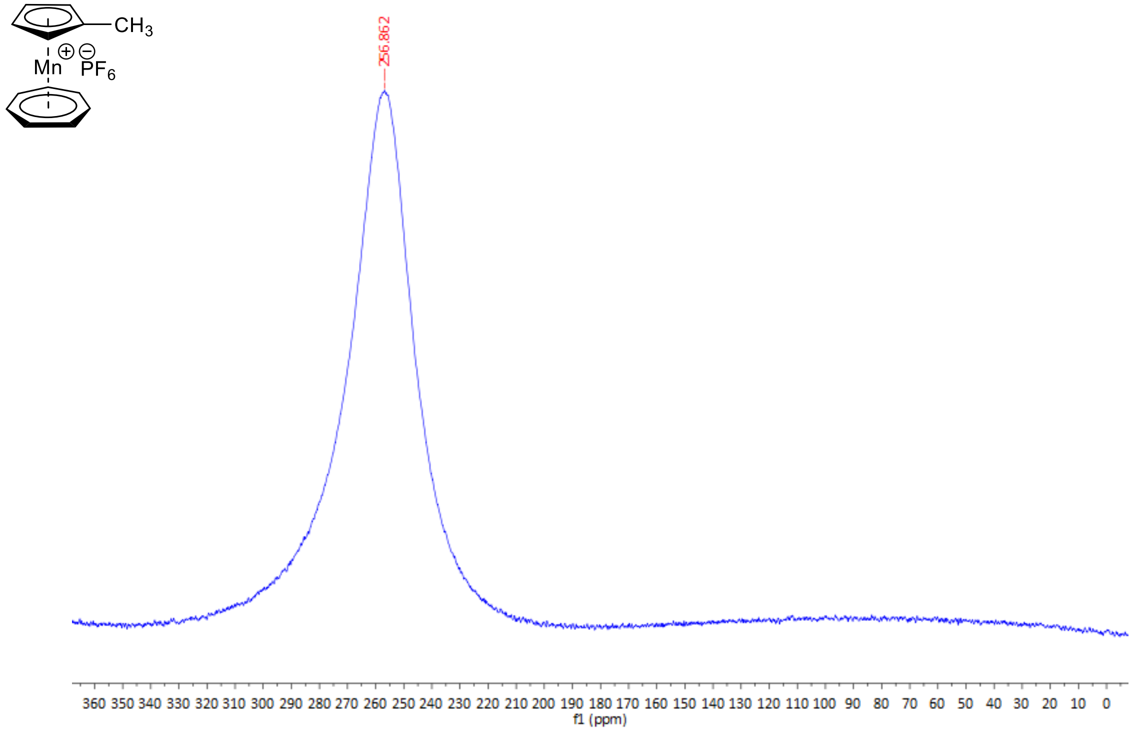

Figure S18. ^{55}Mn-NMR spectrum of **11**.

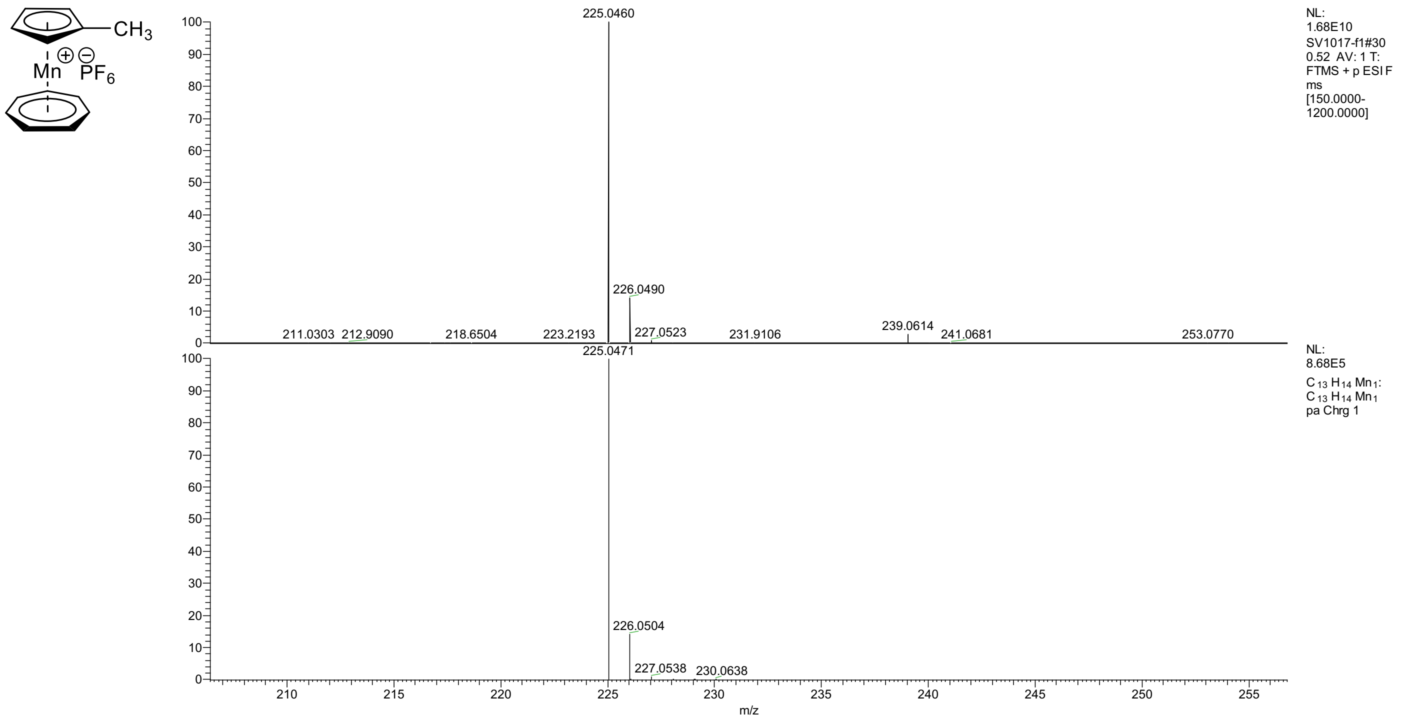

Figure S19. MS (ESI pos, [m/z]; *top*: experimental, *bottom*: simulated) of **11**.

8-Methoxycarbonyltromancenium hexafluoridophosphate (18)

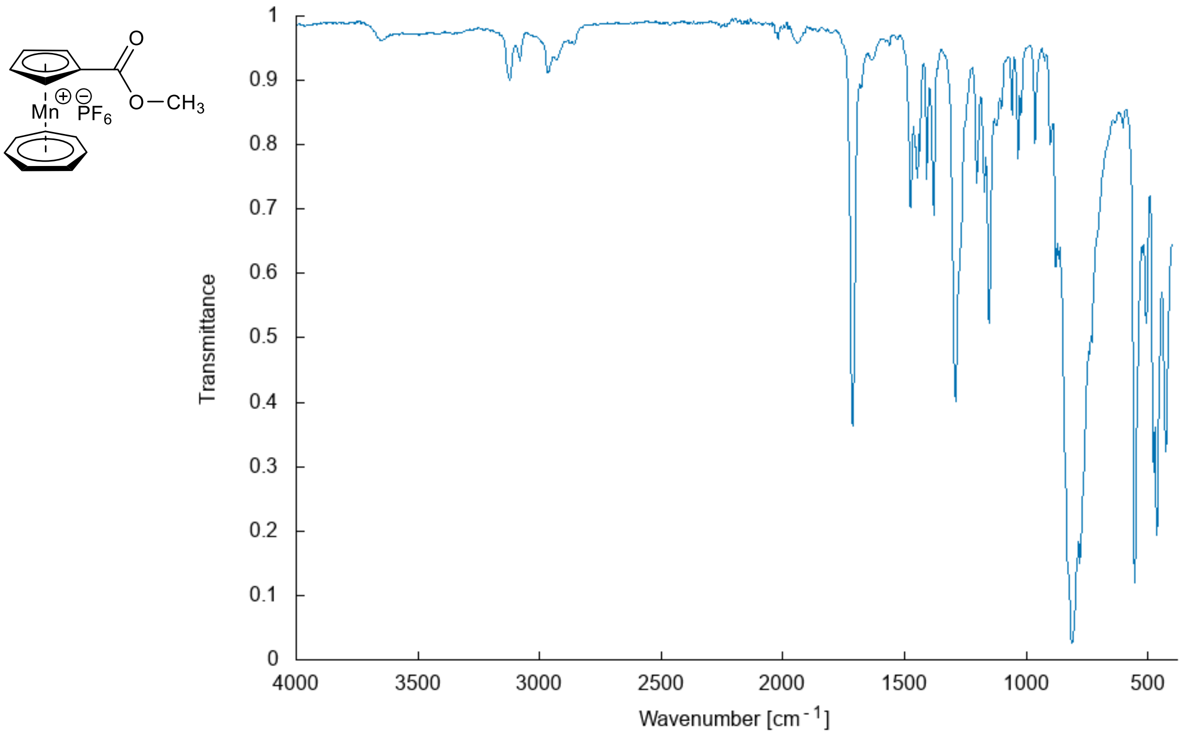

Figure S20. IR spectrum of **18**.

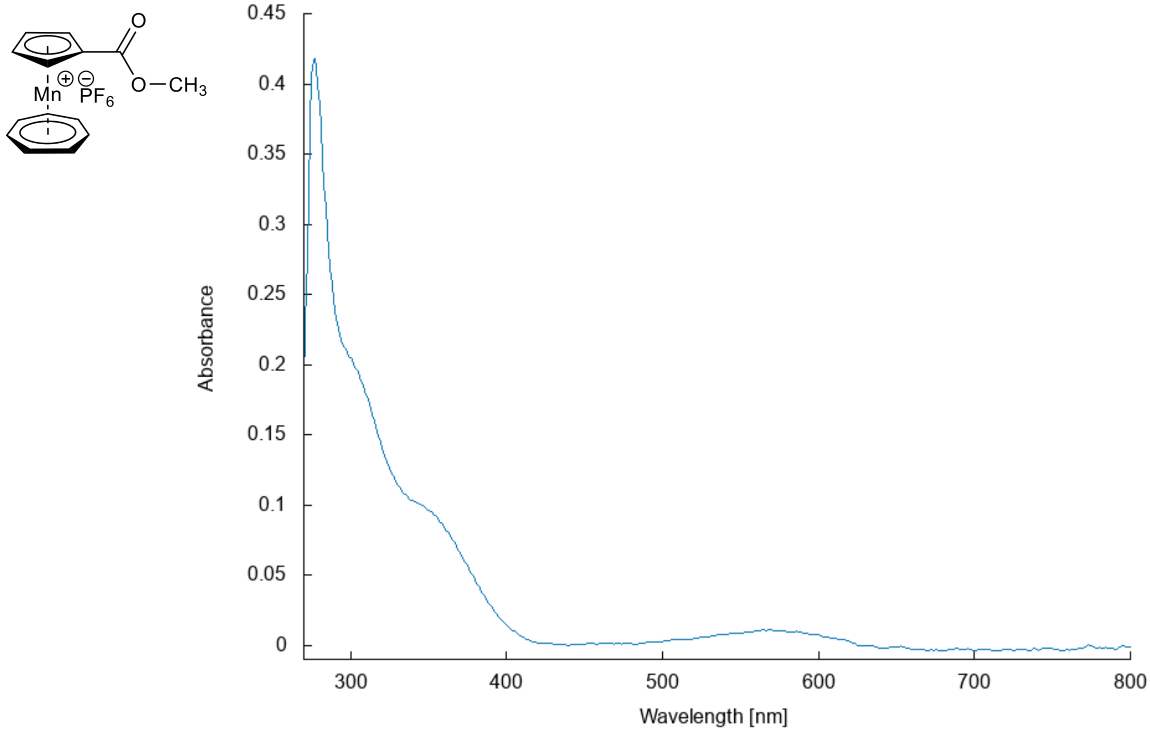

Figure S21. UV/vis spectrum of **18**.

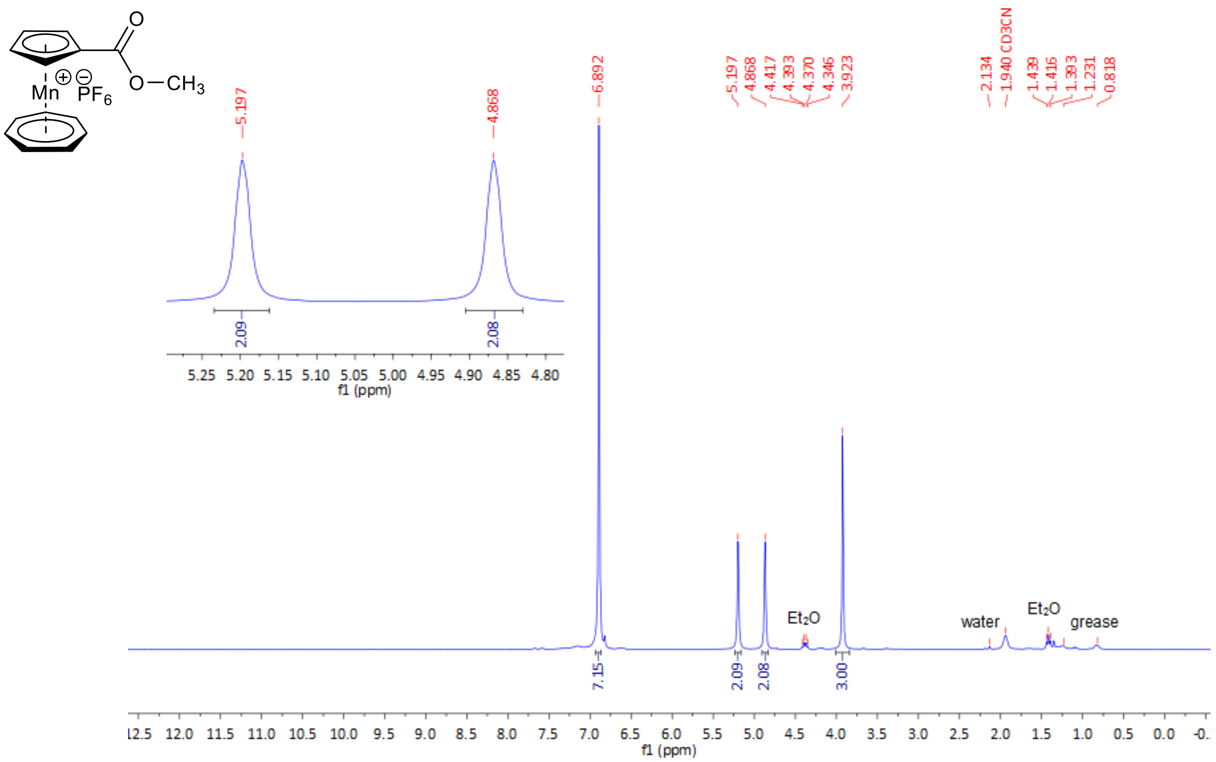

Figure S22. ^{1}H-NMR spectrum of **18**.

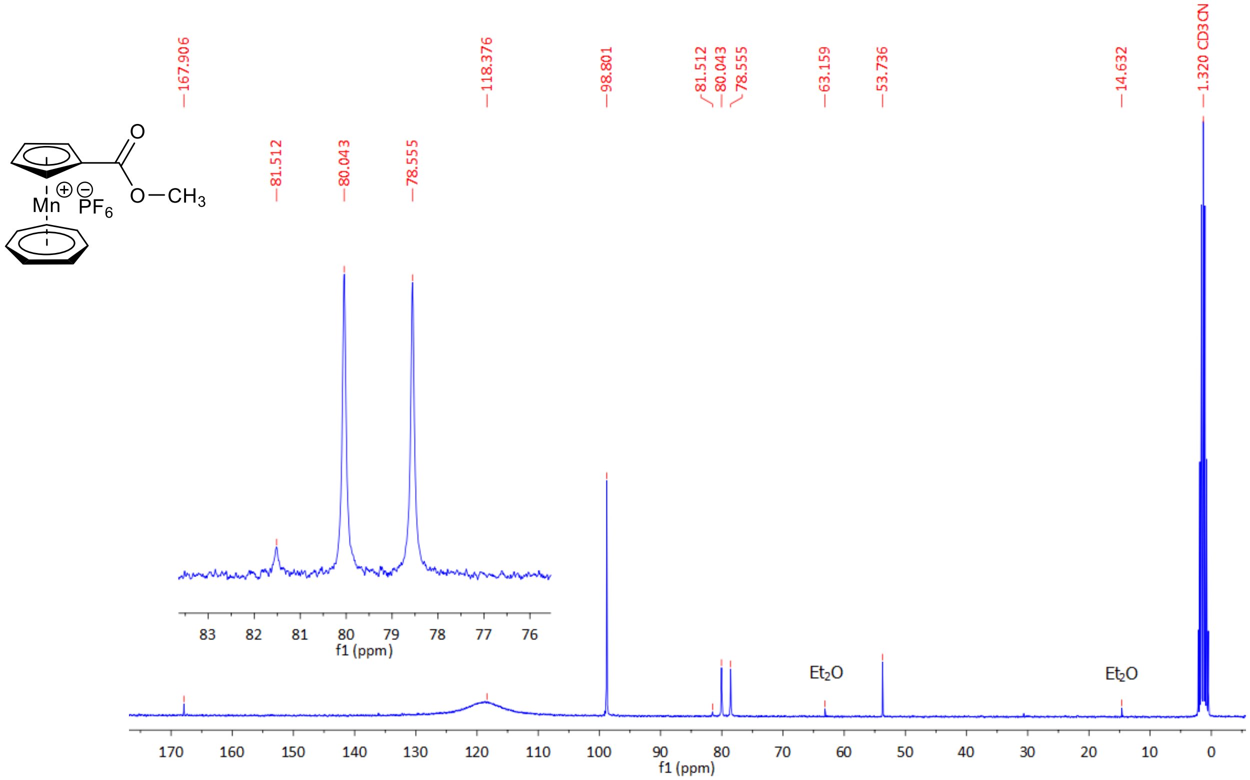

Figure S23. ^{13}C-NMR spectrum of **18**.

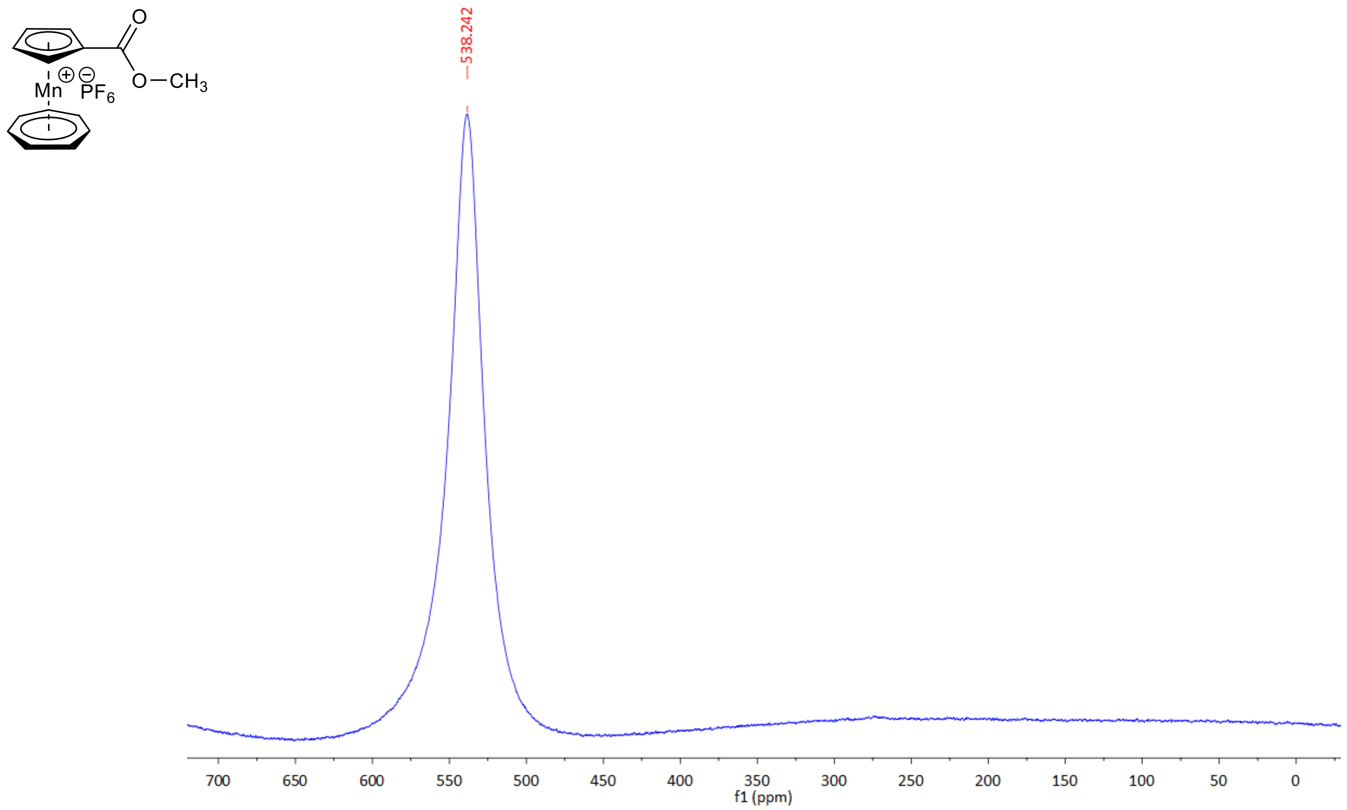

Figure S24. ^{55}Mn-NMR spectrum of **18**.

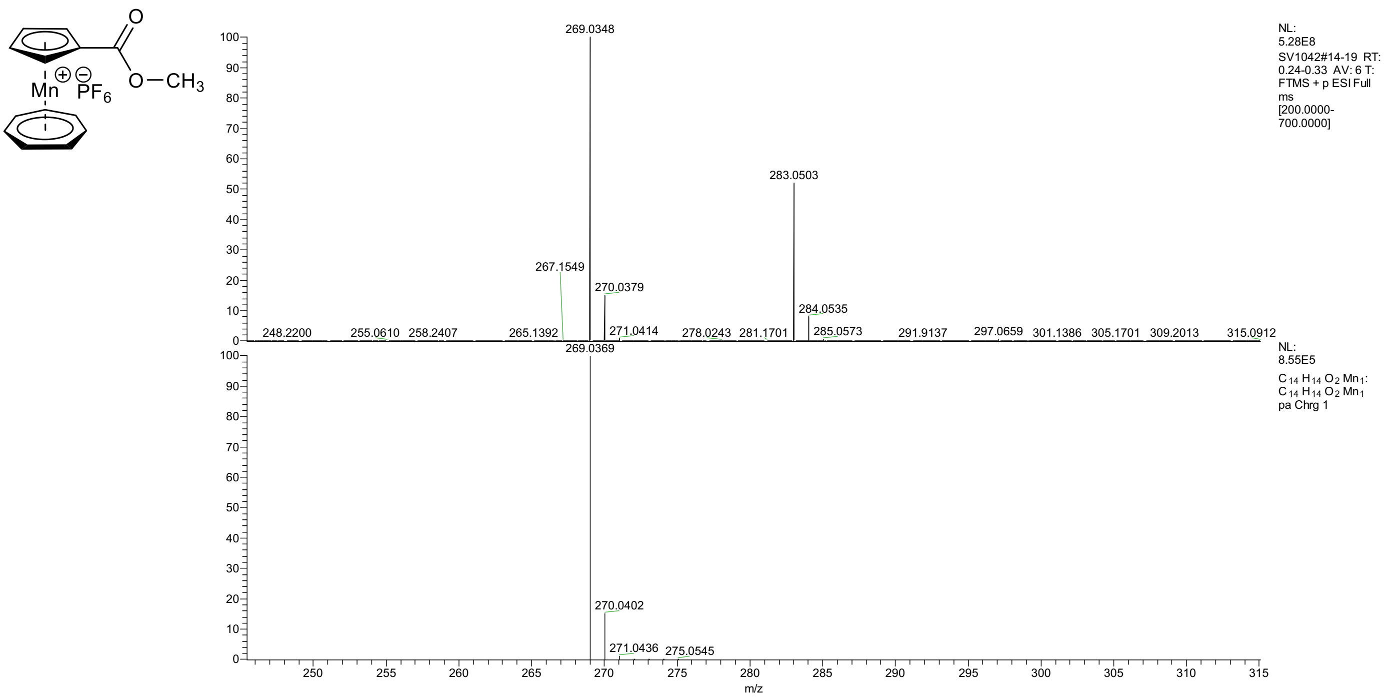

Figure S25. MS (ESI pos, [m/z]; *top*: experimental, *bottom*: simulated) of **18**.

8-Bromotromancenium hexafluoridophosphate (19)

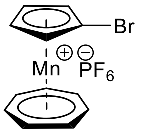

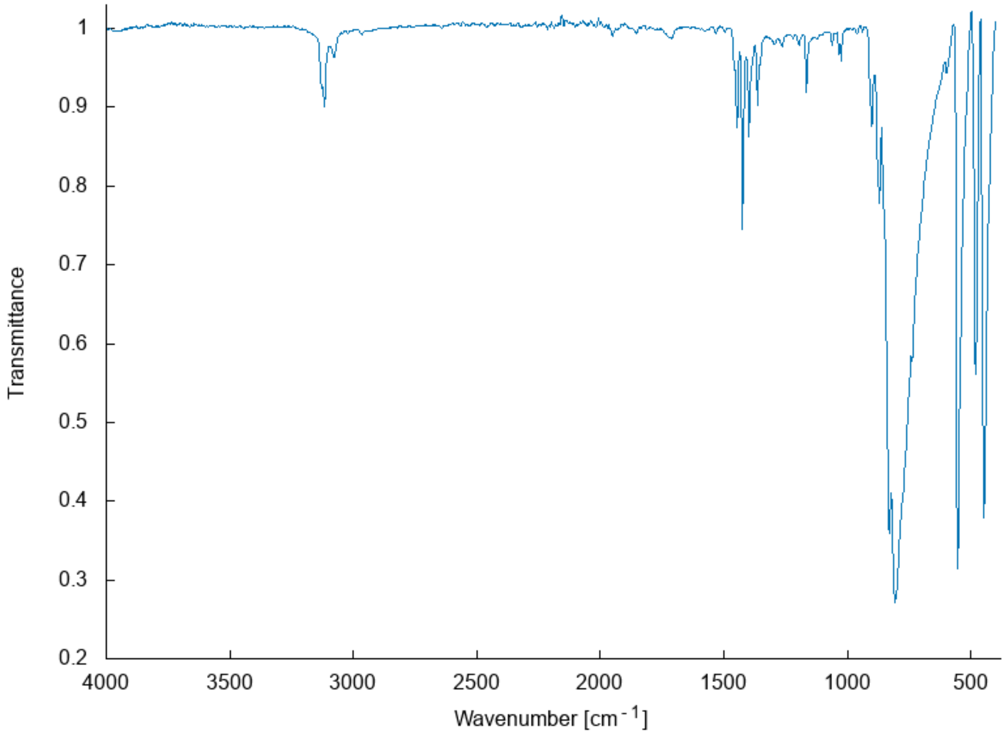

Figure S26. IR spectrum of **19**.

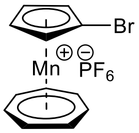

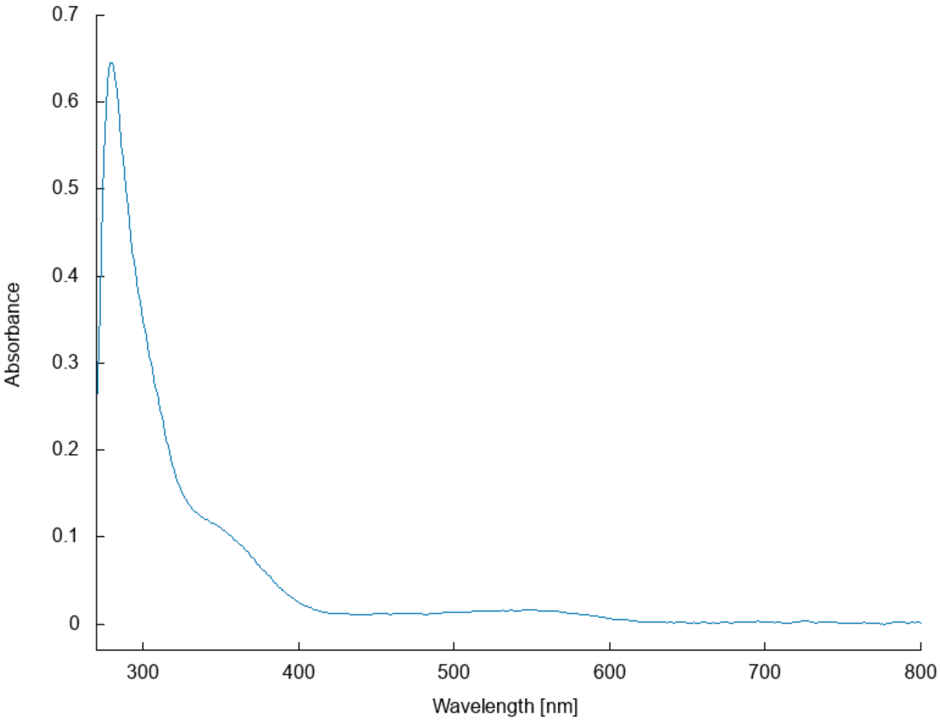

Figure S27. UV/vis spectrum of **19**.

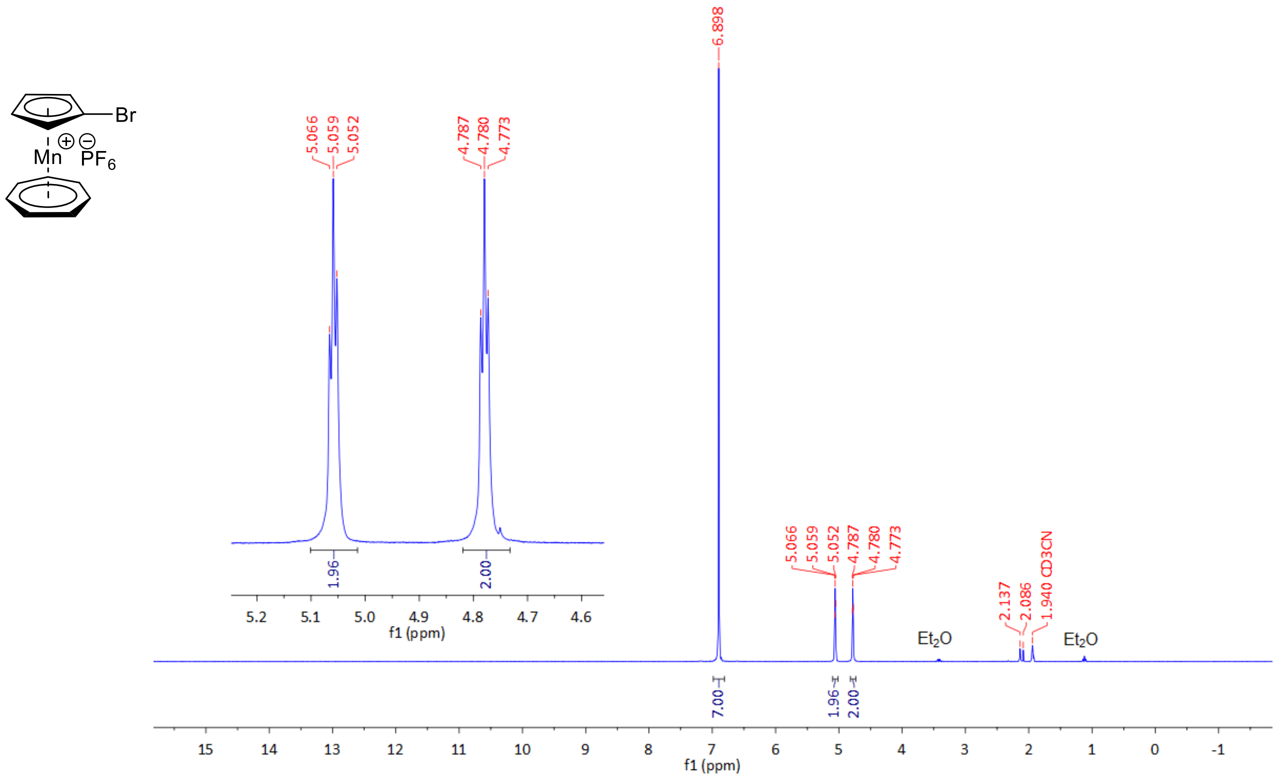

Figure S28. ^{1}H-NMR spectrum of **19**.

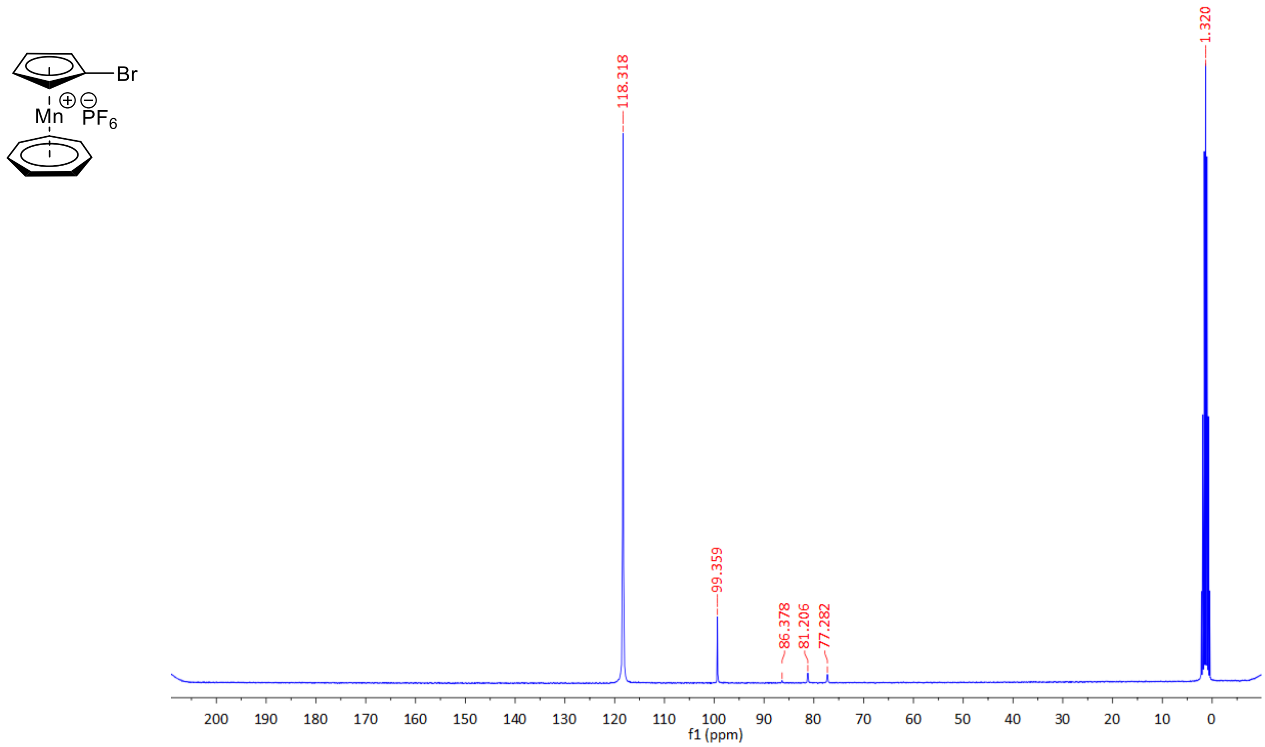

Figure S29. ^{13}C-NMR spectrum of **19**.

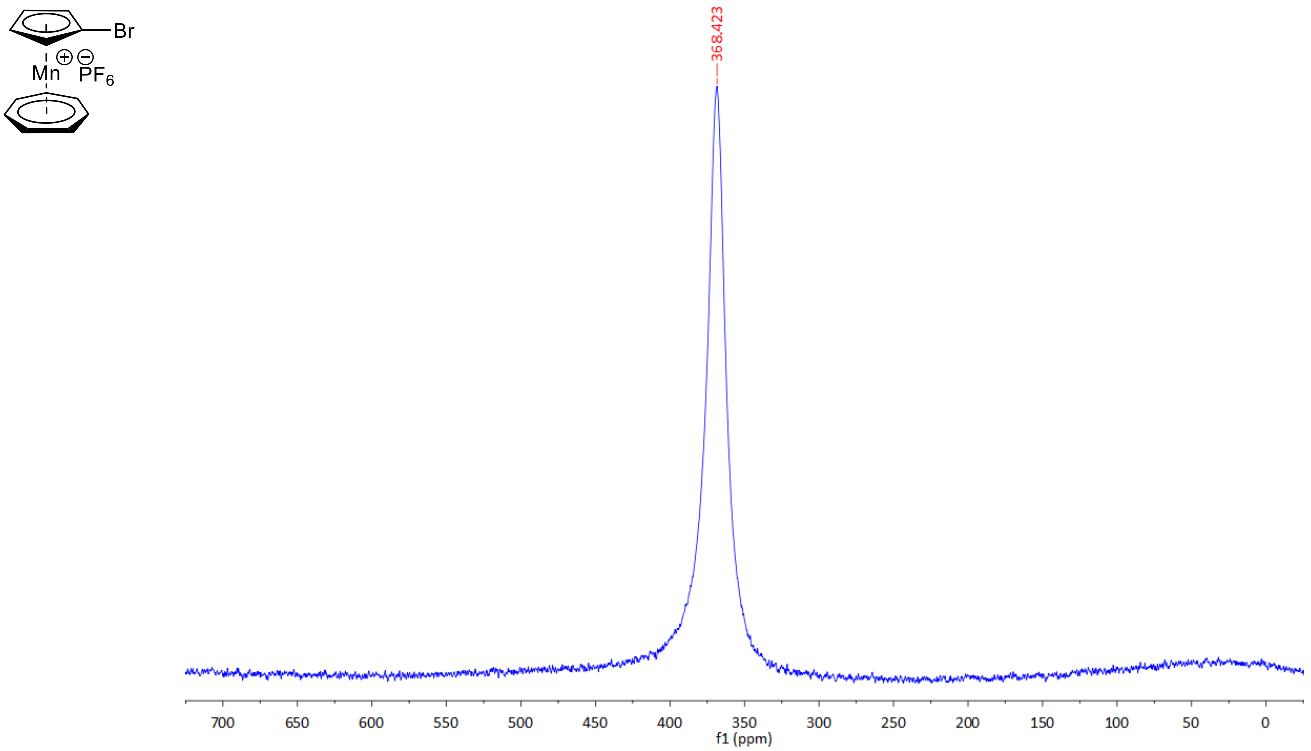

Figure S30. [55]Mn-NMR spectrum of **19**.

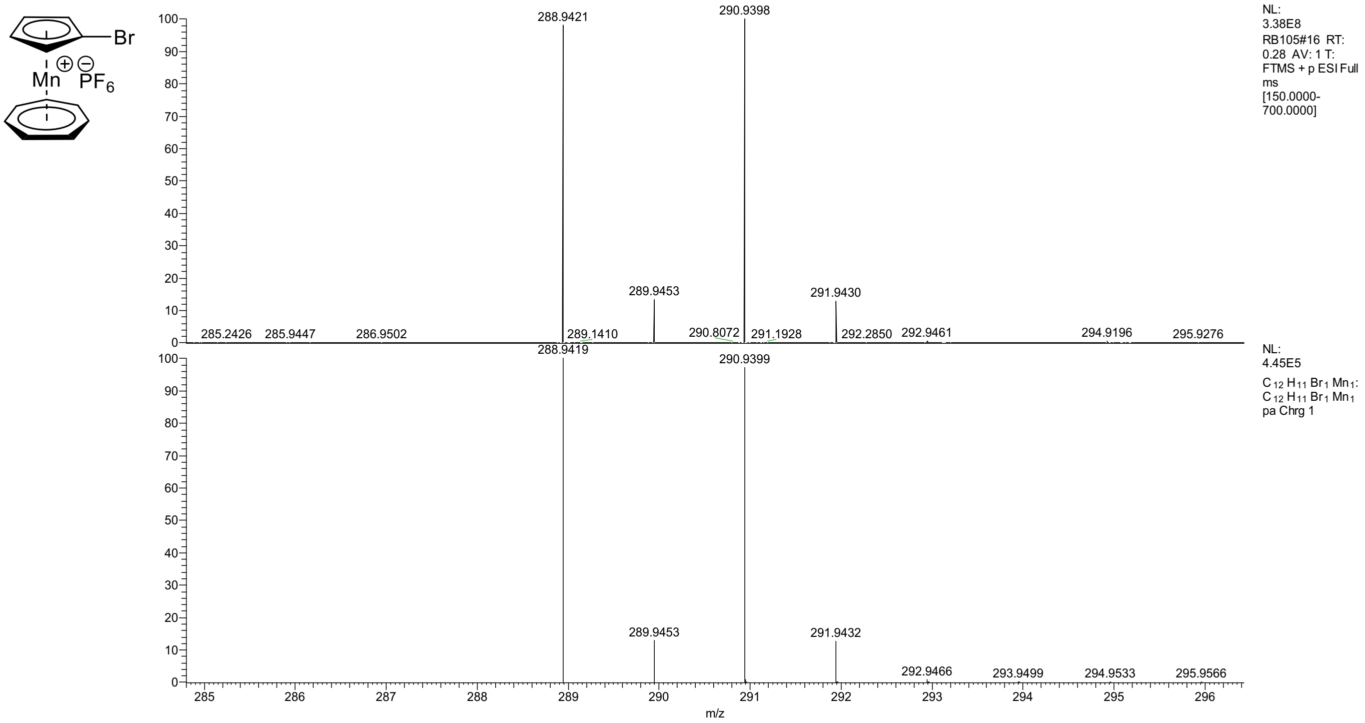

Figure S31. MS (ESI pos, [m/z]; *top*: experimental, *bottom*: simulated) of **19**.

8-Aminotromancenium hexafluoridophosphate (20)

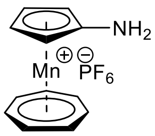

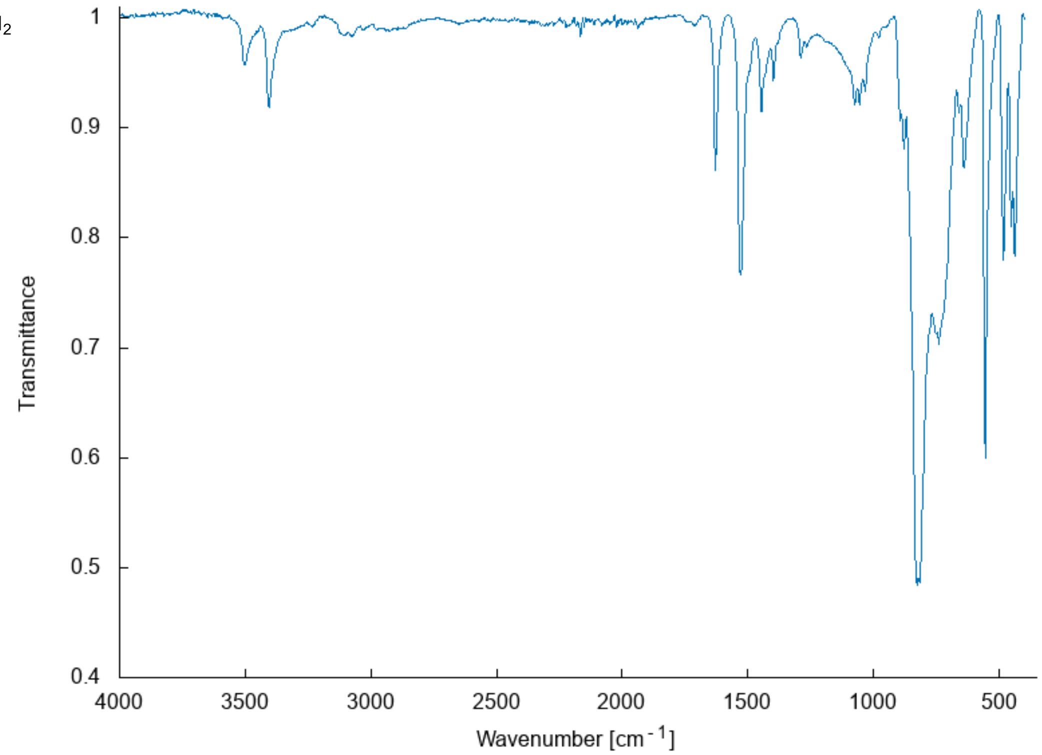

Figure S32. IR spectrum of **20**.

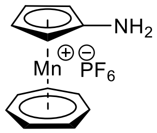

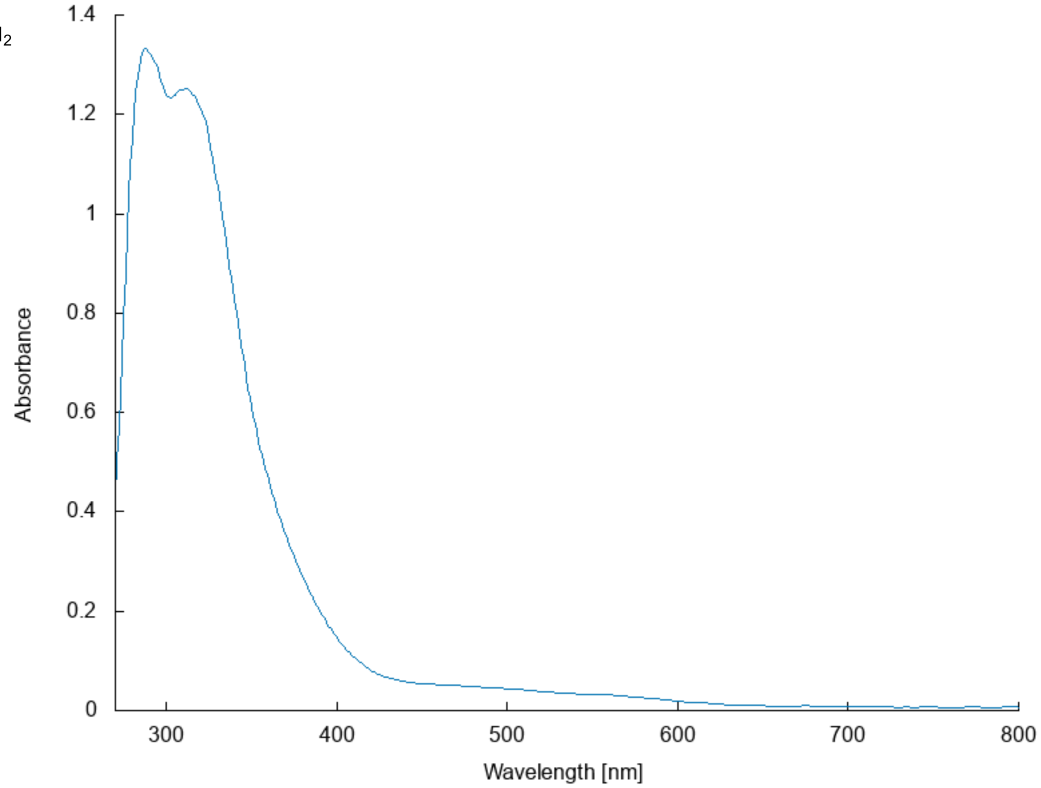

Figure S33. UV/vis spectrum **20**.

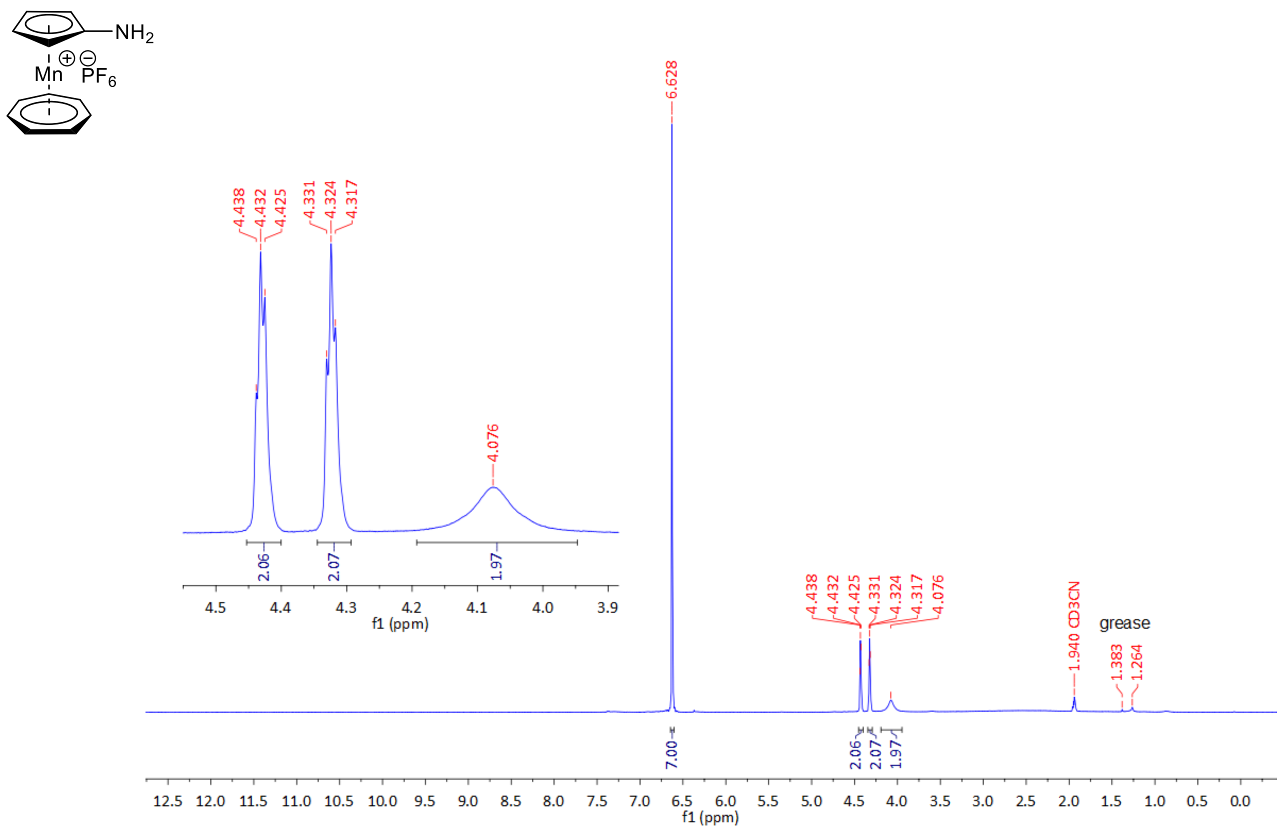

Figure S34. ^{1}H-NMR spectrum of **20**.

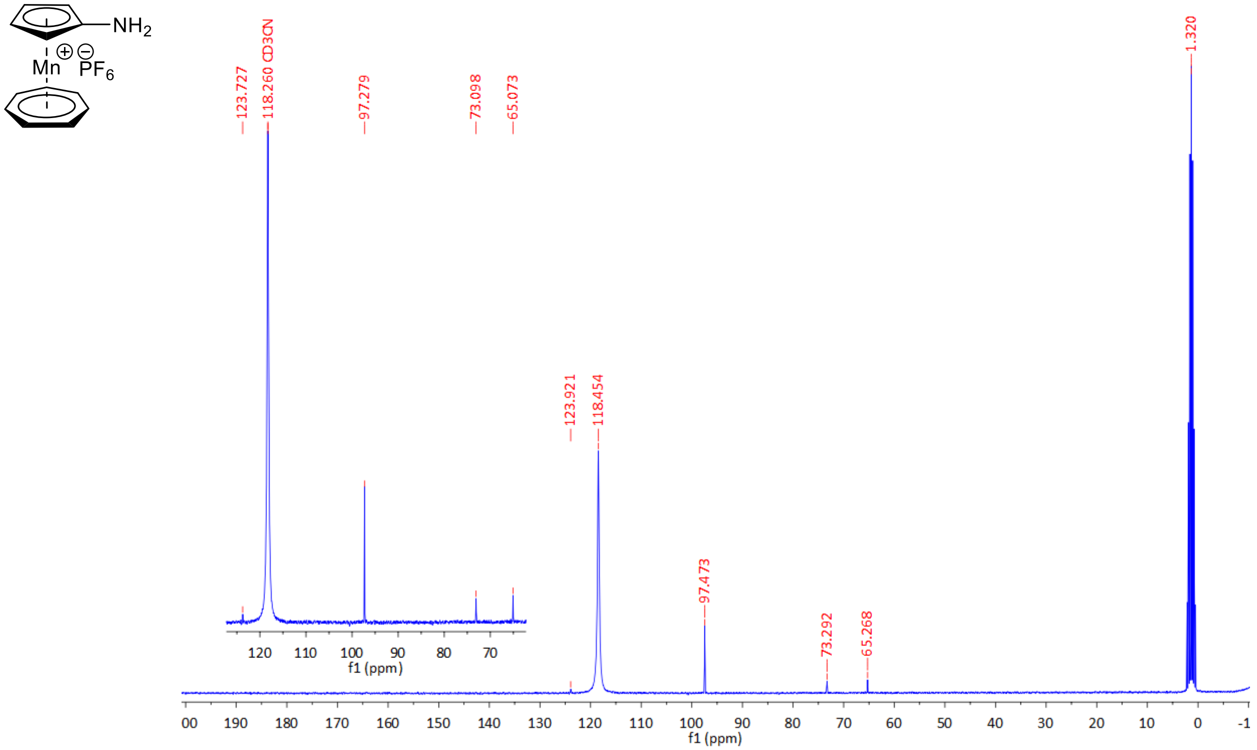

Figure S35. ^{13}C-NMR spectrum of **20**.

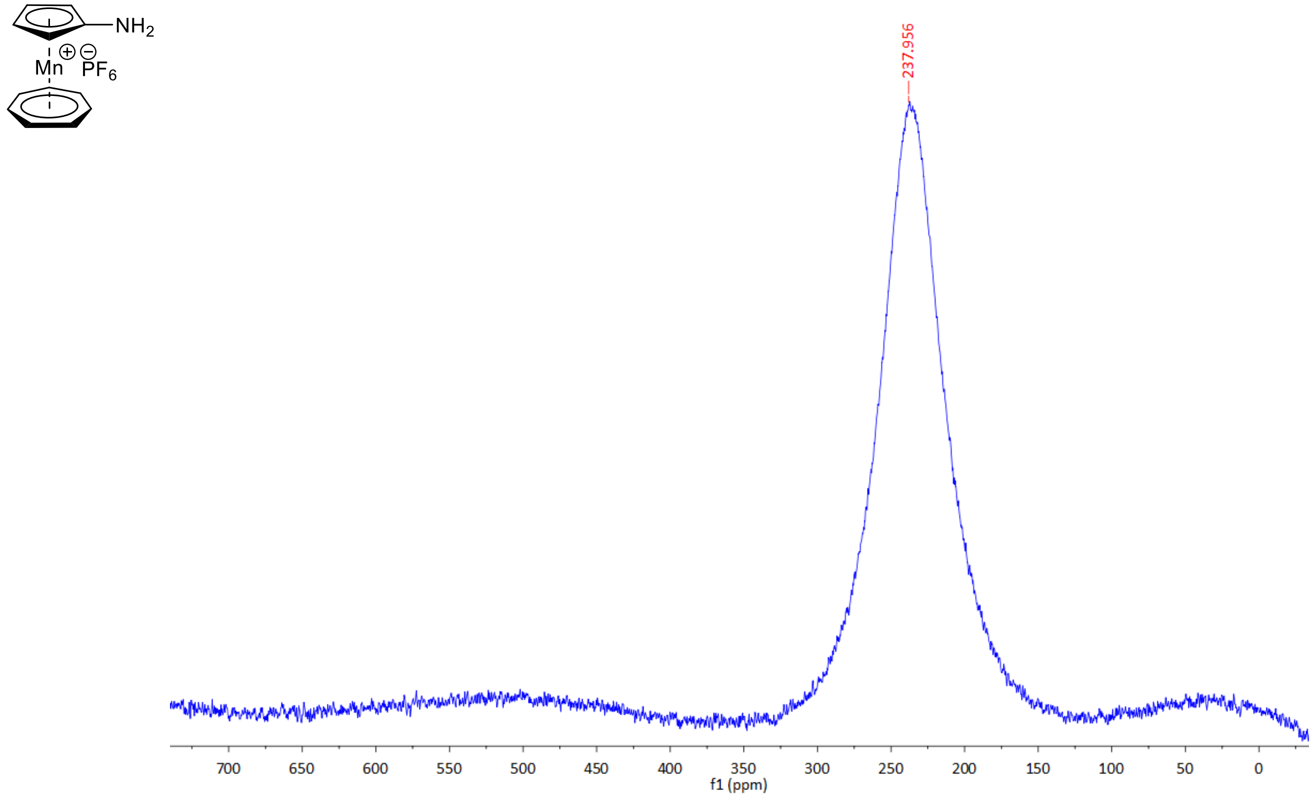

Figure S36. ^{55}Mn-NMR spectrum of **20**.

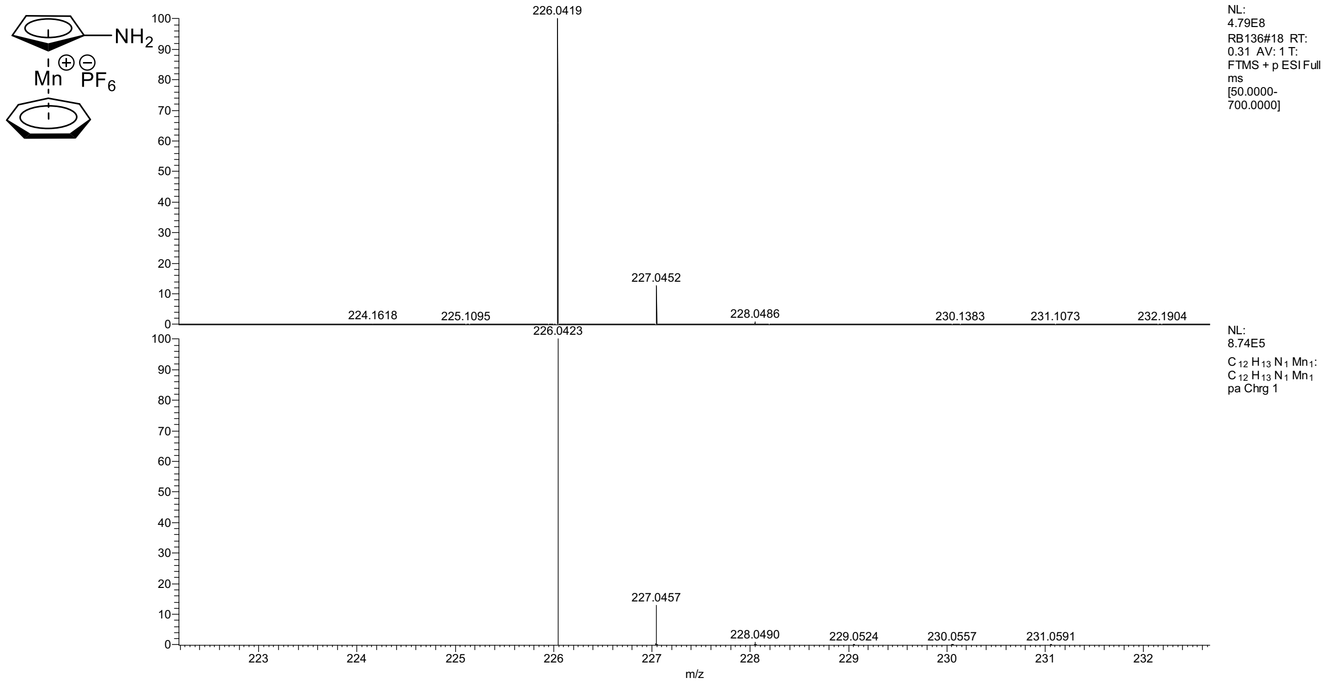

Figure S37. MS (ESI pos, [m/z]; *top*: experimental, *bottom*: simulated) of **20**.

2. XPS measurements

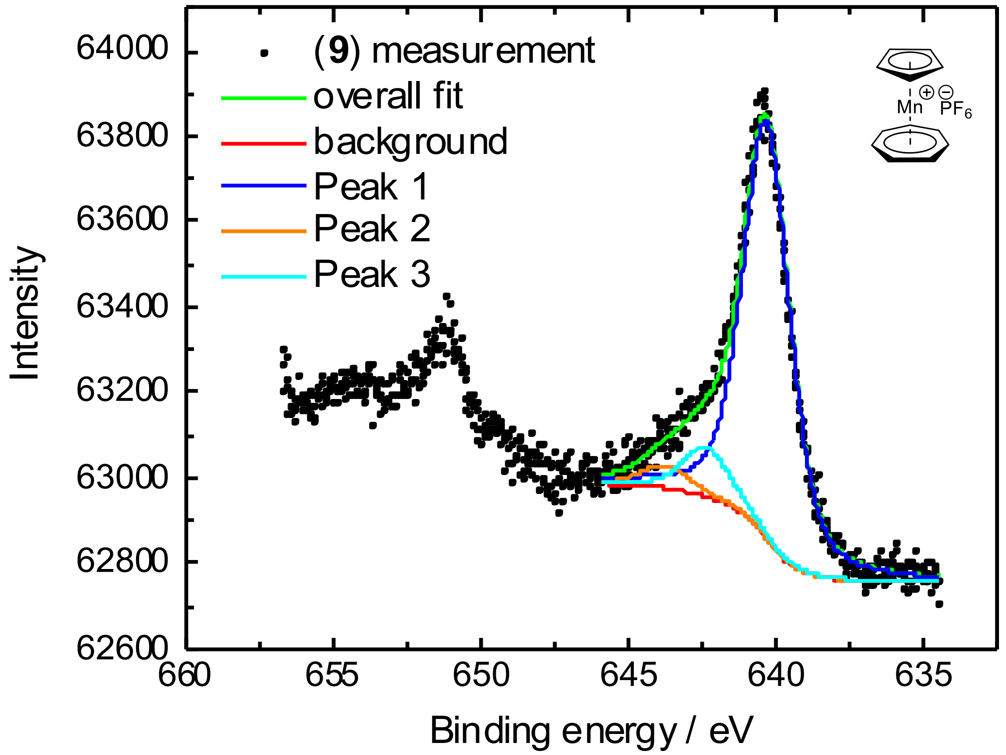

Figure S38. XPS spectrum of **9** (*green:* overall fit, *red:* background, blue*, orange, azure:* split components of the Mn $2p_{3/2}$ region).

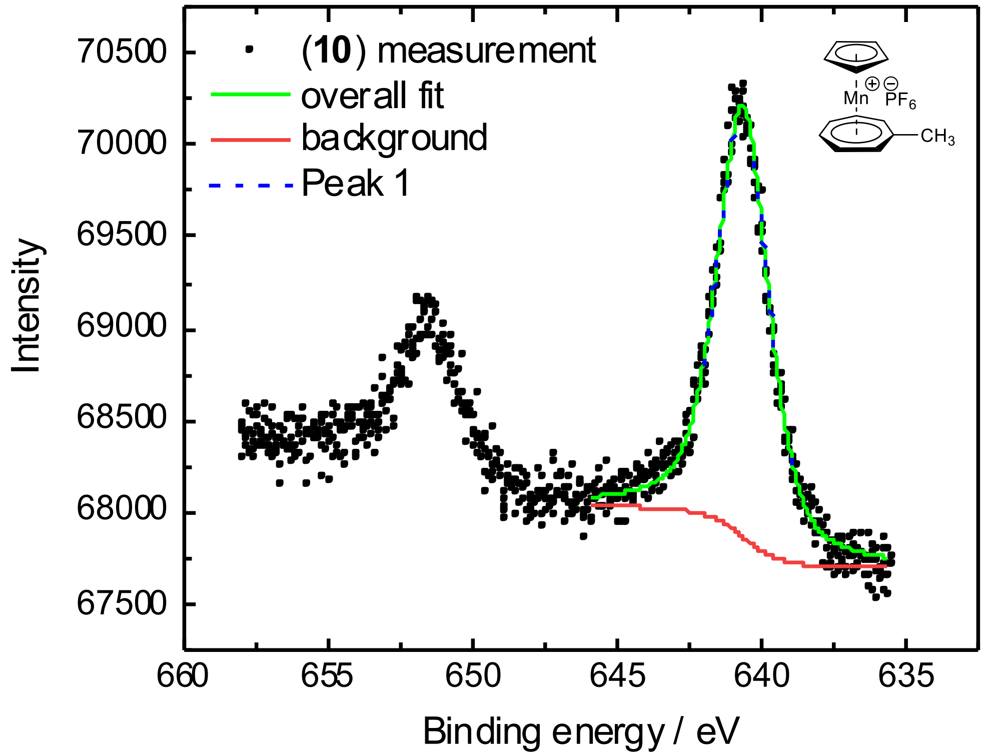

Figure S39. XPS spectrum of **10** (*green:* overall fit, *red:* background, *blue:* split components of the Mn $2p_{3/2}$ region).

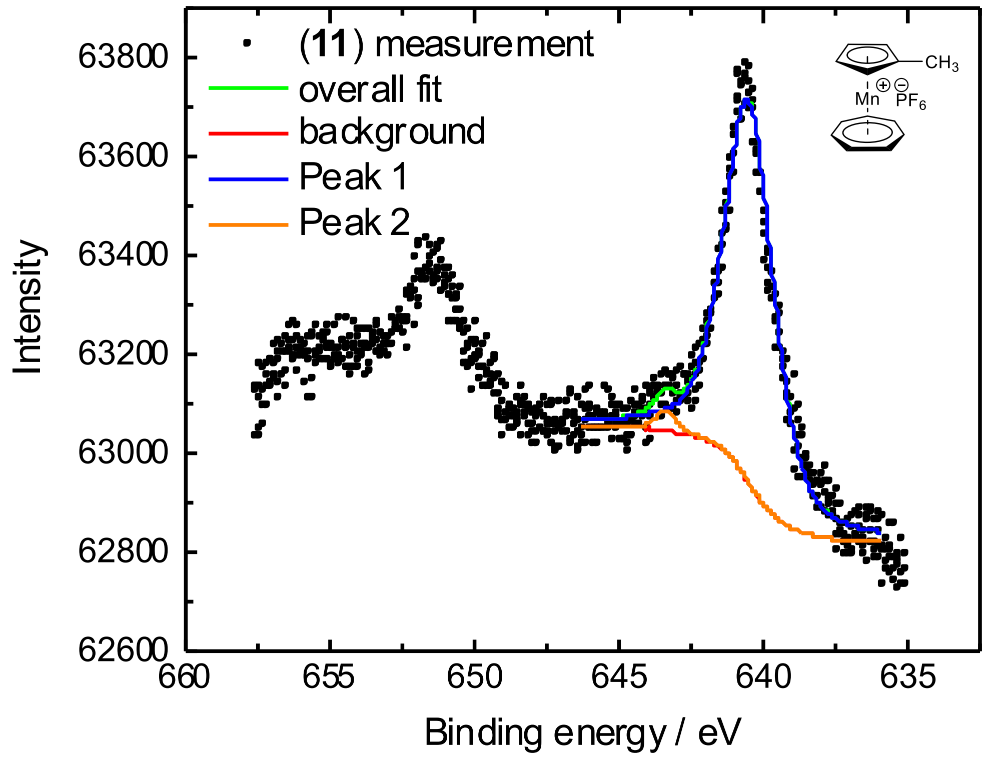

Figure S40. XPS spectrum of **11** (*green:* overall fit, *red:* background, *blue, orange:* split components of the Mn $2p_{3/2}$ region).

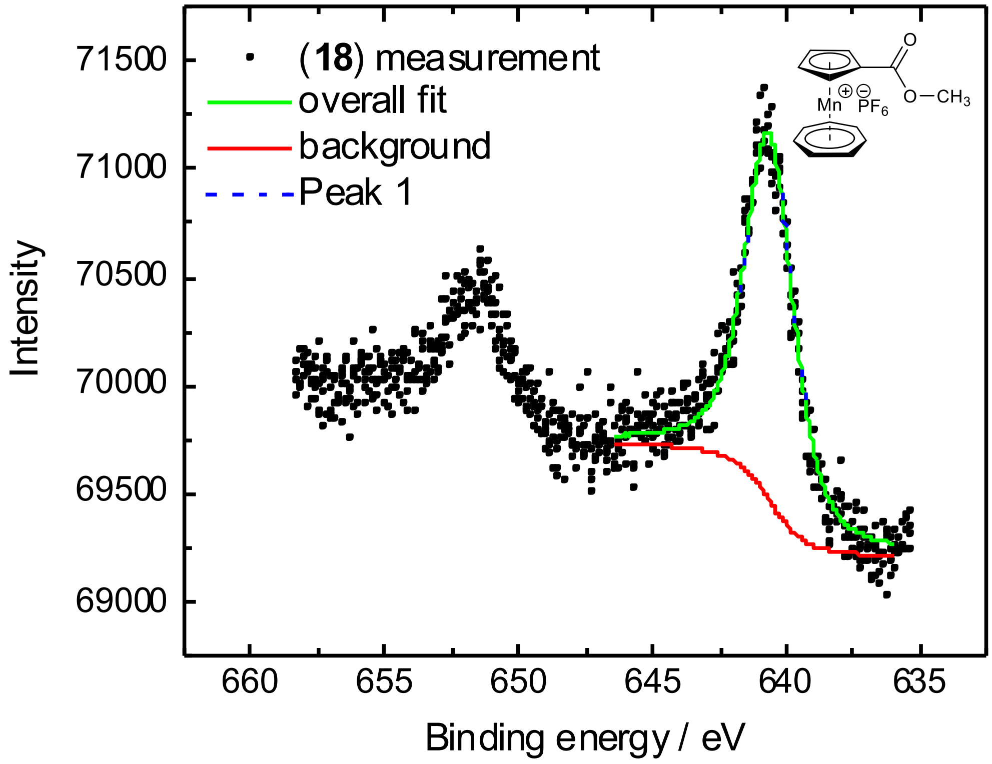

Figure S41. XPS spectrum of **18** (*green:* overall fit, *red:* background, *blue:* split components of the Mn $2p_{3/2}$ region).

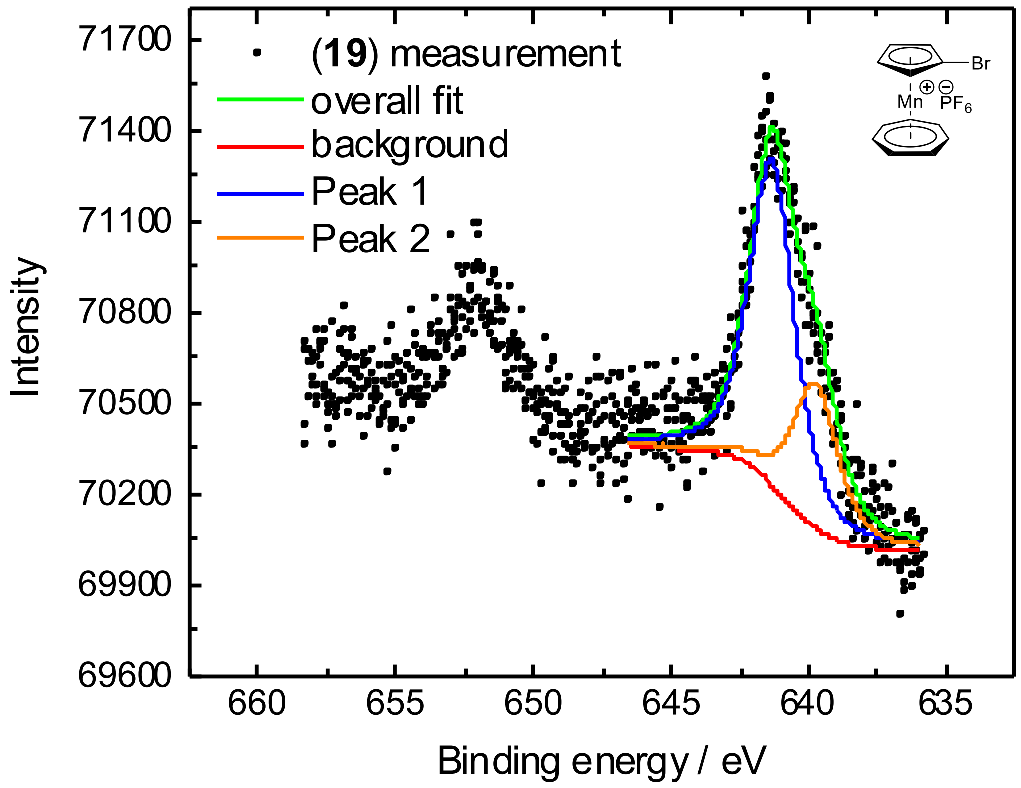

Figure S42. XPS spectrum of **19** (*green:* overall fit, *red:* background, *blue, orange:* split components of the Mn 2p3/2 region).

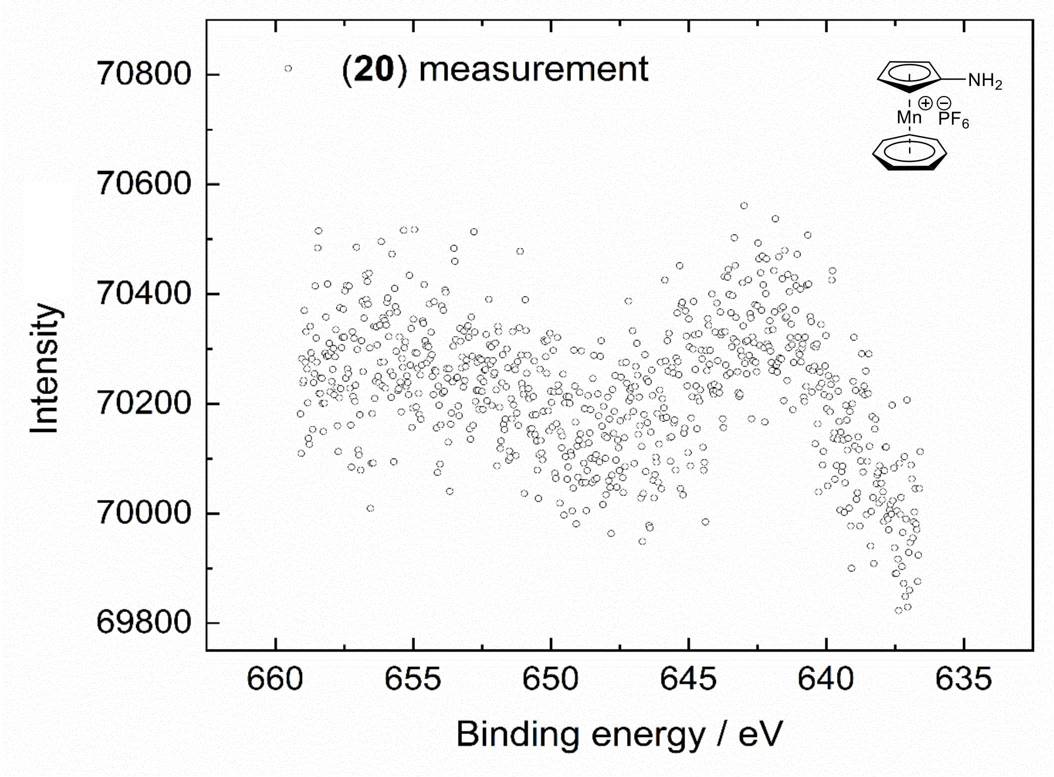

Figure S43. XPS spectrum of **20** (*fitting proved impossible due to degradation of the compound*).

3. Cyclic Voltammetry

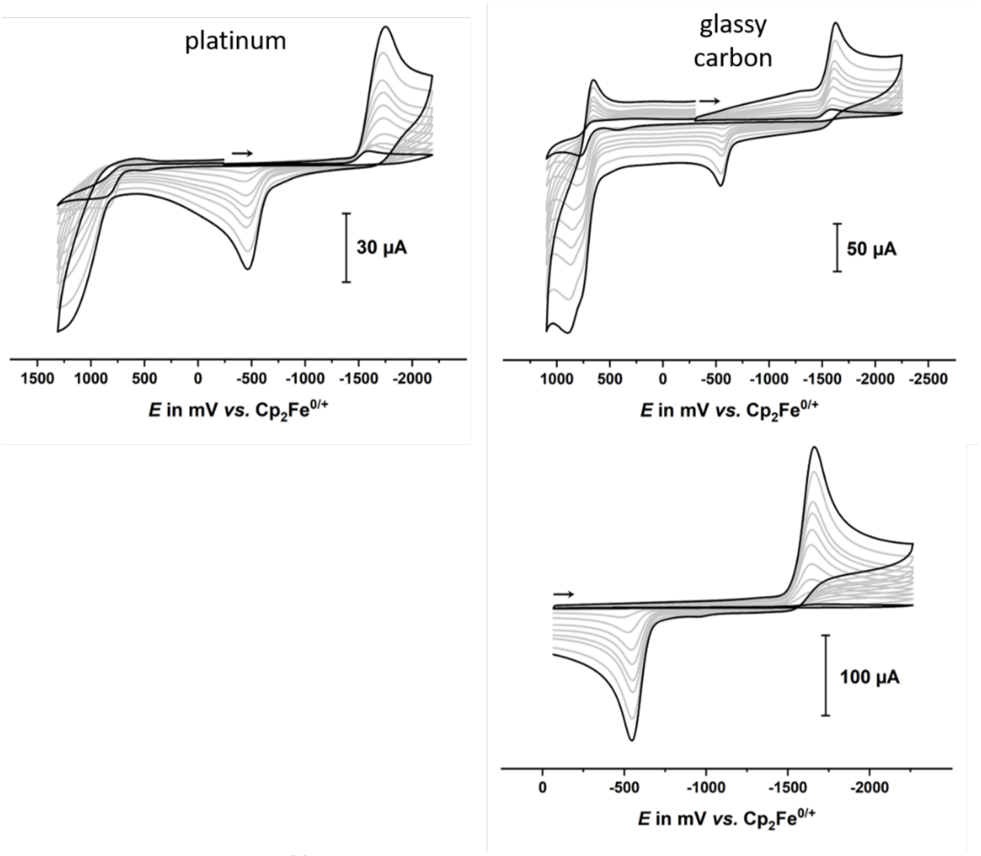

Figure S44. Cyclic voltammogram of [(^{Me}Cht)Mn(Cp)]$^+$ **10** at scan rates from 25 mV/s to 2000 mV/s in DMF/0.1 M NBu_4^+ PF_6^- with a platinum (left) and a glassy carbon electrode (right).

Tromancenium ion 9 [(Cht)Mn(Cp)]$^+$**:**

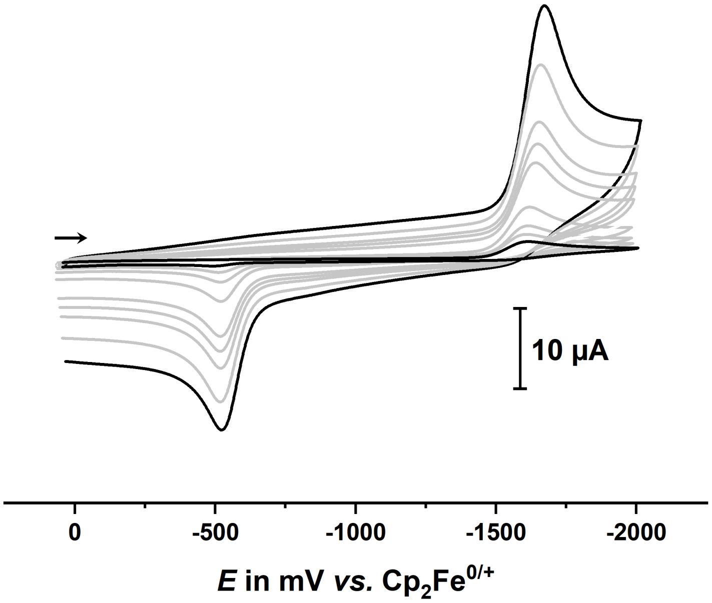

Figure S45. Cyclic voltammogram of **9** at different scan rates from 25 to 2000 mV/s in DMF/0.1 M NBu_4^+ PF_6^- with a platinum electrode.

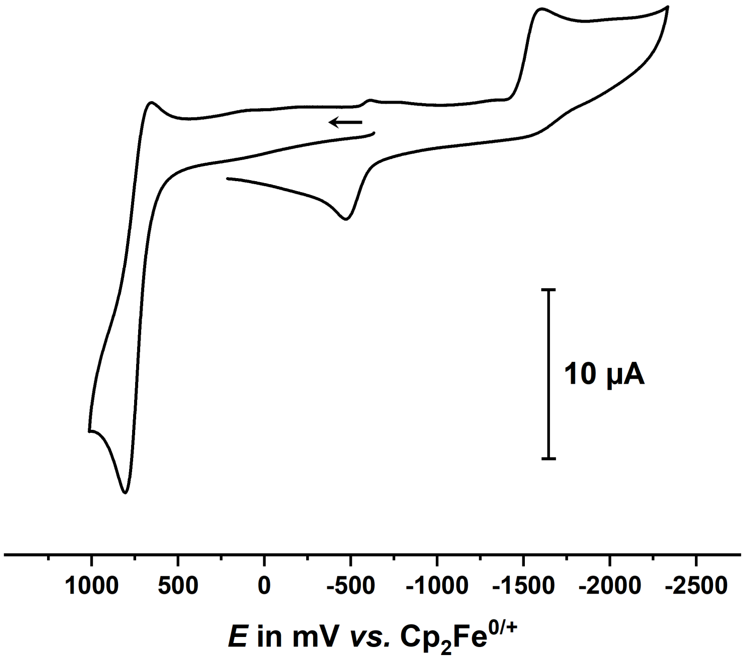

Figure S46. Cyclic voltammogram of **9** at a scan rate of 100 mV/s in DMF/0.1 M NBu_4^+ PF_6^- with a platinum electrode; the wave at -495 mV occurs only after prior reduction.

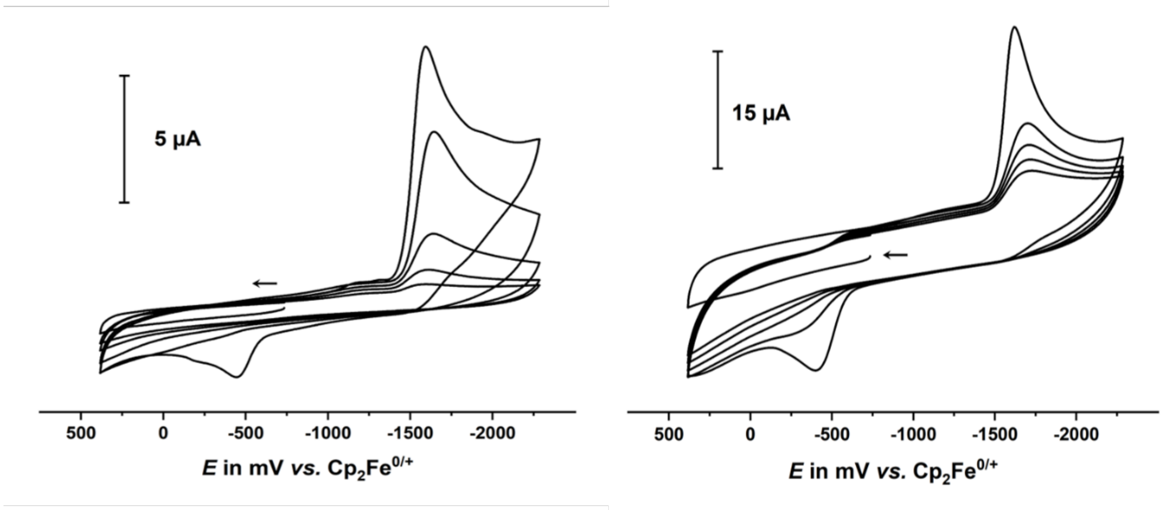

Figure S47. Cyclic voltammogram of **9** at a scan rate of 100 mV/s (left) and 600 mV/s (right) for multiple cycles in DMF/0.1 M NBu_4^+ PF_6^- with a platinum electrode, demonstrating the rapid electrode fouling.

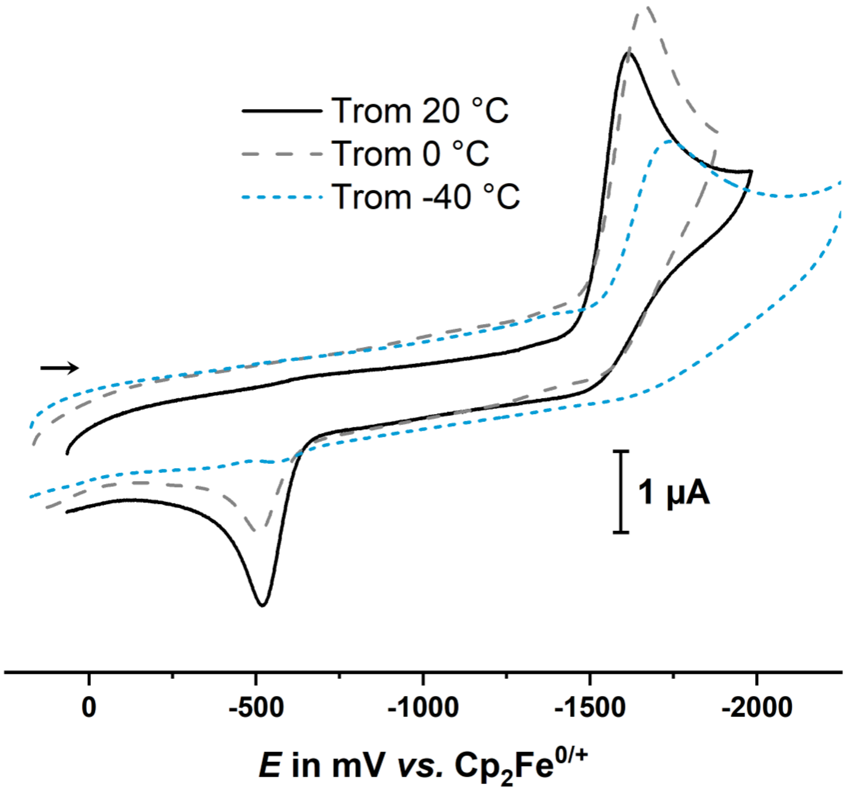

Figure S48. Cyclic voltammogram of **9** at different temperatures at a scan rate of 100 mV/s in DMF/0.1 M NBu_4^+ PF_6^- with a platinum electrode.

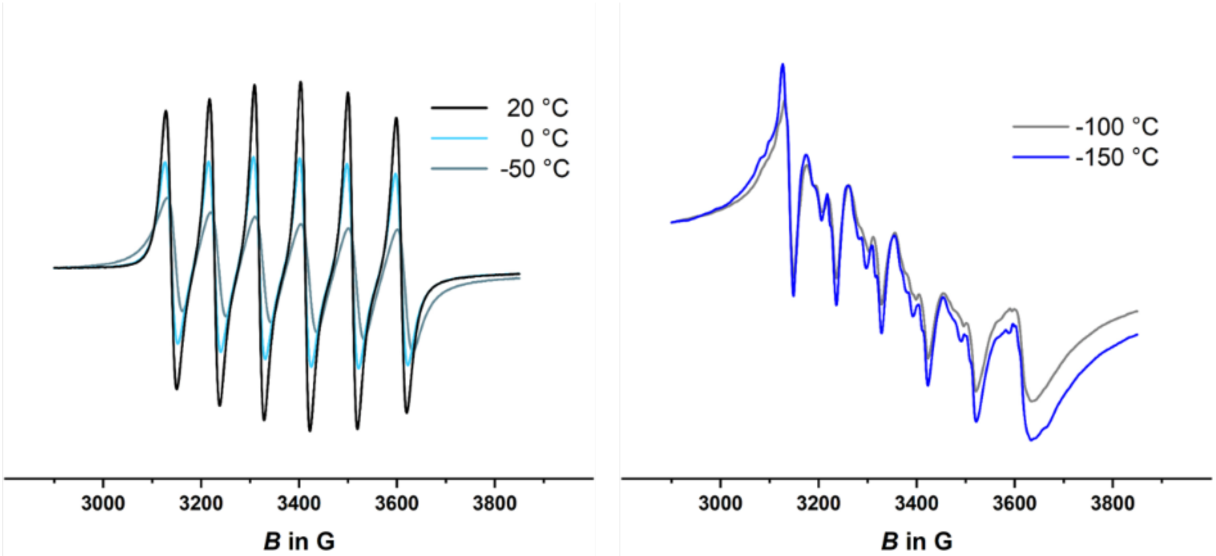

Figure S49. *T*-dependent EPR spectra of chemically reduced **9** in DMF; reducing agent: decamethylcobaltocene.

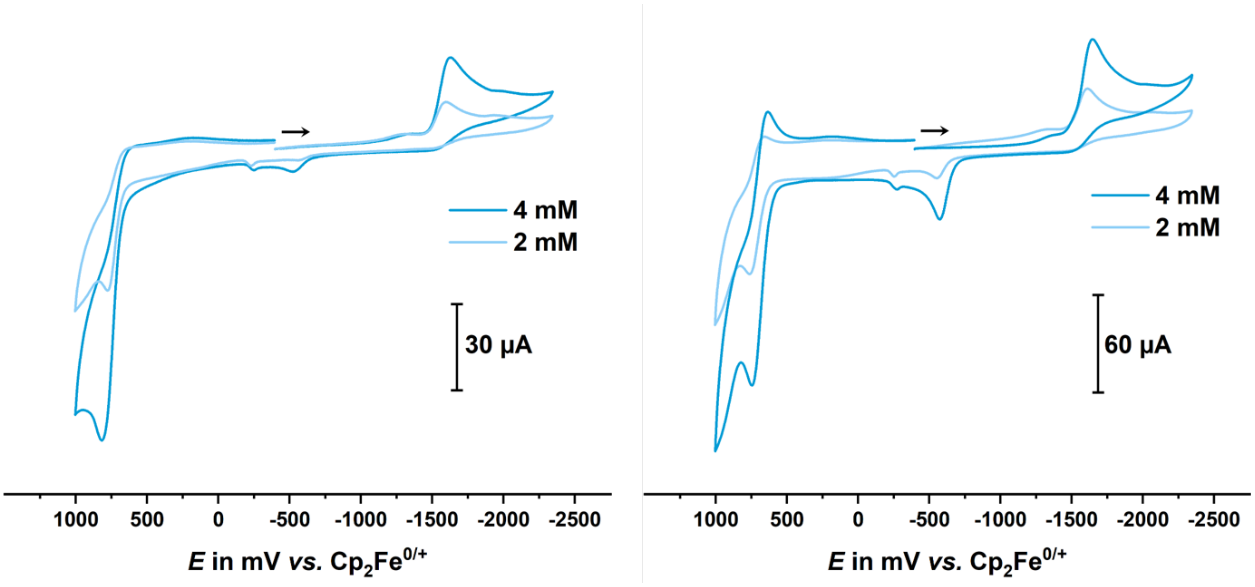

Figure S50. Cyclic voltammogram of **9** in two different concentrations at a scan rate of 100 mV/s (left) and 600 mV/s (right) in DMF/0.1 M NBu_4^+ PF_6^- with a glassy carbon electrode.

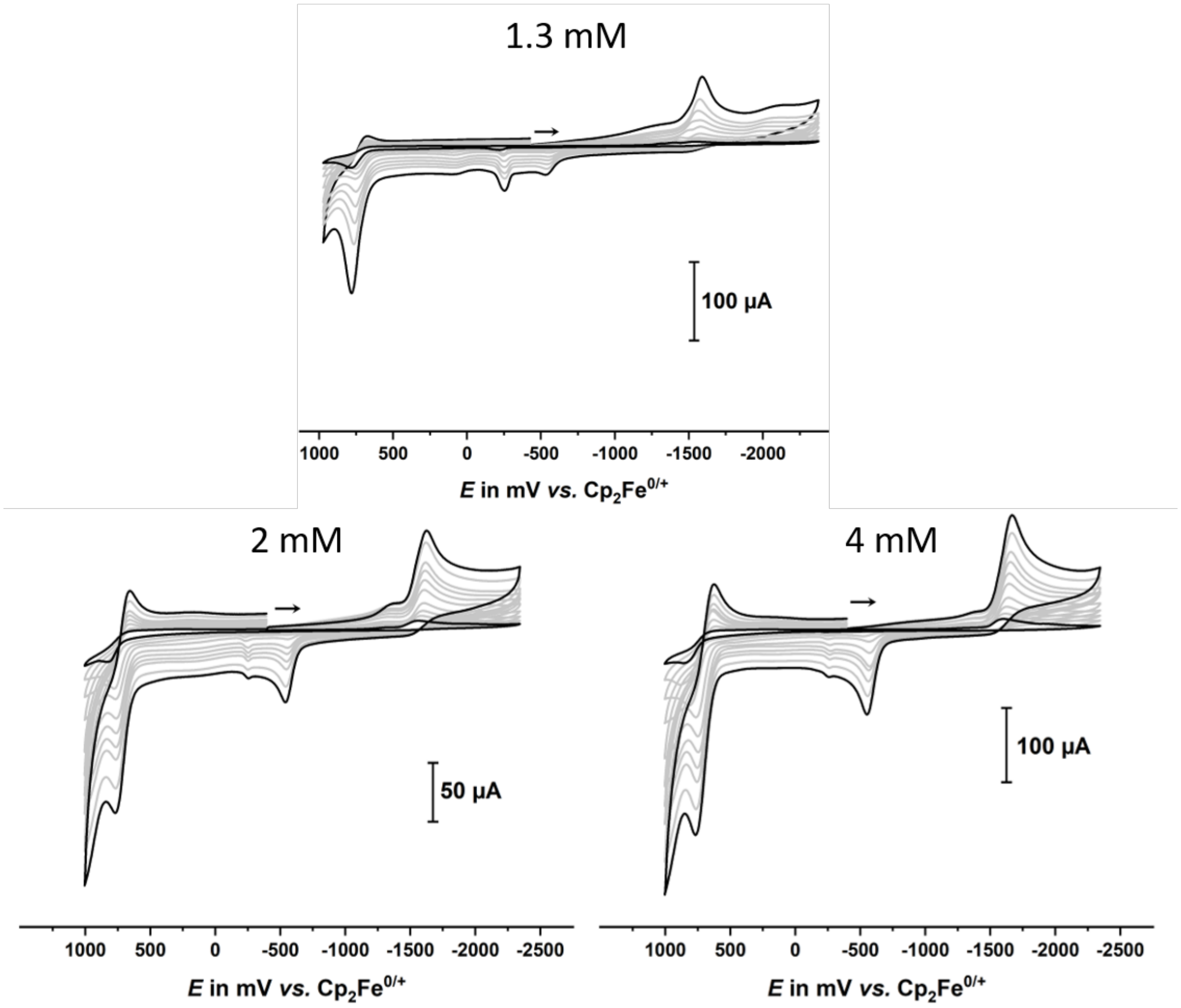

Figure S51. Cyclic voltammogram of **9** in three different concentrations (top: 1.3 mM; bottom, left: 2 mM; right: 4 mM) at scan rates from 25 mV/s to 2000 mV/s in DMF/0.1 M NBu_4^+ PF_6^- with a glassy carbon electrode.

Tromancenium ion 10 $[(^{Me}Cht)Mn(Cp)]^{+}$**:**

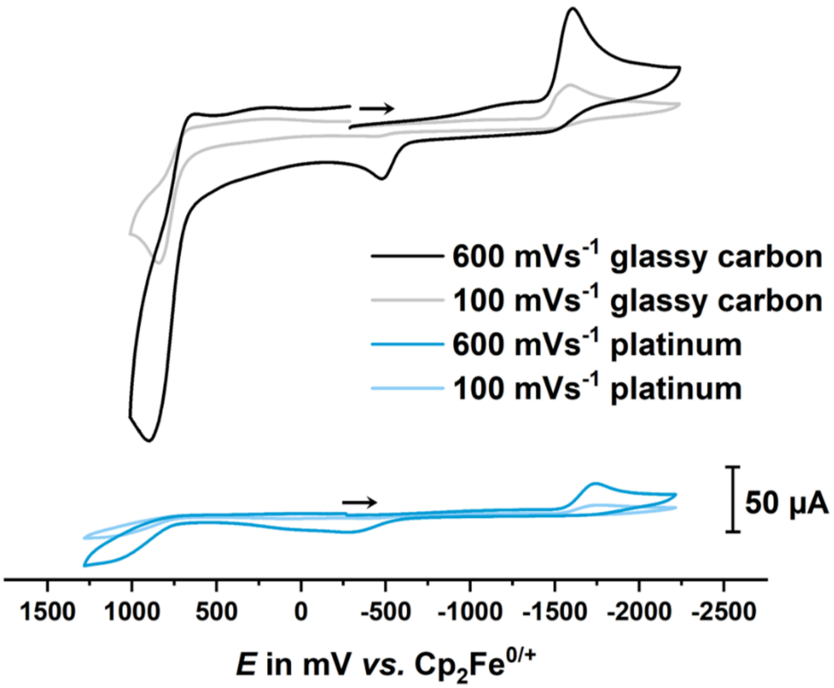

Figure S52. Cyclic voltammograms of **10** (c = 4 mM) at scan rates of 100 mV/s to 600 mV/s in DMF/0.1 M NBu_4^+ PF_6^- with a platinum (blue) and a glassy carbon electrode (black,grey).

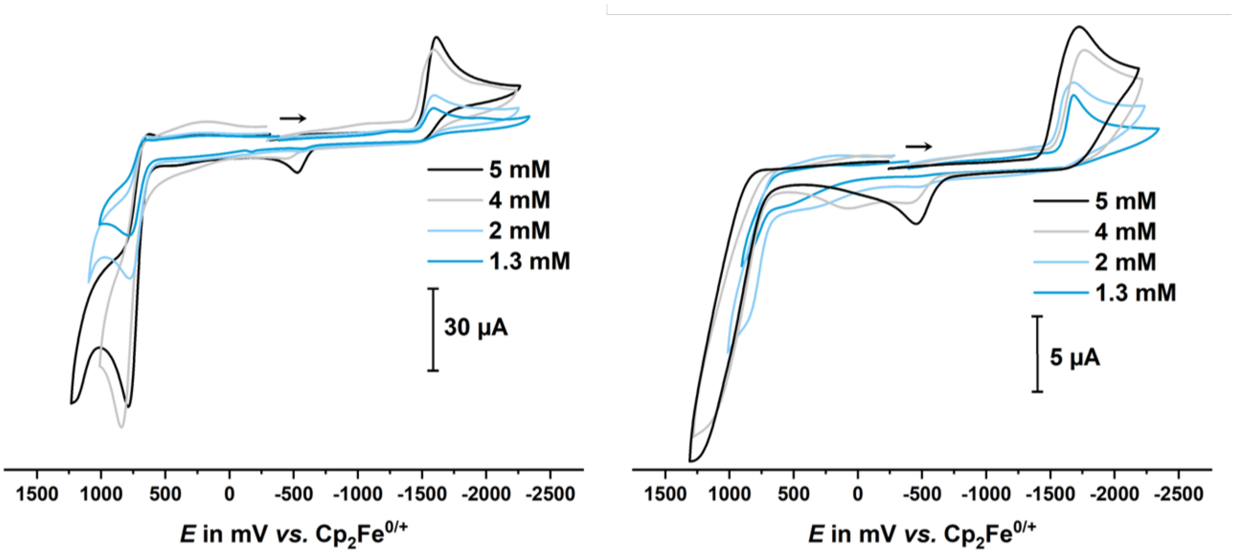

Figure S53. Cyclic voltammograms of **10** in four different concentrations at a scan rates of 100 mV/s in DMF/0.1 M NBu_4^+ PF_6^- with a platinum (right) and a glassy carbon electrode (left).

Tromancenium ion 11 [(Cht)Mn(Cp^{Me})]$^+$**:**

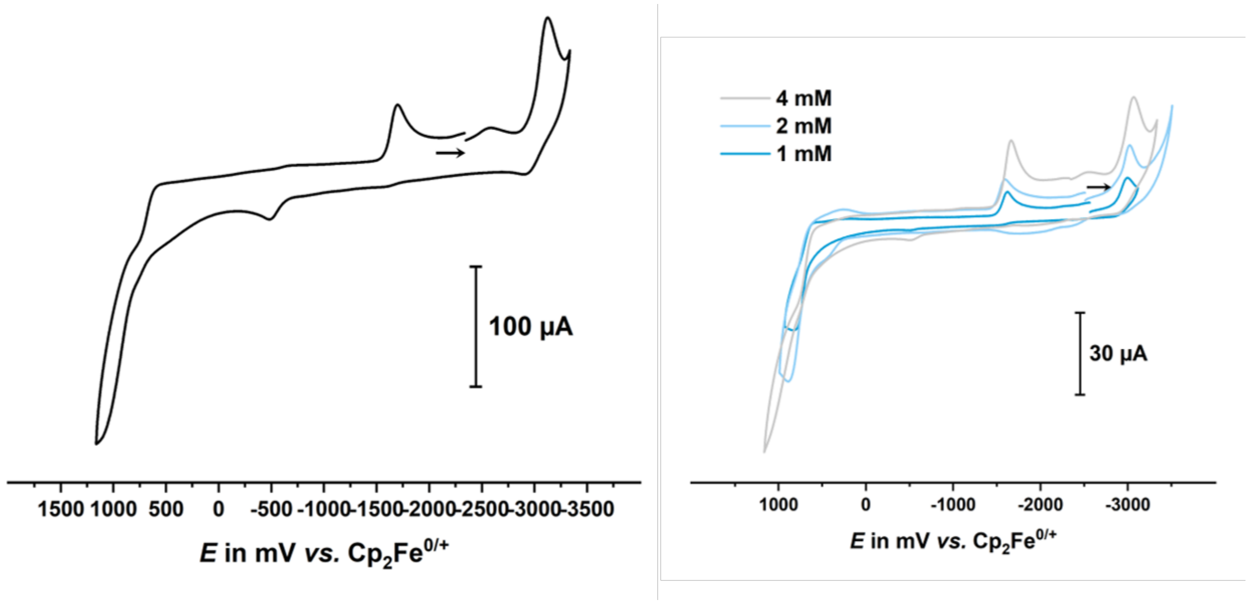

Figure S54. Cyclic voltammograms of **11** in DMF/0.1 M NBu_4^+ PF_6^- with a glassy carbon electrode; left: full range at a scan rate of 100 mV/s; right: scan rate of 100 mV/s for three different concentrations.

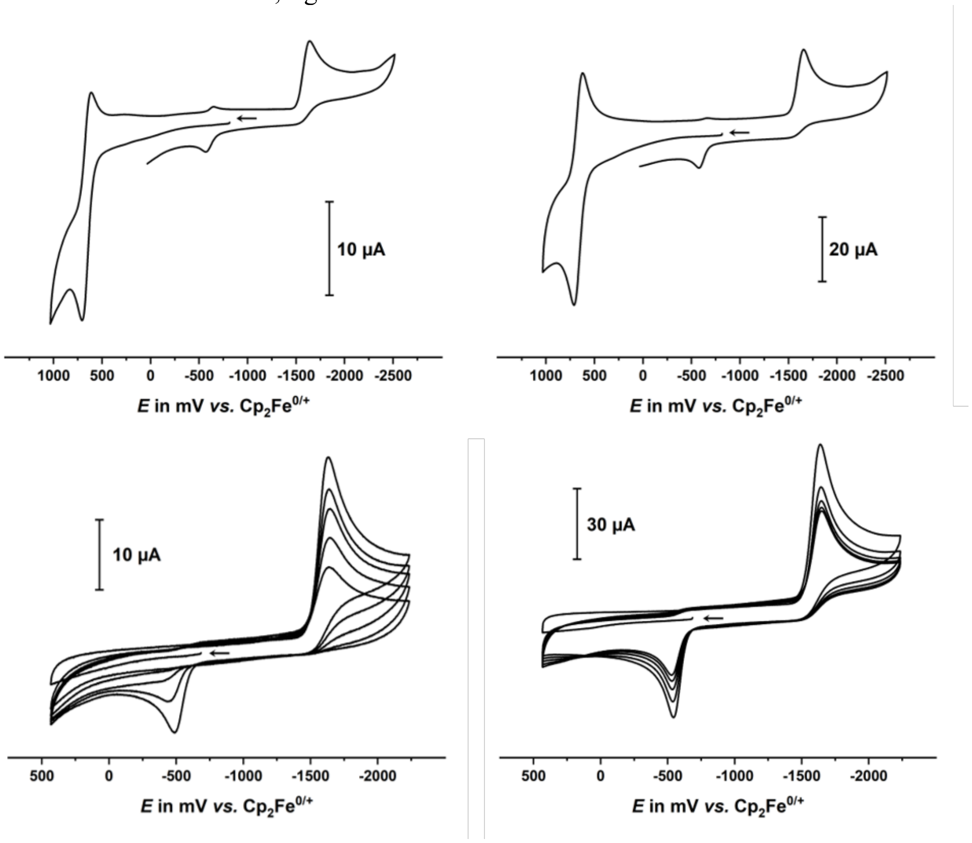

Figure S55. Cyclic voltammograms of **11** in DMF/0.1 M NBu_4^+ PF_6^- with a glassy carbon electrode, scan rates of 100 mV/s (left) and 600 mV/s (right), multiple scans (bottom).

Tromancenium ion 18 [(Cht)Mn(Cp^{COOMe})]$^+$**:**

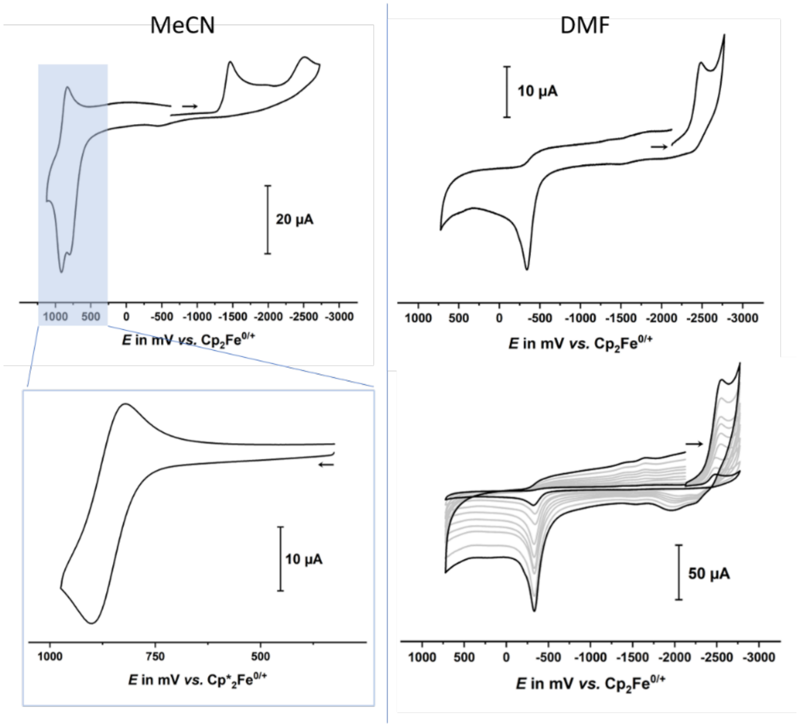

Figure S56. Cyclic voltammograms of **18** in MeCN/0.1 M NBu_4^+ PF_6^- (left) and DMF/0.1 M NBu_4^+ PF_6^- (right) with a glassy carbon electrode; left: full range (top) and oxidation only (bottom) at a scan rate of 100 mV/s; right: scan rate of 100 mV/s (top) and different scan rates from 25 to 2000 mV/s (bottom), oxidation not accessible in the potential range of DMF.

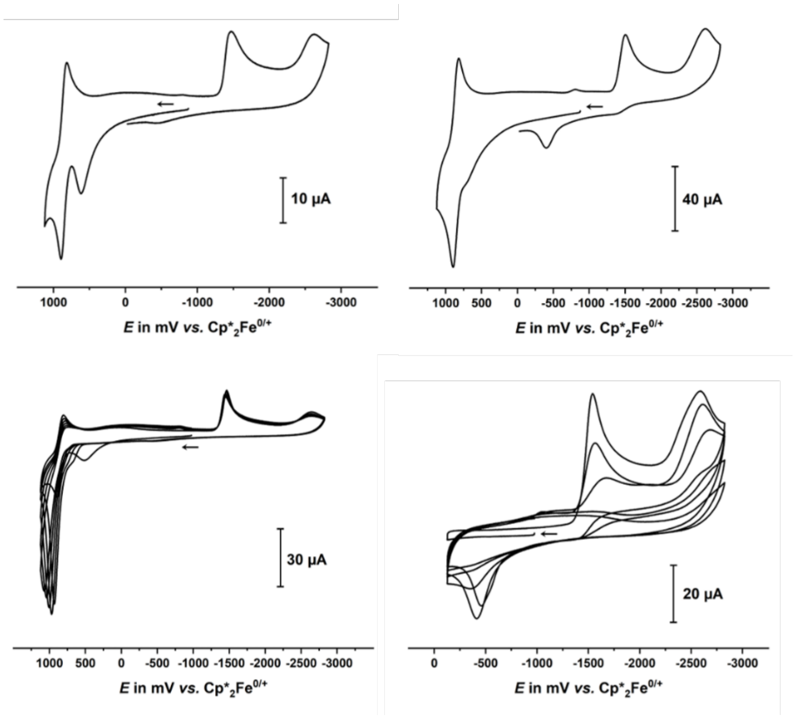

Figure S57. Cyclic voltammograms of **18** in MeCN/0.1 M NBu_4^+ PF_6^- with a glassy carbon electrode, scan rates of 100 mV/s (left) and 600 mV/s (right), multiple scans (bottom), demonstrating rapid electrode passivation.

Tromancenium ion 19 [(Cht)Mn(Cp^{Br})]$^+$**:**

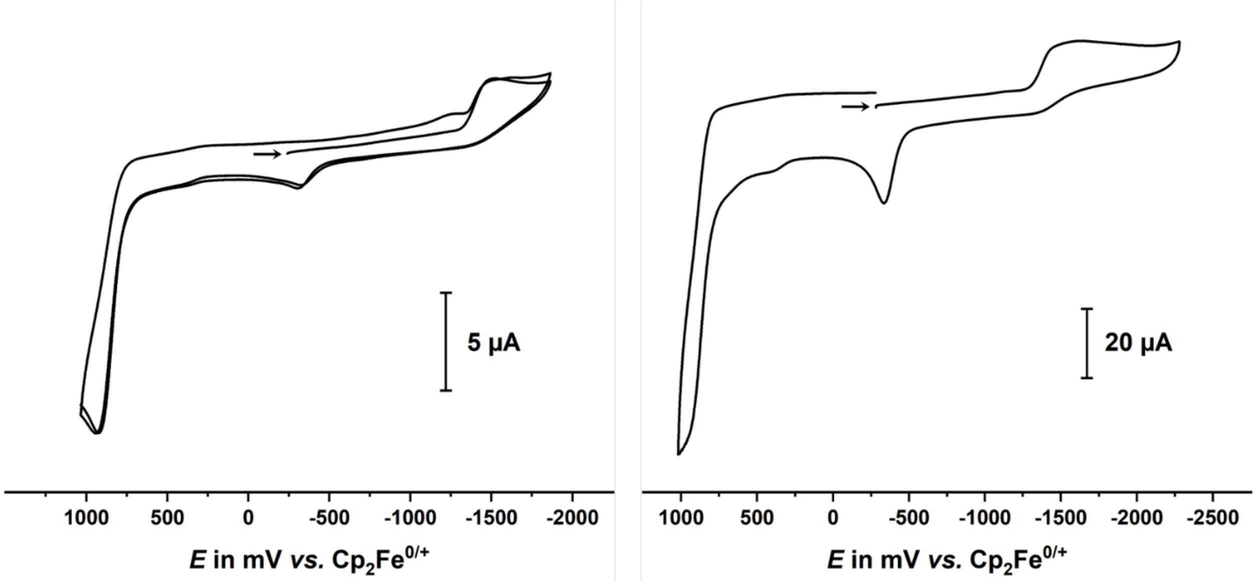

Figure S58. Cyclic voltammograms of **19** in DMF/0.1 M NBu_4^+ PF_6^- with a glassy carbon electrode, scan rates of 100 mV/s (left) and 600 mV/s (right).

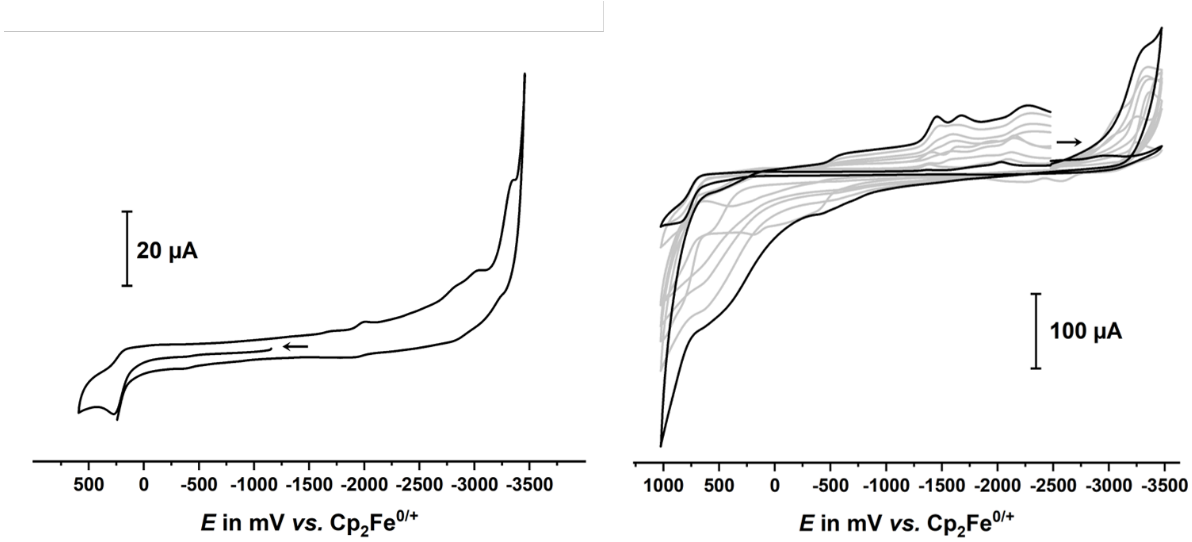

Figure S59. Cyclic voltammograms of **19** in DMF/0.1 M NBu_4^+ PF_6^- with a glassy carbon electrode at a scan rates of 100 mV/s, the oxidation leads to instantaneous passivation of the electrode (left); at different scan rates from 50 mV/s to 2000 mV/s, several small cathodic peaks occur on the reverse scan (right).

Tromancenium ion 20 [(Cht)Mn(Cp^{NH2})]$^+$**:**

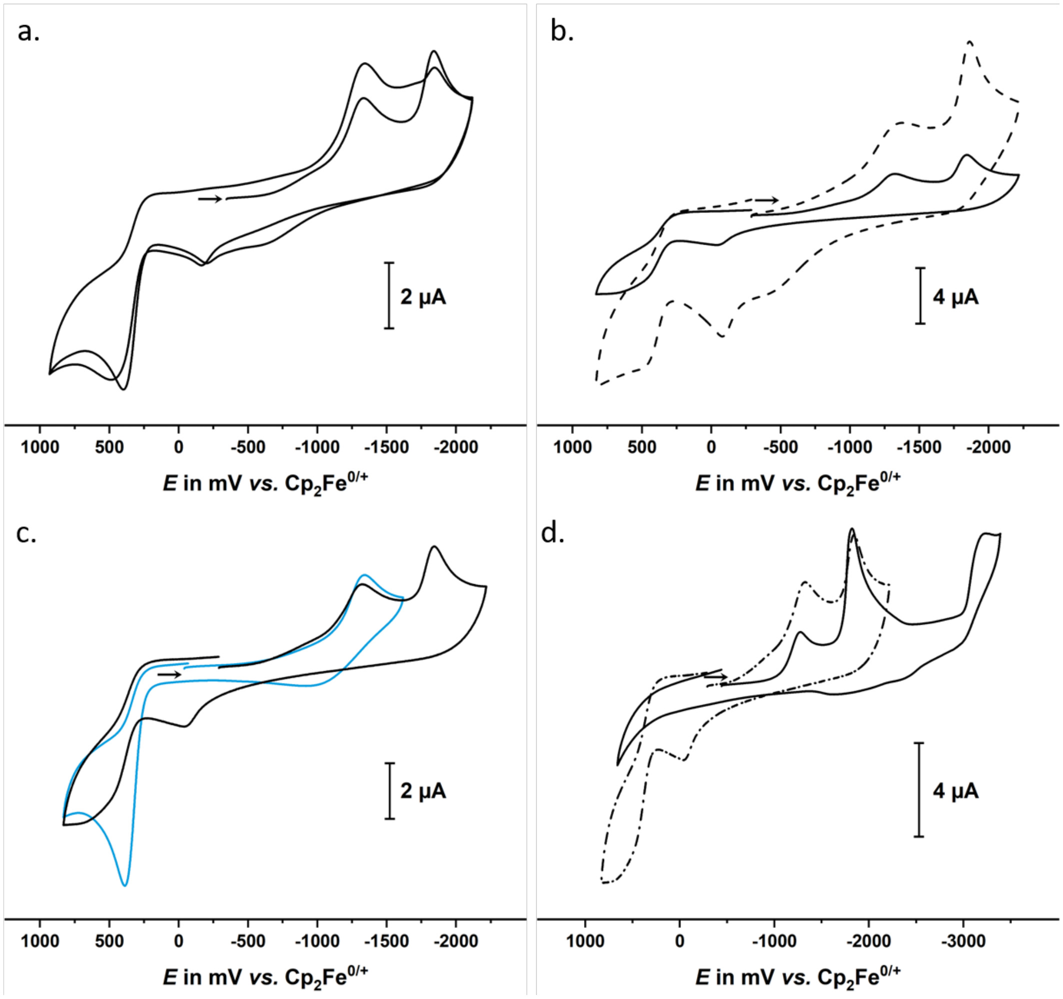

Figure S60. Cyclic voltammogram of **20** at a scan rate of 100 mV/s (a-d) and 600 mV/s (b, dotted) in DMF/0.1 M NBu_4^+ PF_6^- with a platinum electrode; a. multiple scans, b. two different scan rates, c. and d. different ranges.

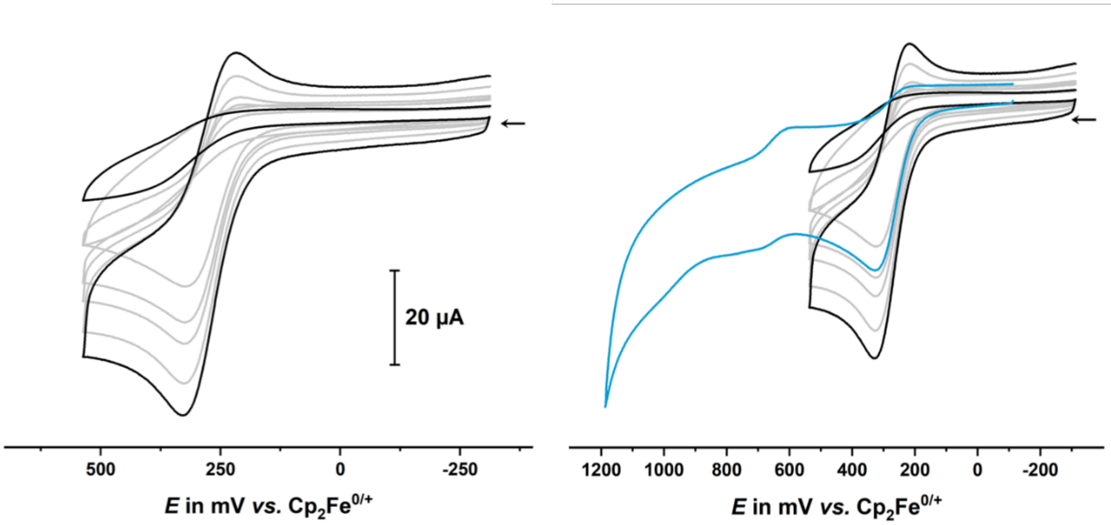

Figure S61. Cyclic voltammogram of **20** focusing on the oxidation at different scan rates from 100 to 2000 mV/s in DMF/0.1 M NBu_4^+ PF_6^- with a glassy carbon electrode with one scan (right, blue) further in anodic direction.

4. Density Functional Theory Calculations

Structural parameters of **9** and its reduced from **[9]**$^-$ calculated with DFT in gas phase and in implicitly modelled DMSO were listed in Tables S1 and S2.

Table S1. Structural parameters of **9**. Bulk solvent effects were modelled implicitly. For the PBE0-D3 and the BP86-D3 density functional, structures were fully optimized with the def2-TZVPP basis set, whereas for the ωB97x-d functional, structures were optimized with the def2-SVP basis set.

	PBE0-D3		BP86-D3		ωB97x-d	
	Gas	DMSO	Gas	DMSO	Gas	DMSO
Mn-Cp	1.735	1.727	1.742	1.734	1.739	1.745
Mn-Cht	1.391	1.384	1.413	1.401	1.393	1.389
Mn-C1	2.112	2.107	2.123	2.117	2.117	2.123
Mn-C2	2.112	2.107	2.124	2.117	2.117	2.124
Mn-C3	2.112	2.107	2.124	2.117	2.118	2.123
Mn-C4	2.112	2.107	2.124	2.117	2.118	2.123
Mn-C5	2.112	2.107	2.124	2.117	2.118	2.124
Mn-C6	2.128	2.123	2.152	2.145	2.135	2.134
Mn-C7	2.130	2.126	2.155	2.148	2.137	2.137
Mn-C8	2.132	2.128	2.156	2.150	2.138	2.136
Mn-C9	2.129	2.124	2.152	2.146	2.136	2.133
Mn-C10	2.129	2.124	2.152	2.146	2.135	2.135
Mn-C11	2.132	2.128	2.156	2.150	2.138	2.137
Mn-C12	2.130	2.126	2.155	2.148	2.138	2.135
C1-C2	1.416	1.417	1.427	1.428	1.421	1.422
C2-C3	1.416	1.417	1.427	1.428	1.421	1.423
C3-C4	1.416	1.417	1.427	1.428	1.421	1.423
C4-C5	1.416	1.417	1.427	1.428	1.421	1.423
C1-C5	1.416	1.417	1.427	1.428	1.421	1.423
C6-C7	1.401	1.401	1.411	1.412	1.406	1.407
C7-C8	1.400	1.400	1.410	1.411	1.405	1.407
C8-C9	1.400	1.401	1.411	1.412	1.406	1.408
C9-C10	1.401	1.401	1.412	1.412	1.407	1.408
C10-C11	1.400	1.401	1.411	1.412	1.406	1.407
C11-C12	1.400	1.400	1.410	1.411	1.405	1.407
C6-C12	1.401	1.401	1.411	1.412	1.406	1.408
Cp	η^5	η^5	η^5	η^5	η^5	η^5
Cht	η^7	η^7	η^7	η^7	η^7	η^7

Table S2. Structural parameters of **[9]**$^-$ when calculated with various density functionals. All structures were fully optimized with the def2-TZVPP basis set.

	PBE0-D3				BP86-D3				ωB97x-d			
	doublet		quartet		doublet		quartet		doublet		quartet	
	gas	DMSO	gas	DMSO	gas	DMSO	gas	DMSO	gas	DMSO	gas	DMSO
Mn-Cp	1.774	1.771	2.083	2.171	1.757	1.752	1.901	1.895	1.948	1.944	2.118	2.135
Mn-Cht	1.451	1.669	1.638	1.780	1.487	1.481	1.616	1.609	1.454	1.447	1.629	1.605
Mn-C1	2.157	2.201	2.406	2.473	2.117	2.152	2.276	2.274	2.260	2.308	2.432	2.451
Mn-C2	2.104	2.149	2.405	2.467	2.155	2.153	2.211	2.241	2.316	2.251	2.432	2.452
Mn-C3	2.104	2.102	2.404	2.484	2.156	2.121	2.209	2.222	2.261	2.314	2.437	2.451
Mn-C4	2.157	2.109	2.405	2.500	2.128	2.122	2.274	2.246	2.303	2.273	2.439	2.451
Mn-C5	2.207	2.160	2.406	2.493	2.126	2.119	2.317	2.277	2.304	2.288	2.436	2.452
Mn-C6	2.099	2.077	2.307	2.420	2.152	2.244	2.241	2.236	2.154	2.151	2.305	2.286
Mn-C7	2.869	2.099	2.308	2.385	2.234	2.480	2.831	2.810	2.169	2.158	2.305	2.285
Mn-C8	2.870	2.872	2.308	2.373	2.487	2.243	3.106	3.075	2.183	2.185	2.301	2.285
Mn-C9	2.099	2.872	2.308	2.391	2.275	2.163	2.833	2.809	2.159	2.169	2.298	2.287
Mn-C10	2.080	2.100	2.309	2.427	2.174	2.091	2.237	2.237	2.158	2.155	2.298	2.287
Mn-C11	2.114	2.078	2.307	2.454	2.092	2.091	2.033	2.032	2.208	2.196	2.299	2.285
Mn-C12	2.080	2.113	2.306	2.451	2.089	2.162	2.033	2.032	2.197	2.194	2.302	2.285
C1-C2	1.421	1.413	1.414	1.418	1.436	1.419	1.429	1.429	1.409	1.424	1.413	1.415
C2-C3	1.417	1.424	1.415	1.418	1.417	1.436	1.430	1.430	1.409	1.418	1.413	1.415
C3-C4	1.422	1.419	1.414	1.417	1.435	1.429	1.430	1.429	1.427	1.406	1.413	1.415
C4-C5	1.410	1.421	1.414	1.417	1.427	1.429	1.424	1.429	1.397	1.430	1.413	1.415
C1-C5	1.410	1.411	1.414	1.417	1.429	1.436	1.424	1.428	1.427	1.402	1.413	1.415
C6-C7	1.478	1.412	1.411	1.415	1.413	1.421	1.419	1.421	1.407	1.409	1.410	1.412
C7-C8	1.327	1.480	1.411	1.415	1.422	1.422	1.402	1.403	1.402	1.405	1.410	1.412
C8-C9	1.478	1.329	1.411	1.415	1.413	1.415	1.400	1.403	1.404	1.403	1.410	1.412
C9-C10	1.410	1.479	1.411	1.414	1.416	1.425	1.420	1.421	1.407	1.409	1.410	1.412
C10-C11	1.413	1.412	1.411	1.413	1.422	1.416	1.436	1.436	1.406	1.408	1.410	1.412
C11-C12	1.413	1.414	1.411	1.413	1.415	1.425	1.430	1.434	1.398	1.400	1.410	1.412
C6-C12	1.410	1.414	1.411	1.413	1.424	1.416	1.436	1.436	1.408	1.409	1.410	1.412
Hapticity	η^5	η^5	η^7	η^7	η^6	η^6	η^4	η^4	η^7	η^7	η^7	η^7
$\Delta E_{d\text{-}q}$	0.0	0.0	-50.5	-52.9	0.0	0.0	17.7	17.5	0.0	0.0	-48.9	-53.5
$\Delta G_{d\text{-}q}^{298K}$	0.0	0.0	-66.5	-61.6	0.0	0.0	5.6	6.1	0.0	0.0	-59.4	-61.8

The structure and the doublet-quartet energy gap is found to heavily depend on the density functional.

To analyse the charge distribution over **9** and its reduced from **[9]**$^-$, Hirshfeld population analyses were performed as listed in Table S3. The atom numbering corresponds to Figure S62.

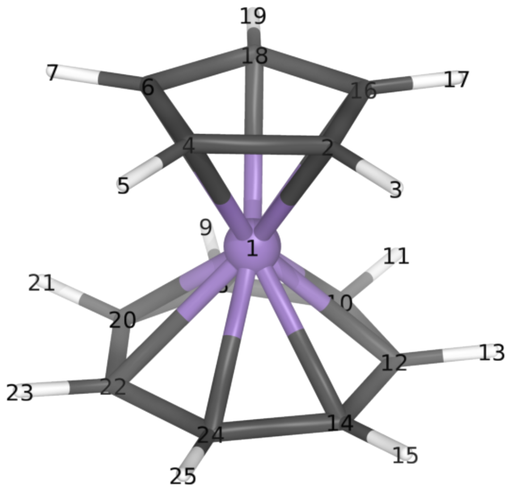

Figure S62. Atom numbering of **9** and **[9]**$^-$.

Table S3. Calculated Hirshfeld charges of **9** and **[9]**$^{-}$ obtained with PBE0-D3/def2-TZVPP in the gas phase.

	**9**	**[9]**$^{-}$	
		doublet	quartet
Mn1	0.143	0.132	0.206
C2	-0.030	-0.060	-0.052
H3	0.075	0.056	0.055
C4	-0.030	-0.059	-0.081
H5	0.075	0.059	0.049
C6	-0.030	-0.059	-0.067
H7	0.075	0.059	0.055
C8	0.005	-0.049	-0.086
H9	0.084	0.051	0.036
C10	0.007	-0.045	-0.064
H11	0.085	0.052	0.048
C12	0.006	-0.049	-0.033
H13	0.084	0.051	0.055
C14	0.005	-0.090	-0.065
H15	0.084	0.035	0.048
C16	-0.030	-0.047	-0.080
H17	0.075	0.057	0.049
C18	-0.030	-0.060	-0.063
H19	0.075	0.056	0.057
C20	0.005	-0.090	-0.048
H21	0.084	0.035	0.039
C22	0.007	-0.051	-0.048
H23	0.085	0.033	0.039
C24	0.006	-0.051	-0.085
H24	0.084	0.033	0.036

Frontier molecular orbitals

For sake of comparison frontier molecular orbitals were calculated with various density functionals as depicted in Figures S63-S64.

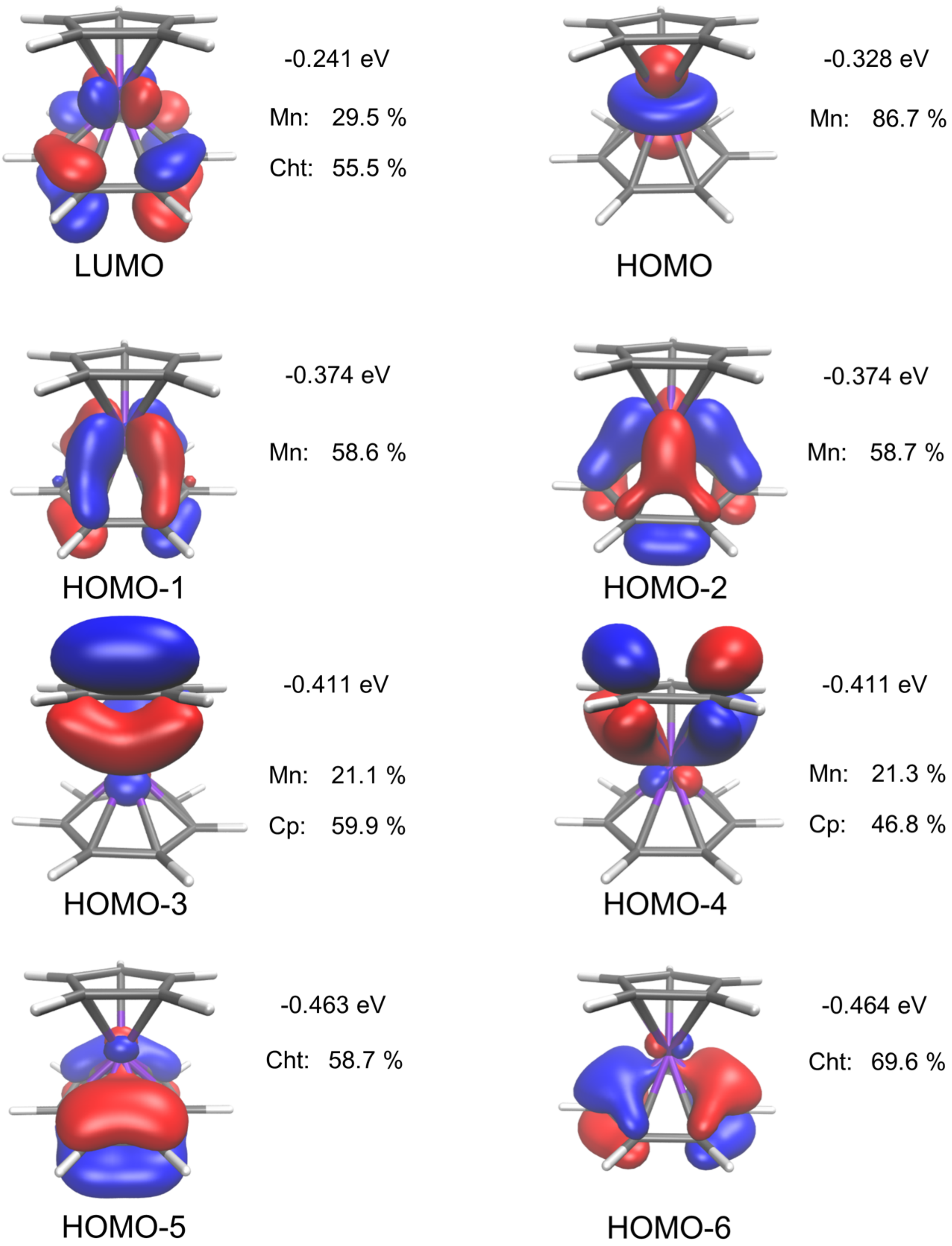

Figure S63. Frontier molecular orbitals of **9** as obtained with BP86-D3/def2-TZVPP.

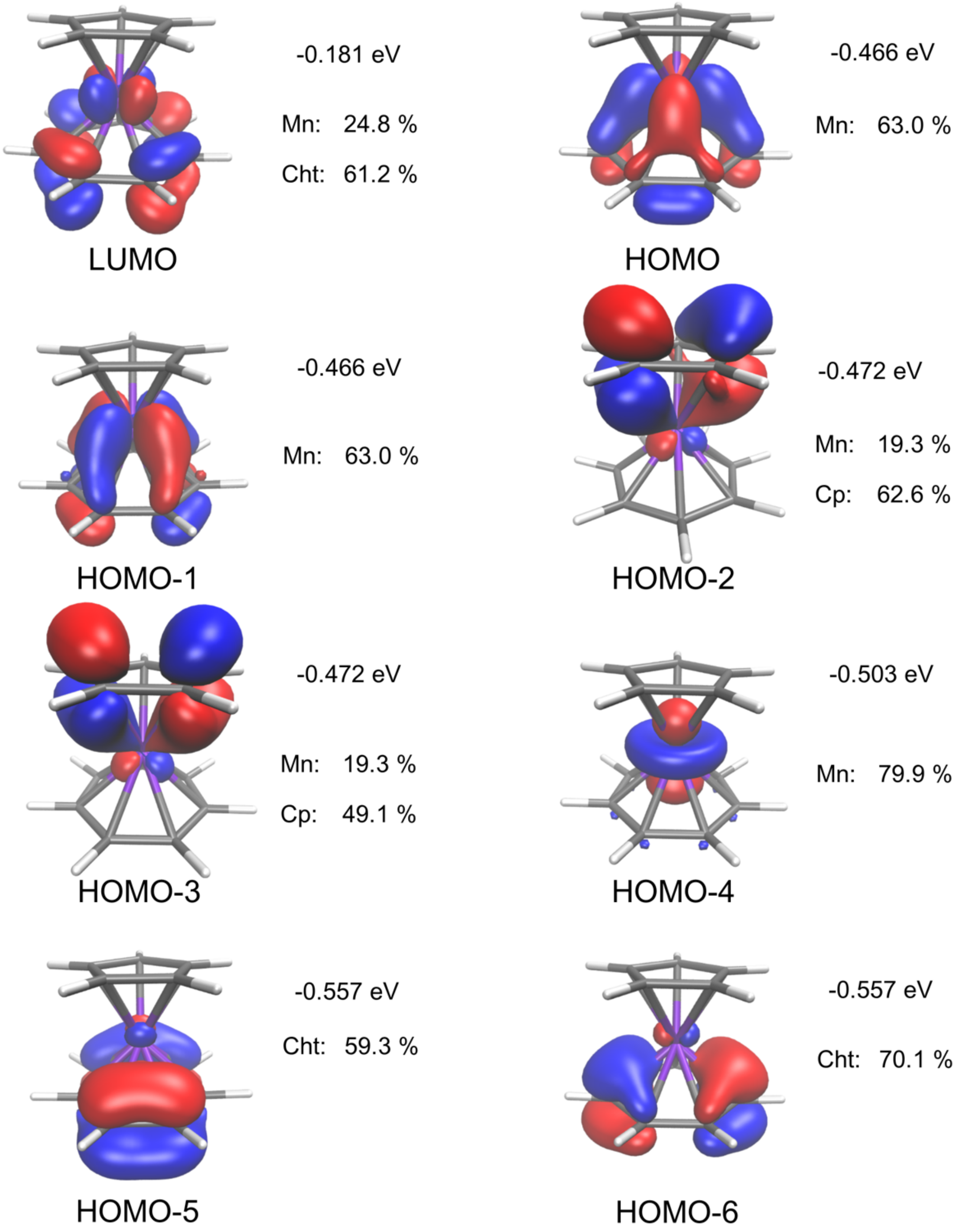

Figure S64. Frontier molecular orbitals of **9** as obtained with M05-2X/def2-TZVPP.

Calculation of p*K*a values

To estimate the basicity of tromancenium **9**, pK_a values in solution were calculated, whereas the solvent was modelled implicitly with the COSMO approach.

pK_a values were obtained from the Gibbs free energy of the solvation process $\Delta G^{\mathrm{Rxn}}{}_{(\mathrm{solv})}$ according to

$$\mathrm{p}K_a = \frac{\Delta G^{\mathrm{Rxn}}{}_{(\mathrm{solv})}}{RT\ln(10)} \qquad (1)$$

where R is here the universal gas constant and T the temperature. $\Delta G^{\mathrm{Rxn}}{}_{(\mathrm{solv})}$ can conveniently be estimated *via* the thermodynamic cycle depicted in Scheme S1, when calculating the gas phase reaction free energy $\Delta G^{\mathrm{Rxn}}{}_{(\mathrm{g})}$ and the free energies of solvation for each species, $\Delta G_{(\mathrm{solv})}$ (HA), $\Delta G_{(\mathrm{solv})}$ (H^+), and $\Delta G_{(\mathrm{solv})}$ (A^-), $\Delta G^{\mathrm{Rxn}}{}_{(\mathrm{solv})}$. In our case, $\Delta G_{(\mathrm{solv})}$ (HA) equals $\Delta G_{(\mathrm{solv})}$ of **9**, whereas $\Delta G_{(\mathrm{solv})}$ (A^-) is $\Delta G_{(\mathrm{solv})}$ of the deprotonated species **9**$^-$, where we took deprotonation at Cp and Cht into consideration. For free energy of solvation of H^+, $\Delta G_{(\mathrm{solv})}$ (H^+),[1] the literature value of -259.80 kcal/mol was used. The phase gas free energy of H^+ was calculated from the obtained from the Sackur-Tetrode equation and translational energy at 298 K and amounts to $G_{(\mathrm{g})}$ (H^+) = -6.29 kcal/mol. [2]

pK_a values were calculated for both density functionals (BP86 and PBE0).

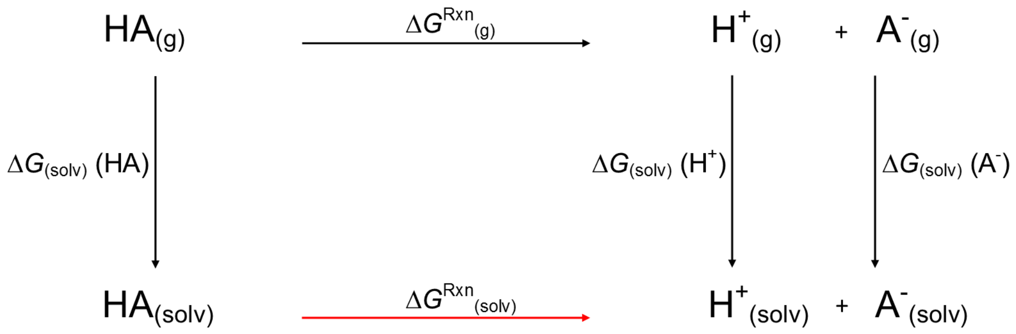

Scheme S1. Thermodynamic cycle used for the calculation of p*K*a values. The property of interest $\Delta G^{\mathrm{Rxn}}{}_{(\mathrm{solv})}$ (depicted by the red arrow) is calculated by the gas phase reaction free energy and the solvation free energies of each species.

Table S4. p*K*a values of **9** for Cp and Cht deprotonation when calculated with the BP86 and the PBE0 density functional in conjunction with the def2-TZVPP basis set.

Density Functional	p*K*a(Cp)	p*K*a(Cht)
BP86	42.5	40.9
PBE0	45.5	42.9
Average	44 ± 2	42 ± 1

Spin Density

The spin densities of **[9]**⁻ in the two potential spin states calculated with various density functionals are depicted in Figures S65-S67.

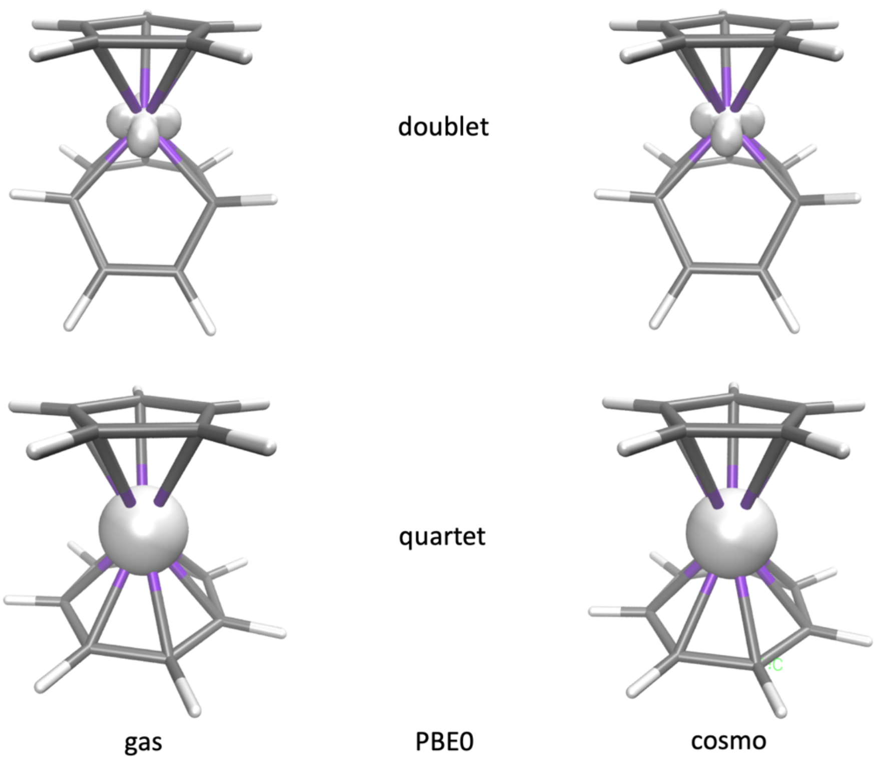

Figure S65. Spin density of **[9]**⁻ in the doublet spin state (top panel) and in the quartet spin state (bottom panel) calculated with PBE0-D/def2-TZVPP.

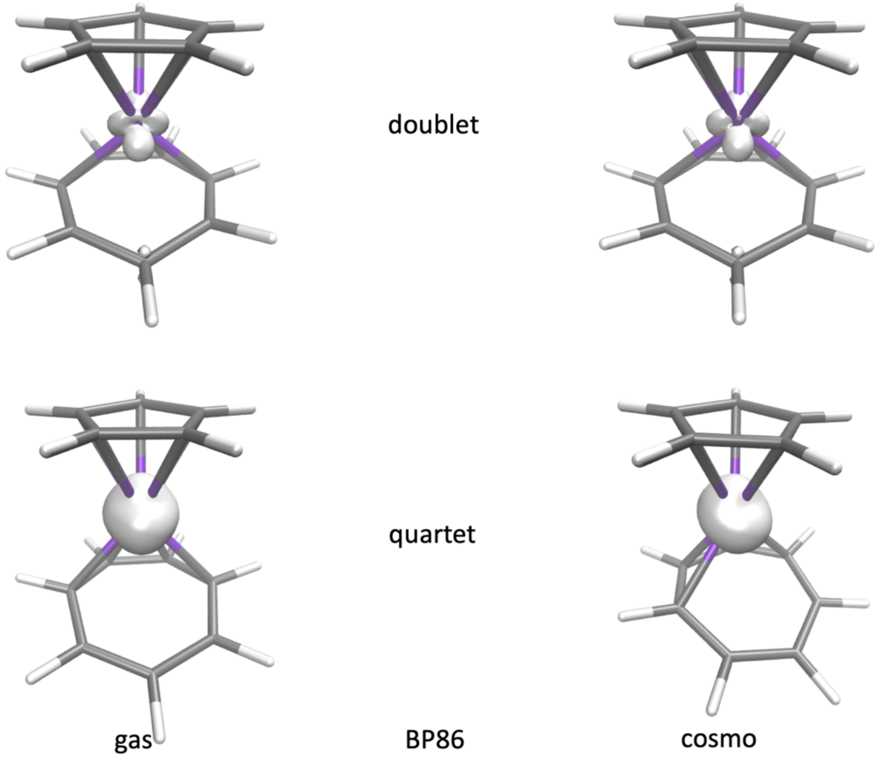

Figure S66. Spin density of **[9]**$^{-}$ in the doublet spin state (top panel) and in the quartet spin state (bottom panel) calculated with BP86-D3/def2-TZVPP.

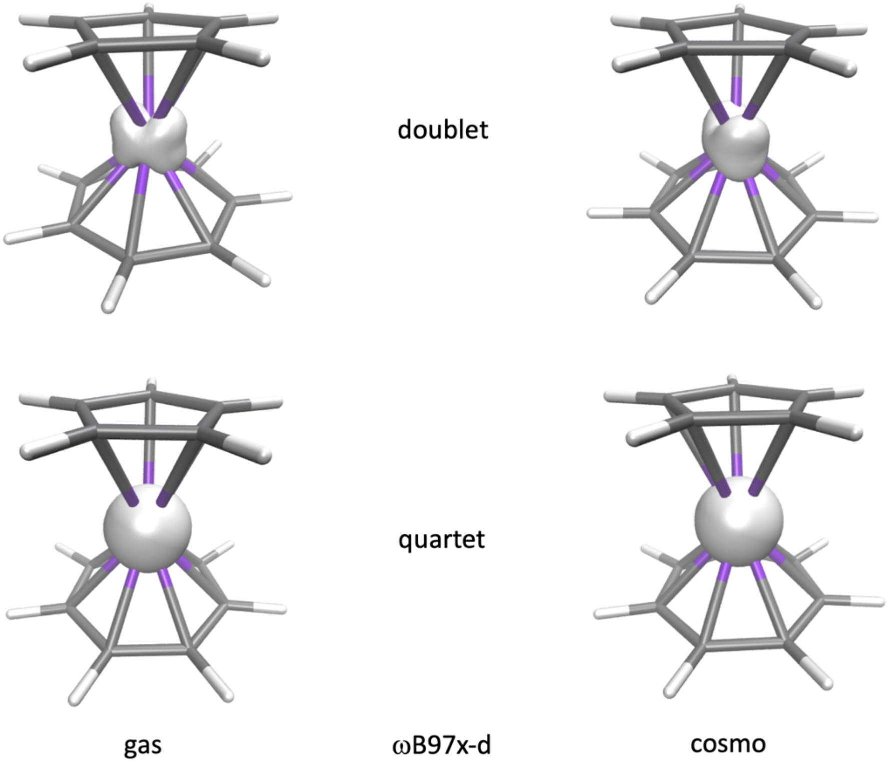

Figure S67. Spin density of **[9]**$^-$ in the doublet spin state (top panel) and in the quartet spin state (bottom panel) calculated with ωB97x-d/def2-TZVPP.

References

(1) Single-Ion Solvation. Experimental and Theoretical Approaches to Elusive Thermodynamic Quantities. P. Hünenberger, M. Reif, Royal Society of Chemistry - Theoretical and Computational Chemistry Series London, UK, ISBN: 978-1-84755-187-0 (2011).

(2) See e.g., J. Ho, M. L. Coote, *Wiley Interdiscip. Rev. Comput. Mol. Sci.* **2011**, *1*, 649-660, and references therein.
